# Antiparkinson Drug Benztropine Suppresses Tumor Growth, Circulating Tumor Cells, and Metastasis by Acting on SLC6A3/DAT and Reducing STAT3

**DOI:** 10.3390/cancers12020523

**Published:** 2020-02-24

**Authors:** Chiharu Sogawa, Takanori Eguchi, Manh Tien Tran, Masayuki Ishige, Kilian Trin, Yuka Okusha, Eman Ahmed Taha, Yanyin Lu, Hotaka Kawai, Norio Sogawa, Masaharu Takigawa, Stuart K. Calderwood, Kuniaki Okamoto, Ken-ichi Kozaki

**Affiliations:** 1 Department of Dental Pharmacology, Graduate School of Medicine, Dentistry and Pharmaceutical Sciences, Okayama University, Okayama 700-8525, Japan; caoki@md.okayama-u.ac.jp (C.S.); trantienmanh1508@gmail.com (M.T.T.); kilian.trin56@hotmail.fr (K.T.); yokusha@bidmc.harvard.edu (Y.O.); pj7l8pfb@s.okayama-u.ac.jp (E.A.T.); riku21@s.okayama-u.ac.jp (Y.L.); k-oka@okayama-u.ac.jp (K.O.); ken-1@okayama-u.ac.jp (K.-i.K.); 2 Advanced Research Center for Oral and Craniofacial Sciences, Graduate School of Medicine, Dentistry and Pharmaceutical Sciences, Okayama University, Okayama 700-8525, Japan; takigawa@md.okayama-u.ac.jp; 3 On-Chip Biotechnologies, Co., Ltd. Tokyo 184-0012, Japan; m-ishige@on-chip.co.jp; 4 ODAPUS (Okayama University Dental School Short-Term-Study-Abroad Program for Undergraduate Students) from Medical School, University of Western Brittany, 29238 Brest, France; 5 Department of Radiation Oncology, Beth Israel Deaconess Medical Center, Harvard Medical School, Boston, MA 02115, USA; scalderw@bidmc.harvard.edu; 6 Department of Medical Bioengineering, Graduate School of Natural Science and Technology, Okayama University, Okayama 700-8530, Japan; 7 Department of Oral Pathology and Medicine, Graduate School of Medicine, Dentistry, and Pharmaceutical Sciences, Okayama University, Okayama 700-8525, Japan; de18018@s.okayama-u.ac.jp; 8 Department of Dental Pharmacology, Matsumoto Dental University, Shiojiri 399-0704, Japan; sogawa@po.mdu.ac.jp

**Keywords:** drug repositioning/repurposing, three-dimensional (3D) culture, tumoroids, dopamine transporter (DAT), benztropine, signal transducer and activator of transcription (STAT), circulating tumor cell (CTC)

## Abstract

Tumor growth, progression, and therapy resistance are crucial factors in the prognosis of cancer. The properties of three-dimensional (3D) tumor-like organoids (tumoroids) more closely resemble in vivo tumors compared to two-dimensionally cultured cells and are therefore effectively used for assays and drug screening. We here established a repurposed drug for novel anticancer research and therapeutics using a 3D tumoroid-based screening system. We screened six pharmacologically active compounds by using an original tumoroid-based multiplex phenotypic screening system with a matrix metalloproteinase 9 (MMP9) promoter-driven fluorescence reporter for the evaluation of both tumoroid formation and progression. The antiparkinson drug benztropine was the most effective compound uncovered by the screen. Benztropine significantly inhibited in vitro tumoroid formation, cancer cell survival, and MMP9 promoter activity. Benztropine also reduced the activity of oncogenic signaling transducers and trans-activators for MMP9, including STAT3, NF-κB, and β-catenin, and the properties of cancer stem cells/cancer-initiating cells. Benztropine and GBR-12935 directly targeted the dopamine transporter DAT/SLC6A3, whose genetic alterations such as amplification were correlated with poor prognosis for cancer patients. Benztropine also inhibited the tumor growth, circulating tumor cell (CTC) number, and rate of metastasis in a tumor allograft model in mice. In conclusion, we propose the repurposing of benztropine for anticancer research and therapeutics that can suppress tumor progression, CTC, and metastasis of aggressive cancers by reducing key pro-tumorigenic factors.

## 1. Introduction

Cancer is one of the most severe diseases throughout the world, and tumor metastasis and therapy resistance are leading causes of death. Poor prognosis in cancer is associated with the dissemination of tumor cells from a primary lesion to the blood circulation and to distant organs where metastatic secondary tumors are formed [1]. A number of studies have shown that matrix metalloproteinases (MMP), especially MMP9, were often highly expressed in human cancers and correlated with the poor prognosis of patients [2,3]. The canonical functions of MMPs involve their roles as extracellular proteases, promoting tumor progression by enhancing tumor growth, migration, invasion, angiogenesis, and metastasis of tumor cells [4,5]. However, we have recently re-defined MMPs as moonlighting metalloproteinase inasmuch as intranuclear MMP can play roles in the transcriptional regulation involved in tumor progression and cartilage metabolism [4,6,7]. Thus, the mechanisms of expression of MMPs are important in cancer progression and therapies. The promoter regions of the MMP9 gene in humans and mice contain binding sites for a number of oncogenic factors including the signal transducer and activator of transcription (STAT), TCF/LEF/β-catenin transcriptional complex, and NF-κB, which are all crucial in cancer cell survival and in the properties of cancer stem cells (CSC), also known as cancer-initiating cells (CIC) [8,9,10]. It has been shown that tumors include CIC/CSC populations, cell fractions essential for tumorigenesis, recurrence, and metastatic potential [11]. Therefore, in the present study, MMP9 expression is a potent marker of tumor progression, recurrence, and metastatic potential, which we aimed to suppress.

Drug repositioning, also known as drug repurposing, involves the investigation and utilization of existing drugs for new therapeutic purposes/indications [12]. The pharmacologist and Nobel laureate James Black said, “the most fruitful basis for the discovery of a new drug is to start with an old drug” [13]. Medicinal effects, adverse effects, and the therapeutic index of existing drugs are well clarified. Therefore, drug repositioning eliminates much of the toxicological and pharmacokinetic assessment required for the development of new drugs [13]. We recently proposed a potent drug repurposing of anti-malaria drug artesunate (ART) for an anticancer agent that effectively reduced both the viability of a tumor-like organoid (tumoroid) and the activities of the MMP9 promoter [2]. This study was an original proof of the concept of how the multiplex tumoroid reporter system could be useful in drug selection. Success in anticancer drug discovery depends heavily on utilizing the appropriate experimental tumor models and screening system. In this respect, three-dimensional (3D) organoid/tissue culture systems can replicate many aspects of 3D organs, tumors, and their environment and are thus more suitable for many physiological and pathological studies [14]. The formation of 3D tumorspheres also known as spheroids had been an indicator of the tumor-initiating properties of CSC/CIC [15,16]. Thus, a 3D culture system has enabled us to develop a tumoroid system *in vitro*, corresponding closely to the properties of native tumors *in vivo* [16,17]. Using the tumoroid system, we have identified a novel effect of a CDK2 inhibitor on suppressing epithelial-mesenchymal transition (EMT) in cancer [18]. We have also developed an original 3D tumoroid-based multiplex assay system with a MMP9 promoter-driven fluorescence reporter [2] and used this system in the present study for drug selection and for the evaluation of both tumoroid formation and progression. Our previous drug screening inspired us to further select pharmacologically active compounds that potentially reduce the cancer cell viability in the 3D tumoroids. In the present study, we examined six pharmacologically active compounds, including ART, AL8810 (prostagrandin F2α analogues) [19], 2-Amino-5-phosphonopentanoic acid [AP5: N-methyl-D-aspartate (NMDA) receptor antagonist] [20], Chlorzoxazone (muscle relaxant) [21], Carboplatin (platinum anticancer drug) [22], and Benztropine mesylate (Benz; antiparkinson drug). Among them, Benz was the most effective hit. Benztropine (Cogentin®) is currently a second-line drug for the treatment of Parkinson’s disease, also used for the treatment of dystonia [12]. Benz had been known to improve Parkinson’s symptoms, mainly as a muscarinic M1 antagonist, as well as a dopamine reuptake inhibitor (DRI), which blocks the action of the dopamine transporter [DAT, also known as solute carrier family 6 member 3 (SLC6A3)] [23,24]. Additionally, Benz also has affinities with other membrane proteins, including dopamine receptors, histamine receptors, and the norepinephrine transporter (NET) [25,26]. 

In the present study, we thus aimed: (i) to screen potential repurposing drugs for anticancer therapy by using the 3D tumoroid multiplex reporter system, (ii) to investigate whether one compound, Benz, could act as an anticancer agent suppressing tumor progression in vitro and in vivo, and reducing circulating tumor cells and metastasis, and (iii) to reveal a mechanism of action (MoA) of Benz in targeting cancer cells by acting on cell surface molecules and by altering key pro-tumorigenic signaling transactivators such as STAT, NF-κB, and β-catenin. 

## 2. Results

### 2.1. Multiplex Drug screening to Target 3D Tumorigenicity, MMP9 Promoter Activity, and Cancer Cell Viability

**Figure 1:**
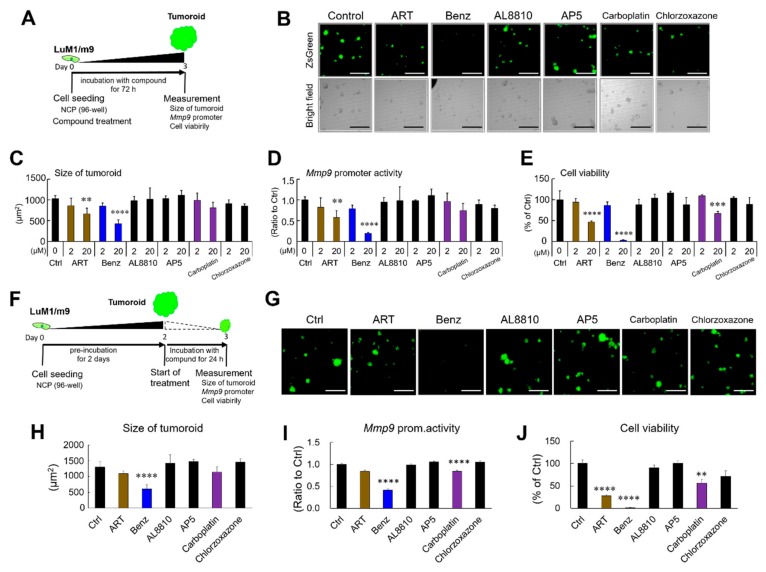
Tumoroid-based multiplex drug screening to target matrix metalloproteinase 9 (MMP9) promoter activity and cancer cell viability. (**A**) Schematic representation of the experimental system. LuM1/m9 reporter cells were cultured to form tumoroids in 96-well NanoCulture Plate (NCP) for 72 h with or without 2 or 20 μM Benz, artesunate (ART), AL8810, AP5, carboplatin, and chlorzoxazone. (**B**) Representative images of tumoroids with reporter fluorescence (top) and bright fields (bottom) after 72 h treated with 20 μM of each compound. Scale bars, 500 μM. (**C**) Tumoroid size altered by the compounds. The size of tumoroids was measured using an ArrayScan HCS system. (**D**) Relative activities of the MMP9 promoter. (**E**) Cell viabilities altered by the compounds. The cellular ATP contents were evaluated as cell viability. (**F**) Schematic representation of the preformed tumoroid-based experiments in panels G to J. The preformed tumoroids were treated with 100 μM of each compound for 24 h. (**G**) Representative images of tumoroids with reporter fluorescence after 24 h treatment with 100 μM of each compound. Scale bars, 500 μM. (**H**) Size of tumoroids altered by the compounds. The size of an area greater than 300 μm^2^ was counted as a tumoroid. (**I**) MMP9 promoter activities. (**J**) Cell viabilities altered by 100 μM of each compound. The values were shown as the average value of 3 or 4 wells. Mean ± SD. ** *P* < 0.01, *** *P* < 0.001, and **** *P* < 0.0001 (vs. Ctrl).

In order for the discovery of a novel anticancer drug inspired by a concept of drug repositioning, we examined six pharmacologically active compounds that potentially might reduce the viability of the 3D tumoroids, composed of Benz, AL8810, AP5, ART, chlorzoxazone, and carboplatin. We examined whether these compounds could suppress tumoroid growth, MMP9 promoter activities, and cancer cell viabilities by using the tumoroid-based multiplex phenotypic screening system. Benz and ART at concentrations of 20 µM significantly decreased tumoroid formation, MMP9 promoter activity, and viability of the LuM1/m9 reporter cells, while Benz was more effective than ART (Figure 1A–E). At 20 µM, the established compound carboplatin, a platinum-based anticancer drug, also decreased cancer cell viability but did not suppress tumoroid formation and the MMP9 promoter. The surviving LuM1/m9 reporter cells were ZsGreen-positive, while ZsGreen-negative debris of dead cells were seen under the bright field (Figure 1B).

Next, we examined whether these drugs could target preformed tumoroids. For this purpose, tumoroids were preformed by culturing LuM1/m9 reporter cells for two days before the administration of the drugs. Benz was the only drug that significantly impacted the tumoroids (Figure 1F–H), reduced MMP9 promoter activity, and reduced tumoroid viability (Figure 1I,J). Carboplatin reduced the MMP9 promoter activity and cell viability, although it failed to impact the size of the tumoroids. ART decreased the viability of the tumoroids but failed to target either the structure of the tumoroids or the MMP9 promoter activity.

Thus, the tumoroid-based multiplex screening identified Benz, a compound that could potentially target tumorigenicity, MMP-driven metastatic potential, and cancer cell survival.

### 2.2. Benztropine Suppresses Tumoroid Viability and MMP9 Promoter Activity

**Figure 2:**
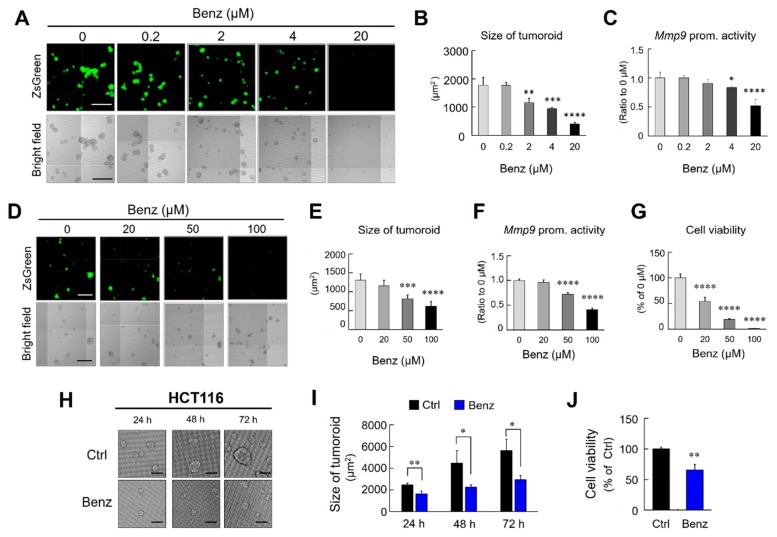
**Inhibitory effects of Benztropine on tumoroids formed by mouse- and human-derived colon cancer cells.** (A to C) LuM1/m9 cells were treated with Benz (0, 0.2, 2, 4, and 20 μM) for 72 h. (**A**) Representative images of tumoroids with LuM1/m9 reporter fluorescence (top) and bright fields (bottom). Scale bars, 500 μm. (**B**) Size of tumoroids altered by treatment with Benz. (**C**) MMP9 promoter activities altered by Benz. (**D**–**G**) Preformed tumoroids of LuM1/m9 cells were treated with Benz (0, 20, 50, and 100 μM) for 24 h. (**D**) Representative images of tumoroids with LuM1/m9 reporter fluorescence. Scale bars, 500 μm. The size of an area greater than 300 μm^2^ was counted as a tumoroid. The (**E**) size, (**F**) MMP9 promoter activities, and (**G**) cell viability of tumoroids were decreased after treatment with Benz. The values were shown as the average value of 4 wells. Mean ± SD. * *P* < 0.05, ** *P* < 0.01, *** *P* < 0.001, **** *P* < 0.0001 (vs. untreated group). From the data, IC_50_ of Benz was 16.5 μM. (**H–J**) The effects of Benz on tumoroid formation in human colon cancer cells. A human colon cancer cell line HCT116 cultured in the 3D culture system was treated with 20 μM Benz for 72 h. (**H**) Representative images of tumoroids treated with Benz for 24 h, 48 h, and 72 h. Scale bars, 100 μm. (**I**) The size of tumoroids altered by treatment with Benz. The size of 33 tumoroids in each well was measured. Mean ± SD, n = 3 wells. (**J**) Tumoroid cell viability evaluated by cellular ATP content. The values were shown as % of the control. Mean ± SD, n = 3 wells. * *P* < 0.05, ** *P* < 0.01 (vs. untreated group).

We next asked whether Benz, at a broader range of concentrations, could suppress the viability of tumoroids formed by colon cancer cells derived from humans and mice. Exposure to Benz at 2 to 20 µM significantly inhibited the formation of tumoroids of mouse-derived LuM1/m9 cells (Figure 2A,B), although 0.2 µM Benz was ineffective. Benz at 4 and 20 µM inhibited the MMP9 promoter activity in a dose-dependent manner (Figure 2A,C), although 0.2 and 2 µM were ineffective. We next asked whether Benz could destruct preformed tumoroids and inhibit their MMP9 promoter. Benz at 50 to 100 µM significantly reduced the size of tumoroids in a dose-dependent manner (Figure 2D,E), although 20 µM was ineffective. Benz at 50 to 100 µM also significantly inhibited the MMP9 promoter in the preformed tumoroids in a dose-dependent manner (Figure 2D,F), although 20 µM was ineffective. Benz at 20, 50, and 100 μM significantly exerted cytotoxicity on the preformed tumoroids (Figure 2G), and, from the cytotoxicity data, IC_50_ of Benz was calculated to be 16.5 μM. These data suggest that Benz could be effectively cytotoxic on preformed tumors.

We next examined the cytotoxicity of Benz by measuring the release of lactate dehydrogenase (LDH), a marker of cell damage and death, from the LuM1 tumoroids. Benz at 20 μM induced the release of LDH from tumoroids for 48 to 72 hours treatment periods, but not for 24 hours (Figure S1). The release of LDH was concomitant with the significant loss in cancer cell viability for the 48 to 72 hours treatment periods.

We next examined the effects of Benz on tumoroid formation by the human colon cancer cell line, HCT116 cells. Benz at 20 μM significantly inhibited the tumoroid growth of HCT116 for 24, 48, and 72 hours treatment periods (Figure 2H,I). Benz at 20 μM significantly inhibited tumoroid growth of HCT116 for 24, 48, and 72 hours treatment periods (Figure 2H,I). Concomitantly, Benz at 20 μM significantly exerted cytotoxicity on tumoroids of HCT116 cells (Figure 2J). 

These data indicate that Benz is cytotoxic to 3D tumoroids and powerfully suppresses tumoroid growth and metalloproteinase promoter activity.

### 2.3. Benztropine Treatment Suppresses Migration and Invasion of Metastatic Cancer Cells

**Figure 3:**
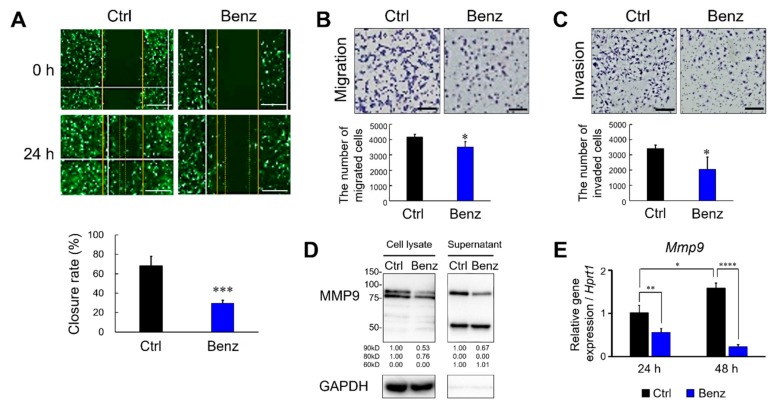
**Suppressive actions of Benztropine on the migration and invasion of cancer cells.** (**A**) Wound healing assay. The solid lines indicate the initial wounds. The dotted lines indicate the leading edge of the closing wound. Upper panel, representative images. Lower panel, rate of the wound closure. Mean ± SD, n = 4. *** *P* < 0.001 vs Ctrl. (**B**,**C**) Migration and invasion assays using a transwell system. LuM1 cells were treated with Benz (50 μM) for 24 h. The (**B**) migrated and (**C**) invaded cells were stained with Diff-Quick and then counted. Upper panels, (**B**) representative images of the migrated cells and (**C**) invaded cells. Scale bars, 100 μm. Lower panels, the number of cells migrated or invaded in a well. Mean ± SD, n = 3. * *P* < 0.05 vs Ctrl. (**D**) Western blot showing MMP9 altered by Benz treatment. LuM1 cells were treated with Benz at 50 μM for 24 h. Whole cell lysate and culture supernatant were prepared from the cells cultured in a serum-free medium for 24 h. Bands of approximately 90 kD (pro-form), 80 kD (active-form), and 60 kD (short-form) of MMP9 were detected. GAPDH, loading control. Densitometric readings for all western blots were calculated relative to the Ctrl. (**E**) RT-qPCR analysis for MMP9 mRNA levels. LuM1 cells were treated with 20 μM Benz for 24 h and 48 h under 3D culture condition. Mean ± SD, n = 3. * *P* < 0.05, ** *P* < 0.01, and **** *P* < 0.0001 vs Ctrl. The mRNA levels were normalized with an internal control *Hprt1*.

Next, we examined whether Benz could suppress cancer cell motility and invasiveness driven by the expression of MMP9. For the migration assay, the LuM1/m9 reporter cells were cultured to be confluent in a 2D culture condition. In the 2D condition, the administration of Benz at 100 μM for 24 hours was cytotoxic, while that at 50 μM for 24 hours was not (data not shown). Benz pre-treatment (at 50 μM for 24 hours) significantly suppressed wound closure by migrating LuM1/m9 cells (Figure 3A). Benz also attenuated LuM1 cell migration in a transwell system (Figure 3B) and inhibited the invasion of LuM1 cells in the Matrigel (Figure 3C). 

Next, we investigated whether the protein and transcript levels of MMP9 could be lowered by Benz treatment. Benz treatment at 50 μM for 24 hours markedly reduced both intracellular and extracellular MMP9 of LuM1 cells (Figure 3D, Figure S2). The expression level of MMP9 mRNA in tumoroids was significantly lowered by Benz treatment at 20 µM for 24 to 48 hours (Figure 3E). Thus, it was indicated that Benz inhibited the transcription and production of MMP9 from the metastatic cancer cells. 

These data indicate that Benz inhibits cancer cell migration and invasion by suppressing MMP9 expression.

### 2.4. Benztropine Treatment Reduces STAT, NF-κB, β-catenin, and CD326 in Tumoroids

**Figure 4:**
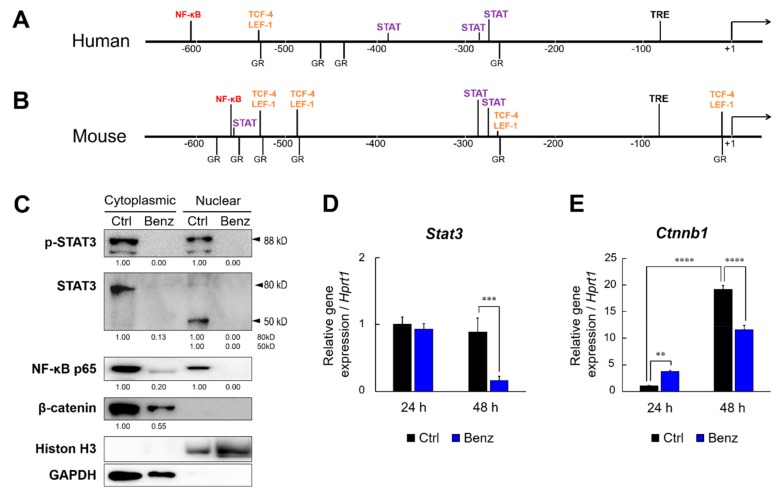
Benztropine reduces STAT, NF-κB, and β-catenin in tumoroids. (A,B) Maps of MMP9 promoter regions in (**A**) humans and (**B**) mice. Binding sites for STAT, NF-κB, TCF-4/LEF-1, and the glucocorticoid receptor (GR) were mapped. (**C**) Western blot showing STAT3, NF-κB p65/RelA, and β-catenin in nuclear and cytoplasmic fractions of LuM1 tumoroids. Tumoroids were treated with 20 μM Benz for 72 h. Phosphorylated STAT3 (p-STAT3) and unphosphorylated STAT3 were examined. Histone H3, a nuclear marker. GAPDH, a cytoplasmic marker. Densitometric readings for all western blots were calculated relative to the Ctrl. (**D, E**) RT-qPCR analysis for the mRNA levels of Stat3 and Ctnnb1 altered by Benz. LuM1 cells were treated with 20 μM Benz for 24 h and 48 h under 3D culture condition. Mean ± SD, n = 3. ** *P* < 0.01, *** *P* < 0.001 and **** *P* < 0.0001 vs Ctrl. mRNA levels were normalized with Hprt1 mRNA.

The promoter regions of MMP9 contain binding sites for oncogenic transcription factors such as STAT, NF-κB, and TCF/LEF/β-catenin complex in humans and mice (Figure 4A,B, Figure S3). We therefore hypothesized that Benz could suppress MMP9 expression by regulating these transcription factors. The mouse MMP9 promoter region (from –600 to +20) contains three STAT binding sequences (preferring STAT1/3/4), among which two are conserved between humans and mice (Figure S3). The major isoforms of STAT3 are STAT3α (86 kD) and STAT3β (79 kD), while it has been reported that 50-kD STAT3 was generated by caspase 3-dependent cleavage [27]. Moreover, it has been also shown that unphosphorylated STAT3 (uSTAT3) is a functional trans-activator and able to form a uNF-κB/uSTAT3 heterodimer able to activate NF-κB target genes [28]. We investigated whether Benz treatment could alter the subcellular status of STAT3, NF-κB p65/RelA, and β-catenin in the tumoroids of metastatic LuM1 cells. An anti-phosphorylated STAT3 (p-STAT3) C-terminus antibody detected duplex bands in both cytoplasmic and nuclear fractions of the tumoroids, resembling the two major isoforms p-STAT3α (86 kD + phospho = 88 kD) and p-STAT3β (79 kD + phospho = 81 kD), whereas p-STAT3 was undetectable upon Benz treatment (Figure 4C, top, Figure S4A). The anti-STAT3 N-terminus antibody detected STAT3 (approx. 80 kD) in the cytoplasm, while a 50-kD short fragment of STAT3 (lacking C-terminal phosphorylation sites, resembling uSTAT3) was found only in cell nuclei of 3D tumoroids (Figure 4C, second raw, Figure S4B). Both 80-kD and 50-kD STAT3 disappeared from the tumoroids upon Benz treatment. NF-κB/RelA was detectable in both cytoplasmic and nuclear fractions of tumoroids, while RelA disappeared from the nuclei fraction upon Benz treatment and was markedly reduced in the cytoplasm by Benz treatment (Figure 4C, third raw, Figure S4C). 

It has been shown that CD326, also known as the epithelial cell adhesion molecule, could form a protein complex with β-catenin for promoting CSC/CIC properties essential for tumor progression, recurrence, and metastasis [29]. β-catenin was well detectable in the cytoplasmic fraction, but not in the nuclear fraction of the tumoroids (Figure 4C, fourth raw, Figure S4D). Benz treatment markedly decreased the protein level of β-catenin in the cytoplasm. CD326 was well detectable in the cytoplasmic fraction of the tumoroids, while CD326 disappeared from the tumoroids by Benz treatment (Figure S5A). These data indicated that Benz was able to reduce the levels of pro-tumorigenic factors in tumoroids, including STAT3, NF-κB, β-catenin, and CD326. 

Next, we asked whether the mRNA levels of STAT3 and β-catenin (encoded by *Ctnnb1* gene) could be altered by tumoroid growth and by Benz treatment. Benz treatment at 20 µM significantly lowered the *Stat3* mRNA level in the tumoroids (Figure 4D). Tumoroid growth significantly increased the *Ctnnb1* mRNA level from the 24 to 48 hours culture periods, although Benz treatment for 48 hours significantly inhibited the induction of *Ctnnb1* mRNA (Figure 4E). The mRNA levels of CD326, HIF-1α, and pluripotency genes including Nanog, Sox2, and Oct4 were increased along with tumoroid growth at 24 to 48 hours, although they were not lowered by Benz treatment (Figure S5B). 

These experiments show that Benz is specifically able to reduce STAT3, NF-κB, and β-catenin in the tumoroids.

### 2.5. Anti-Tumor Effect of Benztropine through Inhibition of Dopamine Transporter SLC6A3

**Figure 5:**
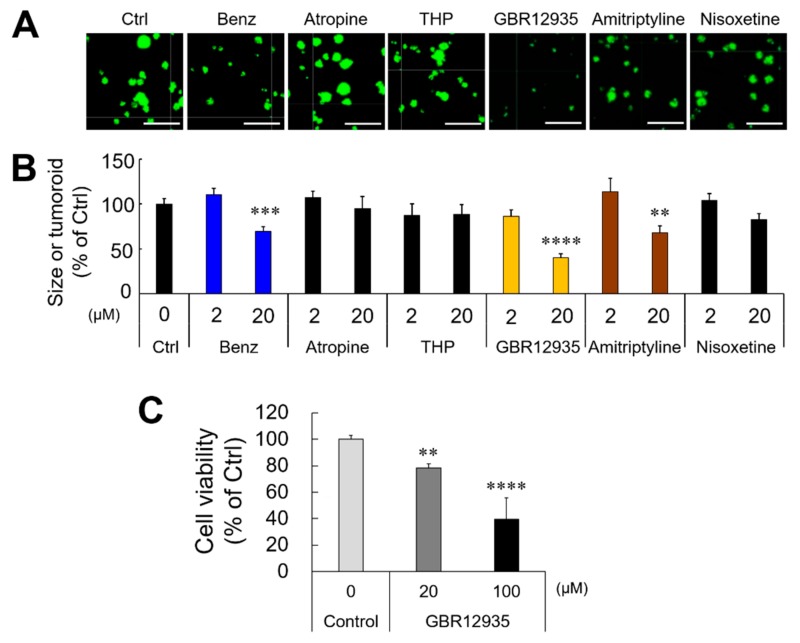
**Anti-tumor effect of Benztropine through the inhibition of the dopamine transporter SLC6A3.** (**A,B**) LuM1 tumoroids were treated with Benz, Atropine (muscarinic antagonist), Trihexyphenidyl (THP; muscarinic antagonist), GBR12935 (DAT inhibitor), amitriptyline (MAT inhibitor), and nisoxetine (NET inhibitor) for 72 hours. The protocol shown in Figure 1G was used. (**A**) Representative images of tumoroids with LuM1/m9 reporter fluorescence. Scale bars, 500 μm. (**B**) The size of the tumoroids altered by drugs. The values were shown as % of Ctrl. Mean ± SD, n = 4. ** *P* < 0.01, *** *P* < 0.001 and **** *P* < 0.0001 vs Ctrl. (**C**) Tumoroid cell viability evaluated by cellular ATP contents. LuM1 tumoroids were treated with 0, 20, or 100 μM GBR12935 for 24 h. The values were shown as % of Ctrl.

It has been shown that Benz improves Parkinson’s symptoms, mainly as a muscarinic M1 antagonist, as well as a DRI, which blocks the action of the DAT/SLC6A3 [24], while Benz also has affinities with other membrane proteins, including dopamine receptors, histamine receptors, and NET [25,26]. In order to investigate an anti-tumorigenic MoA of Benz based on its mechanisms in Parkinson’s, we treated the tumoroids with an antimuscarinic (atropine), an antiparkinsonian agent of the antimuscarinic class trihexyphenidyl (THP), a DAT inhibitor (GBR-12935), a NET inhibitor [nisoxetine; a tricyclic antidepressant (TCA)], and a non-specific MAT inhibitor (amitriptyline; TCA). The muscarinic antagonists (atropine and THP) did not alter tumoroid formation, while Benz inhibited tumoroid formation at 20 µM. GBR-12935 at 20 µM significantly inhibited tumoroid growth (Figure 5A,B). In contrast, the NET inhibitor nisoxetine did not alter tumoroid growth as compared to the untreated control. The MAT inhibitor amitriptyline significantly inhibited tumoroid growth, suggesting that DAT inhibition, but not NET inhibition, was required for tumoroid inhibition. GBR-12935 lowered the cancer cell viability of preformed tumoroids in a concentration-dependent manner (Figure 5C). Thus, DAT inhibition is a common mechanism by which Benz and GBR-12935 inhibit tumor growth.

It has been known that DAT inhibitors are DRI increasing extracellular dopamine and dopaminergic neurotransmission and signaling [24]. We therefore examined whether tumoroid growth and MMP9 promoter activities could be altered by dopamine and sulpiride, a selective dopamine receptor antagonist. Neither dopamine nor sulpiride altered tumoroid growth and MMP9 promoter activities (Figure S6). Neither dopamine nor sulpiride exerted any synergistic effect or antagonistic effect with the DAT inhibitors (Benz and GBR12935). These results suggest that the antitumor effects of DATs did not involve dopamine or dopamine receptors.

Thus, these data indicate that Benz inhibits tumor growth by acting on DAT and reducing STAT3, NF-κB/RelA, and β-catenin.

### 2.6. Benztropine Treatment Suppresses Tumor Growth, Circulating Tumor Cells, and Metastasis

**Figure 6:**
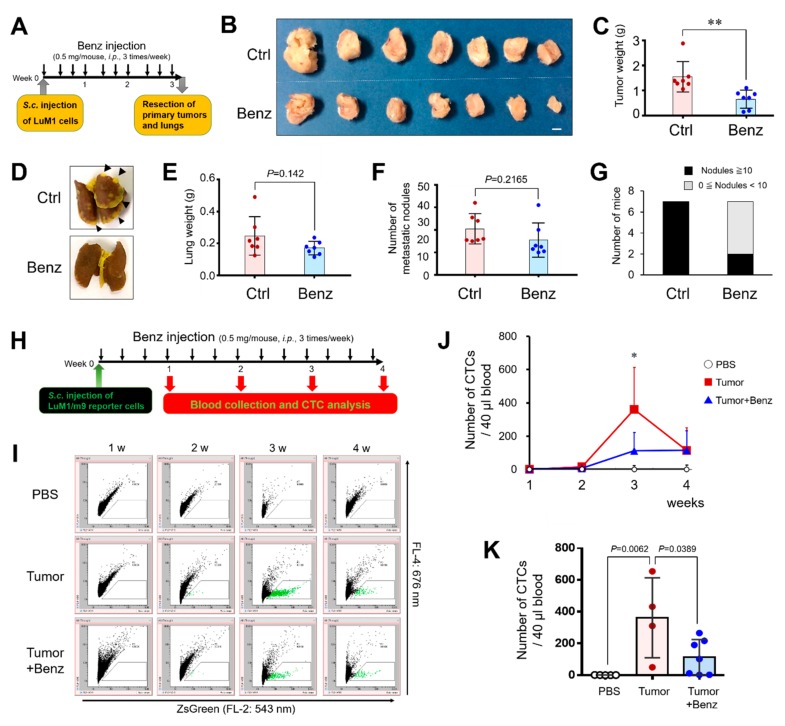
**Benztropine inhibits tumor growth, circulating tumor cells, and metastasis in vivo.** (**A** to **G**) Evaluation of tumor growth and metastasis. (**A**) Schedule of LuM1 tumor cell injection and Benz treatment to BALB/c mice. LuM1 (500k cells) was subcutaneously (s.c.) injected into the side abdominal wall of BALB/c mice. Benz was administered intraperitoneally (i.p.) at a dose of 0.5 mg/mouse thrice a week. PBS was injected as the vehicle control. Primary tumors and secondary tumors on the lungs were resected on day 22 of the post-injection period. (**B**) The images of the primary tumors. Scale bar, 5 mm. (**C**) The weights of the primary tumors. **(D**) Representative images of the lungs. Arrowheads indicate typical metastatic nodules. Scale bar, 5 mm. (**E**) The weights of the lungs. (**F**) The number of metastatic tumor nodules on the lungs. (**G**) The number of mice containing ten or more metastatic tumor nodules on each lung. Ctrl group: 7/7, Benz group: 2/7. (**H** to **L**) Evaluation of circulating tumor cells (CTCs). (**H**) Schedule of CTCs analysis after s.c. injection of LuM1/m9 and i.p. injection of Benz into BALB/c mice. LuM1/m9 (500k cells) was injected into the side abdominal wall of BALB/c mice. Benz was i.p. injected every other day. PBS was injected as a negative control. (**I**) Scatter plot analysis of CTCs. ZsGreen-positive CTCs were analyzed using On-chip Sort. Representative data were shown. (**J**) The number of CTCs of each group at one to four weeks after the injections. * *P* = 0.0062 (vs. PBS group), *P* = 0.0389 (vs. Tumor+Benz group). Mean + SD; PBS, n = 5; Tumor injected, n = 4; Tumor+Benz, n = 7. (**K**) The number of CTCs per 40 μL of mouse blood at the 3 weeks post-transplantation period.

To evaluate whether Benz could suppress the tumor growth and lung metastasis of aggressive cancer cells, we used a tumor allograft mouse model using the BALB/c-derived LuM1 injection into BALB/c mice. Intraperitoneal injection of Benz (at 0.5 mg/mouse, thrice weekly for three weeks) significantly suppressed tumor growth (Figure 6A–C) and attenuated metastasis to lungs (Figure 6D–G). Benz treatment reduced tumor weight, lung weight, and the number of metastatic tumor nodules on the lungs as compared to the control group.

CTCs are cells that have shed into the vasculature or lymphatics from a primary tumor and that are then carried around the body in the blood circulation [30]. We have established a cytometric method to evaluate fluorescent cells on a chip. Next, we asked whether subcutaneously injected LuM1/m9 ZsGreen-positive fluorescent tumor cells could enter into the blood circulation, being detectable by using the on-chip cytometric system. The gate of CTCs was determined by comparing tumor cells injected with PBS (3 mice) vs. with LuM1/m9 cells (3 mice) at 3 weeks after the injections and decided as areas with ZsGreen-positive cells and without cells in the PBS-treated group (Figure S11A–C).

We also asked whether intraperitoneally injected Benz could reduce LuM1/m9 tumor-derived CTCs in the tumor allograft mouse model. ZsGreen-positive CTCs were undetectable at 1 week after the injection, while ZsGreen-positive CTCs (10 to 2500 cells) were detectable in 40 μL of blood sample per individual mouse at 2 to 4 weeks after the injection of LuM1/m9 tumor cells (Figure 6H,I; Figure S11). The number of CTCs was highest at 3 weeks after the injection of tumor cells and decreased at 4 weeks post-injection (Figure 6I,J; Figure S11D). The intraperitoneal injection of Benz markedly reduced the number of CTCs derived from the subcutaneous LuM1/m9 tumors (Figure 6I–K; Figure S11D–F). Among the mice in the tumor-injected and tumor/Benz-injected groups, most mice showed a similar tendency in the number of CTCs at 3 weeks, while each mouse showed a much larger number of CTCs as compared to the other mice (Figure S11E,F). We expected possibilities of: (i) a technical error, (ii) rapid increase of CTCs in the mice over a week, or (iii) individual differences. When the data that were far apart were omitted, there were statistically significant differences between tumor-injected (n = 4) vs. PBS-treated (n = 5) groups and between tumor-injected (n = 4) vs. tumor+Benz-injected (n = 7) groups (Figure 6K).

Therefore, it is suggested that Benz treatment suppresses primary tumor growth, seeding of cancer cells into the circulation, and secondary tumorigenesis. 

### 2.7. Clinical Significance of DAT and STAT

**Figure 7:**
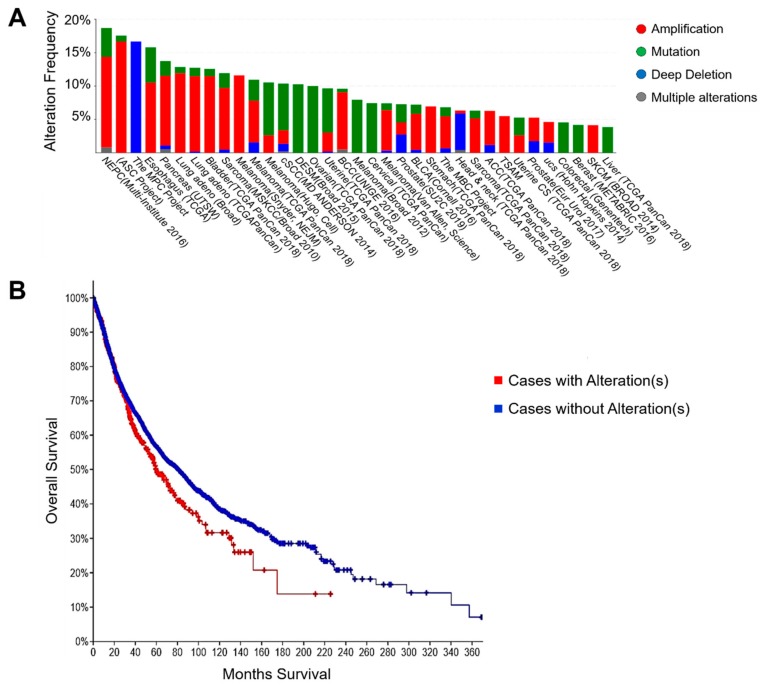
**The clinical significance of SLC6A3/DAT in cancer.** (**A**) Genetic alteration of DAT/SLC6A3 from a combined study (44,313 patients/46,641 samples). (**B**) Overall survival Kaplan-Meier estimate of cases with or without DAT/SLC6A3 alteration(s). Data were obtained from TCGA PanCancer Atlas (10,953 patients/10,967 samples).

Next, we searched the database to determine whether genetic alterations in the DAT/SLC6A3 gene could be clinically significant. The genetic amplification of DAT/SLC6A3 was frequently found in 14% of castration-resistant prostate cancer (CRPC, known as neuroendocrine tumor), 12% of pancreatic cancer, 13% of lung squamous cell carcinoma (SCC), 11% of lung adenocarcinoma, 12% of esophagus cancer, 12% of sarcoma, 9% of bladder cancer, and 8% of ovarian cancer (Figure 7A, Figure S7). A high expression of DAT/SLC6A3 mRNA was found in renal cell carcinoma, lung adenocarcinoma, prostate cancer, lung SCC, pancreatic cancer, ovarian cancer, stomach cancer, esophagus cancer, sarcoma, and head and neck cancer (Figure S8). 

Cancer cases with DAT/SLC6A3 alteration(s) were significantly correlated with a poorer prognosis of cancer patients as compared to the no alteration group, as shown by the overall survival Kaplan-Meier estimate (Figure 7B, Table 1, Figure S9). These data indicate that the DAT/SLC6A3 is often genetically amplified in many types of cancers and involved in poor prognosis and that it is therefore sensibly targetable by DAT inhibitors such as Benz. It has been shown that STAT3 was able to activate the autocrine feedforward loop by inducing oncostatin M, oncostatin M receptor (OSMR), IL-31, IL-31 receptor, and gp130 [glycoprotein 130, also called IL-6 signal transducer (IL6ST), IL6Rβ, oncostatin M receptor subunit α] [31]. Next, we therefore searched to determine whether the expression of STAT3 and DAT/SLC6A3 were correlated with the expression of other genes related to the STAT and NF-κB signals in colorectal adenocarcinoma patient-derived samples (594 cases). We found a negative correlation between DAT vs. BCL10, an inducer of apoptosis through the recruitment of caspases, suggesting that DAT expression could be involved in a potent anti-apoptotic activity (Table S1). DAT/SLC6A3 expression was positively correlated with STAT5B and CTNNBL1 encoding β-catenin (Spearman’s rank correlation coefficient: 0.29 to 0.35).

The STAT3 expression was significantly correlated with the gene expression of JAK-STAT and NF-κB signaling factors, including JAK1, gp130, STAT5A, REL, NFKB1, OSMR, and IL6R (Spearman’s rank correlation coefficient: 0.40 to 0.57) (Table S1, Figure S10). However, lower correlations of STAT3 expression were found with IL6, OSM, IL31RA, and IL31 (Spearman’s rank correlation coefficient: 0.005 to 0.24) (Table S1).

Therefore, the STAT/gp130/OSMR/JAK feedforward loop and NF-κB (REL, NFKB1) may operate combinatorially in cancer. Benz can block such a STAT/NF-κB combination, as the data above has shown.

## 3. Discussion

**Figure 8:**
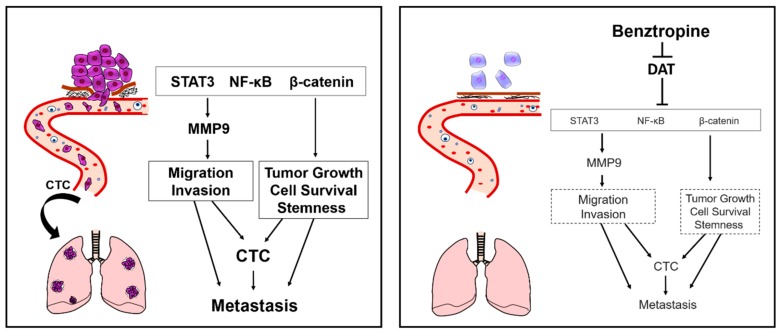
**Graphical abstract**. Tumor growth, enhanced migration, invasion, and stemness are essential for the properties of aggressive, refractory cancers. STAT3, NF-κB, and β-catenin are oncogenic signaling trans-activators crucial for these properties and for activation of the MMP9 gene. These phenotypes often enable tumor cells to disseminate to the bloodstream as CTCs and to distant organs for establishing metastasis. Benztropine suppresses the properties of aggressive, refractory cancers by acting on the dopamine transporter SLC6A3 and reducing the pro-tumorigenic factors including STAT3, NF-κB, and β-catenin.

Our study demonstrates the anti-tumor effect of Benz on suppressing tumor growth, CTCs, and metastasis (Figure 8: graphical abstract). Our findings strongly suggest repositioning the drug benztropine for it to become anticancer therapeutic, particularly in relation to targeting refractory, metastatic, and lung SCC, lung adenocarcinoma, melanoma, sarcoma, and other cancers. Furthermore, the recurrent cancers with the frequent genetic amplification of SLC6A3, such as pancreatic cancer, and CRPC, indicate the potential cross-fertilization between two research fields when carrying out the re-drug positioning analysis; the properties of Benz in the treatment of Parkinson’s disease cast significant light on its potential mechanisms in cancer therapy. 

We revealed the tremendous antitumor effects of Benz using the in vitro 3D tumoroid model, being phenotypically closer to in vivo tumors than 2D cultured cells. A number of studies, including ours, have shown that MMP9 at the levels of gene expression and proteolytic activity are established markers of aggressive tumors with potent activities of migration, invasion, angiogenesis, and metastasis [5]. Benz markedly inhibited MMP9 expression concomitant with tumor cell migration and invasion and suppressed the dissemination of the tumor cells to the blood circulation and to metastatic secondary tumors in the lungs. These data are consistent with our previous report that the knockdown of MMP9 and MMP3 inhibited the tumorigenesis and metastasis of aggressive colon cancer cells [4]. Besides, our present study demonstrated that Benz, a single agent, reduced pro-tumorigenic signaling/transcriptional components, including STAT, NF-κB, and β-catenin, as well as MMP. A recent study reported that breast cancer stem cells were inhibited by benztropine mesylate in vitro by using stem cell markers such as aldehyde dehydrogenase activity, CD44+/CD24- phenotype, and the number of spheroids [32]. However, recent studies revealed that CD326/EpCAM and CD44 variants are definitive markers of CSC/CIC properties rather than the CD44 standard [16,33,34]. Our study revealed that Benz reduced STAT3, NF-κB, β-catenin, and CD326/EpCAM, which are cancer cell survival factors, as well as CSC/CIC markers, concomitant with the inhibition of a number of pro-tumorigenic malignancy events. Another recent study reported the preventive action of Benz on platinum-induced peripheral neuropathies and tumor growth [35]. Their data indicated that Benz alone did not inhibit tumor growth, although a synergistic effect between oxaliplatin and Benz could be found. A synergistic effect of Benz and paclitaxel was also reported for inhibiting tumorspheres of breast cancer stem cells [32]. Nevertheless, our study indicates that targeting SLC6A3/DAT can be useful for cancer research and therapeutics, while the combination with other therapeutics can withdraw synergistic effects with lower doses and less toxicity as much as an independent MoA between the DAT inhibitor and other anticancer medications.

Our study also indicates that the frequent, genetic amplification of SLC6A3/DAT is targetable in cancers. In accordance with this, SLC6A3 was recently identified as a biomarker for patients suffering from renal cell carcinoma [36]. Thus, amplified, overexpressed SLC6A3/DAT could be a novel promising target in cancer research and treatment. Our study pharmacologically indicates that Benz, GBR-12935 (a selective DAT inhibitor), and Amitriptyline (a non-selective MAT inhibitor) targeted SLC6A3/DAT, inasmuch as these drugs significantly suppressed tumorigenesis and cancer cell viability, whereas neither muscarinic antagonists, the NET inhibitor nisoxetine, the dopamine receptor antagonist sulpiride, nor dopamine did. Thus, Benz could directly target SLC6A3/DAT on tumor cells independently of the dopamine uptake. Dose-response relationships that are different among GBR-12935, Benz, and Amitriptyline may result from their different selectivity, binding affinities to target molecules, or MoA in the cells. It has been known that GBR-12935 selectively binds to DAT at a very low concentration (K_D_ = 5.5 nM in rat striatal membranes) [37], while Benz requires much higher concentrations for binding to DAT (K_i_ = 130 to 237 nM) [25]. Amitriptyline also requires a much higher concentration for binding to DAT (K_i_ = 3 μM) than Benz or GBR-12935 do [38]. In the first place, it has been shown that Benz rather antagonized the muscarinic M1 receptor at a very low concentration (K_i_ = 0.59 nM in rat brain membranes), while inhibiting DAT as well as the serotonin transporter does at a much higher concentration (K_i_ = 5 μM) and acid sphingomyelinase by 87% at 10 mM [26,39]. Nevertheless, repurposing Benz for cancer treatment might not require pharmacodynamics, pharmacokinetics, ADME, and toxicology testing. A successful example is Metformin, an antidiabetic medication also effective for cancer prevention and treatment [40]. Our data also indicate that Benz effectively destroyed preformed tumors and also prevented in vitro tumorigenesis. However, repurposing Benz at a high dose might cause anticholinergic, antimuscarinic (side) effects. Otherwise, other DAT-binding agents are a considerable resource of drug repositioning and development for the anticancer purpose, including GBR-12935, other selective DRIs, or DAT-imaging agents. For example, Ioflupane (^123^I) (DaTSCAN®; GE Healthcare) has a high binding affinity for presynaptic DAT in the brains of mammals and is currently used for the detection of dopaminergic neuron degeneration in the differential diagnosis of Parkinson’s disease [41]. Currently, available DAT-binding agents are purposed to penetrate the blood-brain barrier (BBB) to reach dopaminergic neurons in brains. However, repurposing DAT-binding agents at a high dose can cause dopaminergic (side) effects such as adverse neurotoxicity as well as substance addiction by stimulating the reward system. Therefore, repurposing DAT-binding agents for cancer treatment could require the chemical modification of the lead compounds into a BBB-non-penetrating structure avoiding adverse effects in brains. The side effects may also involve alternative splicing variants of DAT and NET expressed in human blood cells and placenta, respectively [42]. Nevertheless, our study indicates that amplified SLC6A3/DAT in tumor cells is a promising novel target in cancer research and treatment. 

We pursued a molecular MoA for Benz in its inhibition of tumor progression. Our data indicated that STAT is a key molecule mediating the antitumor effect of Benz. STAT3 was most significantly suppressed by Benz at the mRNA level, at the phosphorylation level, and in the unphosphorylated short-form expression in the tumoroids. STAT3 is a member of the STAT family essential for anti-apoptotic cell survival and proliferation (by inducing c-Myc, Bcl2, Cxcl12/SDF1, Ccnd1, Cxcr4, and by mediating the mitochondrial oxidative stress response), migration and invasion (by inducing MMPs), oncogenic transformation (by mediating Src signal), stemness (by inducing IL-6 and Cxcl3), and EMT (by inducing twist and vimentin) [43]. A co-expression correlation analysis revealed that STAT/gp130/OSMR/JAK and NF-κB signaling factors are simultaneously expressed in colon cancer. These data are consistent with a recent report that STAT3/oncostatin signaling is essential for EMT-driven cancer stemness in triple-negative breast cancer [44]. Notably, STAT3 is an auto-activator for *STAT3* gene transcription, and the STAT3/NF-κB heterodimer can bind to NF-κB binding sites in gene promoters [28]. Our data indicated that NF-κB/RelA was simultaneously reduced along with the complete loss of STAT3 by Benz treatment. Therefore, NF-κB might be destabilized and degraded following the loss of the partner STAT3. In accordance with this, a recent study showed that napabucasin, a STAT3 inhibitor, suppressed the proliferation, invasion, and stemness of glioblastoma cells [45]. In this study, napabucasin also disrupted the NF-κB signaling pathway via the downregulation of RelA, suggesting a similar MoA with Benz. Nevertheless, we independently demonstrated that STAT3 signaling, a common target in cancers, was efficiently targetable by Benz.

Our study is original in touching upon a 50-kD fragment of uSTAT3 found only in the cell nuclei of 3D tumoroids. It has been known that the major isoforms of STAT3 are STAT3α (86 kD) and STAT3β (79 kD), while two papers reported that a 50-kD STAT3 was generated by caspase 3-dependent cleavage [46]. In the 3D tumoroid model, we previously observed that tumoroids contain cellular heterogeneity, similarly to tumor heterogeneity in vivo [17]. We have also observed active caspase 3-positive cells in the tumoroid heterogeneity. Otherwise, intracellular MMPs could cleave intranuclear proteins such as STAT in accordance with what we have proposed [4,7]. Indeed, it has been shown that, upon stress conditions, inducible and activated MMP3 in dopaminergic cells digested α-synuclein in dopaminergic neurons, playing a pivotal role in the progression of Parkinson’s disease through the modulation in the aggregation of C-terminal truncated α-synuclein, Lewy body formation, and neurotoxicity [47]. The 50-kD uSTAT3 was detectable by the anti-N-terminus STAT3 antibody, but not by the anti-C-terminus phosphorylated STAT3 antibody that recognizes phosphorylation sites at the C-terminus. Thus, the 50-kD STAT3 does not contain established phosphorylation sites. However, it has been shown that uSTAT3 is a functional transactivator and that it is able to form a transcriptionally active uNF-κB/uSTAT3 heterodimer to induce NF-κB target genes [28]. As a single agent, Benz was able to reduce the levels of the p-STAT3, uSTAT3, and NF-κB, factors essential for cancer cell survival and tumor progression. 

Our data also touch upon another pathway by which β-catenin could mediate the anti-tumor effect of Benz. Previous studies, including ours, have shown that the Wnt/β-catenin/MMPs axis was targetable in the CSC/CIC phenotype by ART [2,48]. Indeed, this repurposing drug, ART, was generally well tolerated in clinical studies on colorectal cancer [49]. The Wnt/β-catenin/MMPs regulatory axis is essential to CSC/CIC properties, which are known to enhance metastasis and recurrence. It has been shown that the CSC marker CD326/EpCAM could form a protein complex with β-catenin [29]. Our current data indicate that CD326 and β-catenin at both the protein and mRNA levels were simultaneously lowered by Benz. The reduction of β-catenin might also destabilize CD326. On the other hand, pluripotency transcription factors were not reduced at the mRNA level by Benz treatment, suggesting that Benz may not inhibit the adult stem cells required for tissue repair and regeneration. Nevertheless, our data definitively show that Benz stifles CSC/CIC properties by reducing β-catenin, CD326, and STAT3 simultaneously.

## 4. Materials and Methods

### 4.1. Cell Line and Cell Culture

A rapidly metastatic colon cancer cell line LuM1 was established from the mouse colon cancer cell line Colon26 [4,50]. LuM1/m9 reporter cells were established by the stable transfection of a murine *Mmp9* promoter (588 bp)-driven ZsGreen fluorescent reporter construct into LuM1 cells [2]. HCT116, a human colorectal cancer cell line, was obtained from ATCC. LuM1, LuM1/m9, and HCT116 were cultured in RPMI1640 with 10% FBS supplemented with penicillin, streptomycin, and amphotericin B for 2D culture, or in mTeSR1 stem-cell medium (Stemcell Technologies, Vancouver, Canada) for 3D culture, as described [16]. NanoCulture Plate (NCP) (Medical & Biological Laboratories, Nagoya, Japan) or ultra-low attachment (ULA) culture plates/dishes (Greiner, Kremusmunster, Austria) were used for the 3D culture, as described [17]. 

### 4.2. Three-Dimensional Tumoroid-Based Multiplex Reporter Assay

A tumoroid-based multiplex reporter assay was carried out as described previously [2]. We used two experimental systems to evaluate the effects of drugs on (I) new tumoroid formation (Figure 1A) and on (II) pre-formed tumoroids (Figure 1F). For system I, cells were seeded at 5 × 10^3^ cells/well in a 96-well NCP and cultured in mTeSR1 for 72 h with or without drugs. For system II, cells were seeded at 5 × 10^3^ cells/well in a 96-well NCP and then pre-cultured in mTeSR1 for 48 h to form tumoroids without drugs. The drugs were then administrated to the pre-formed tumoroids. For the measurement of the fluorescent intensity and area (μm^2^ = pixel), each tumoroid per well was calculated using the ArrayScan™ high contents screening (HCS) system (ThermoFisher, Waltham, MA). Fluorescent areas greater than 300 μm^2^ were counted as tumoroids. The *Mmp9* promoter activity was evaluated by an average fluorescence intensity per μm^2^ of all cells in a well. The experiments were performed with three or four biological replicates.

### 4.3. Chemicals and Drugs

Artesunate, benztropine mesylate, carboplatin, and 9α, 15R-dihydroxy-11β-fluoro-15-(2,3-dihydro-1H-inden-2-yl)-16, 17, 18, 19, 20-pentanor-prosta-5Z, 13E-dien-1-oic acid (AL8100) were purchased from Cayman Chemical (Ann Arbor, MI). Amitriptyline, GBR-12935, and nisoxetine were purchased from Sigma-Aldrich (St Louis, MO). Chlorzoxazone and 2-Amino-5-phosphonopentanoic acid (AP5) were purchased from Toronto Research Chemicals (Toronto, Canada). Atropine (Nacalai Tesque, Kyoto, Japan), sulpiride (Tokyo Chemical Industry, Tokyo, Japan), trihexyphenidyl (THP) (Wako, Osaka, Japan). All drugs were prepared as stock solutions with a concentration of 10 mM in ethanol. 

### 4.4. Cell Viability Assay

The ATP content was quantified using a CTG Luminescent Cell Viability Assay (Promega, Madison, WI). Briefly, from the total 200 μL of culture supplement, 150 μL was removed, and 50 μL of CTG solution was added to each well and then suspended. The plate was rocked for 2 min and incubated for 10 min at 37 °C. The luminescence was measured in a plate reader (Molecular Devices, San Jose, CA). From the cytotoxicity data, IC_50_ was calculated using a GraphPad Prism (La Jolla, CA).

### 4.5. Wound Healing Assay

LuM1/m9 cells (1 × 10^5^ cells) were seeded in 24-well plates and pre-cultured for 24 h. Cells were treated with 50 μM Benz for 24 h, and then the medium was replaced with a fresh one. Cells were wounded by scratching with pipette tips. Images were captured immediately and at 24 h using the ArrayScan™ HCS system. The percentage of the area closed in 24 h was measured using Image J (https://imagej.nih.gov/ij/).

### 4.6. Lactate Dehydrogenase Release Assay

The cytotoxicity was measured using the index of lactate dehydrogenase (LDH) release from cells and expressed as a percentage of the total cellular activity. LuM1 cells were seeded at 5000 cells/well in 96-well NCP and cultured with or without Benz. The culture medium was transferred to the other 96-well plates at 24 h, 48 h, and 72 h after Benz addition. The LDH activity was measured using the LDH cytotoxicity assay kit according to the manufacturer’s instructions (Nacalai Tesque, Kyoto, Japan) by measuring the absorbance (490 nm).

### 4.7. Migration/Invasion Assays

In vitro migration/invasion assays were performed using uncoated and Matrigel-coated transwell assays (Becton Dickinson, Franklin Lakes, NJ), respectively, as described [4]. LuM1 cells were pre-treated with Benz at 50 μM for 24 h, and then 5 × 10^4^ cells were re-seeded into the upper chambers of a Transwell® 24-well (Corning, NY). The migrating or invading cells on the lower surfaces of the filters were fixed at the 24 h post-cell-transfer period and stained using Diff-Quick (Sysmex, Hyogo, Japan). The number of migrating/invading cells in the five fields (5.3 mm^2^/filed) was counted using Image J.

### 4.8. Promoter Analysis

A 588-bp cDNA fragment of the *Mmp9* promoter region between –569 and +19 was cloned via genomic PCR from a tail of a BALB/c mouse and sequenced, as described [2]. Human and murine *Mmp9* promoter sequences were also obtained from the Eukaryotic Promoter Database. Transcription factor binding sites were predicted using PROMO (ver. 8.3), as described [51]. Sequences were aligned using NCBI BLAST. STAT binding sequences were analyzed according to consensus sequences [52,53].

### 4.9. Protein Sample Fractionation

For the detection of MMP9, cells were pre-treated with Benz at 50 μM for 24 h and then cultured in a serum-free medium for 24 h. Cell culture supernatants were concentrated eight times using Amicon Filtration tubes for MW. 10,000. LuM1 cells were washed with PBS, collected using a cell scraper, and lysed in RIPA buffer containing a protease inhibitor cocktail (Sigma-Aldrich, St Louis, MO). To investigate other proteins, cells were seeded at 1.6 × 10^5^ cells/well in a 6-well ULA plate and cultured in mTeSR1 for 72 h with or without 20 μM Benz. The nuclear/cytoplasmic subcellular fractions were prepared using a NE-PER nuclear and cytoplasmic extraction kit (ThermoFisher), according to the manufacturer’s instruction. 

### 4.10. Western Blot Analysis

The protein samples were loaded onto 8% or 10% polyacrylamide gel and transferred to a PVDF membrane by using the semi-dry method. Blocking and antibody reactions were done in blocking buffer containing 5% skim milk (Wako, Osaka, Japan) in Tris-buffered saline containing 0.05% Tween 20 (TBS-T). An anti-MMP9 (Abcam, Cambridge, UK), anti-β-catenin [Cell Signaling Technology (CST), Danvers, MA], anti-phosphorylated STAT3 (CST), anti-NF-κB p65 (CST), anti-STAT3 (Proteintech, Chicago, IL), anti-EpCAM (Abcam), anti-Histone H3 (CST), and HRP-conjugated anti-GAPDH antibody (Wako) were used. The quantitative densitometric analysis was performed using Image J.

### 4.11. RT-qPCR

RT-qPCR was performed as described [2]. Briefly, LuM1 cells were seeded at 1.6 × 10^5^ cells/well in a 6-well ULA plate and cultured in mTeSR1 for 24 h or 48 h with or without 20 μM Benz. The total RNA was extracted using the AGPC method with Trizol (Molecular Research Center, Cincinnati, OH). cDNA was synthesized using ReverTra Ace (Toyobo, Osaka, Japan). A real-time PCR was carried out by using iQ cyber (BioRad). Primers for *Mmp9, Epcam, Stat3, Ctnnb1*, and *Hprt1* (an internal control) [17] were listed in Table S2. The relative mRNA levels to *Hprt1* mRNA levels were quantified by the ΔΔCt method using the following formula: fold change = 2^−ΔΔCt^. 

### 4.12. Genetic Alterations, Gene Expression, Kaplan-Meier Estimate

A TCGA PanCancer Atlas combined study (10,953 patients/10,967 samples) and curated set of non-redundant studies (44,313 patients/46,641 samples) were analyzed to examine the genetic alterations, RNA-seq-based mRNA expression levels, and Kaplan-Meier estimate, by using cBioPortal [54,55].

### 4.13. Coexpression Correlation Analysis

The co-expression correlations between two genes were analyzed among the colorectal adenocarcinoma cases of 594 patients/594 samples (TCGA, PanCancer Atlas) by using cBioPortal.

### 4.14. Tumor Allograft and CTC

All animal experiments were performed according to the guidelines for the care and use of laboratory animals approved by Okayama University and the Japanese Pharmacological Society (OKU-2015659). LuM1 cells or LuM1/m9 ZsGreen reporter cells (5 × 10^5^ cells) suspended in 50 μL PBS were transplanted subcutaneously at the side abdominal wall of 6-week-old female BALB/c mice. As a negative control, 50 μL PBS was injected. The mice were intraperitoneally injected with Benz (500 μg/mice) thrice a week for three to four consecutive weeks. 

For the analysis of CTCs, blood (40 μL) was collected in the tubes containing EDTA solution from each mice tail vein at one, two, three, and four weeks after the injection of LuM1/m9 ZsGreen cells. The blood samples were treated with RBC Lysis Buffer (BioLegend; 1 ml per sample) for 15 min at RT, and then centrifuged 400 × *g* for 15 min at 4 ℃. The supernatant was removed, and then the remaining pellet was suspended with 100 μL On-chip T buffer (On-chip Biotechnologies, Tokyo, Japan). The suspension was filtered with a CellTrics 150 µm cell strainer (Sysmex, Kobe, Japan) and then analyzed using an On-chip Sort LS5 (On-chip Biotechnologies). The gate to define CTCs was decided as the area that contained ZsGreen-positive cells and without PBS-treated mice-derived cells, as shown in Figure S11A,B and Figure 6I.

Twenty-two days after the transplantation of LuM1 cells, the subcutaneous tumors and lungs were resected, and then fixed with 4% formalin or Bouin’s fluid. The number of nodules with a diameter above 0.5 mm was counted.

### 4.15. Statistics

The data were expressed as the means ± SD unless otherwise specified. The statistical significance was calculated using GraphPad Prism (La Jolla, CA). Three or more mean values were compared using a one-way analysis of variance with the pairwise comparison via Dunnett’s method, while comparisons of two were made with an unpaired Student’s *t*-test. *P* < 0.05 was considered to indicate statistical significance.

## 5. Conclusions

In conclusion, therefore, we propose the drug repurposing of Benztropine and DAT-binding agents for anticancer research and therapeutics that can suppress tumor progression, circulating tumor cells, and metastasis by acting on SLC6A3/DAT and by reducing key pro-tumorigenic factors, including STAT, NF-κB, β-catenin, CD326, and MMP9.

## Figures and Tables

**Table 1:** Correlation of DAT/SLC6A3 genetic alterations with a poor prognosis of cancer patients.

Data Set	TCGA PanCancer Atlas	A Curated Set of Non-Redundant Studies
Number of patients	10,953 patients	44,313 patients
Number of samples	10,967 samples	46,641 samples
Overall survival, P-value	0.0268	0.136
Disease/Progression-free survival, *P*-value	0.382	0.792

The data were expressed as a log-rank test *P*-value. Kaplan-Meier plots are shown in Figure 7 and Figure S9.

## Supplementary Materials

The following are available online at https://www.mdpi.com/2072-6694/12/2/523/s1. Figure S1. Cell viability and LDH release of tumoroids upon Benz treatment; Figure S2. Western blotting showing MMP9 and GAPDH supporting Figure 3D; Figure S3. Promoter analysis of mouse and human MMP9; Figure S4. Western blotting showing p-STAT3, STAT-3, NF-κB, β-catenin, Histon H3, and GAPDH supporting Figure 4C; Figure S5. Tumoroid progression and Benz treatment altered expression levels of Cd326, Hif1a, Nanog, Sox2, and Oct4; Figure S6. The size of tumoroids and MMP9 promoter activities altered by treatment with dopamine, a dopamine receptor antagonist (sulpiride), GBR12935, and their combinations; Figure S7. Alteration frequencies of the DAT/SLC6A3 gene among cancers; Figure S8. SLC6A3/DAT mRNA expression among cancers; Figure S9. Overall survival Kaplan-Meier estimate of cases with or without DAT/SLC63A alteration(s); Figure S10. Scatter plot analysis of the co-expression correlation of STAT3 with NF-κB and oncostating signaling; Figure S11. On-chip CTC analysis; Table S1. Expression correlation in colorectal adenocarcinoma patients (594 cases); Table S2. List of primers for RT-qPCR.
