# Supplementary material for: Antiparkinson Drug Benztropine Suppresses Tumor Growth, Circulating Tumor Cells, and Metastasis by Acting on SLC6A3/DAT and Reducing STAT3
Source: Cancers (Basel). 2020 Feb 24;12(2):523. doi: 10.3390/cancers12020523 (PMC7072357; doi:10.3390/cancers12020523)
Supplement: Supplementary file 1 [file cancers-12-00523-s001.zip › Benz supple proof.docx]

Supplementary Materials: Antiparkinson Drug Benztropine Suppresses Tumor Growth, Circulating Tumor Cells, and Metastasis by Acting on SLC6A3 and Reducing STAT3

Chiharu Sogawa^1^, Takanori Eguchi^1,8,^*, Manh Tien Tran^1^, Masayuki Ishige^2^, Kilian Trin^1,3^, Yuka Okusha^1,4^, Eman Ahmed Taha^1,5^, Yanyin Lu^1^, Hotaka Kawai^6^, Norio Sogawa^7^, Masaharu Takigawa^8^, Stuart K. Calderwood^4^, Kuniaki Okamoto^1^ , Ken-ichi Kozaki^1^


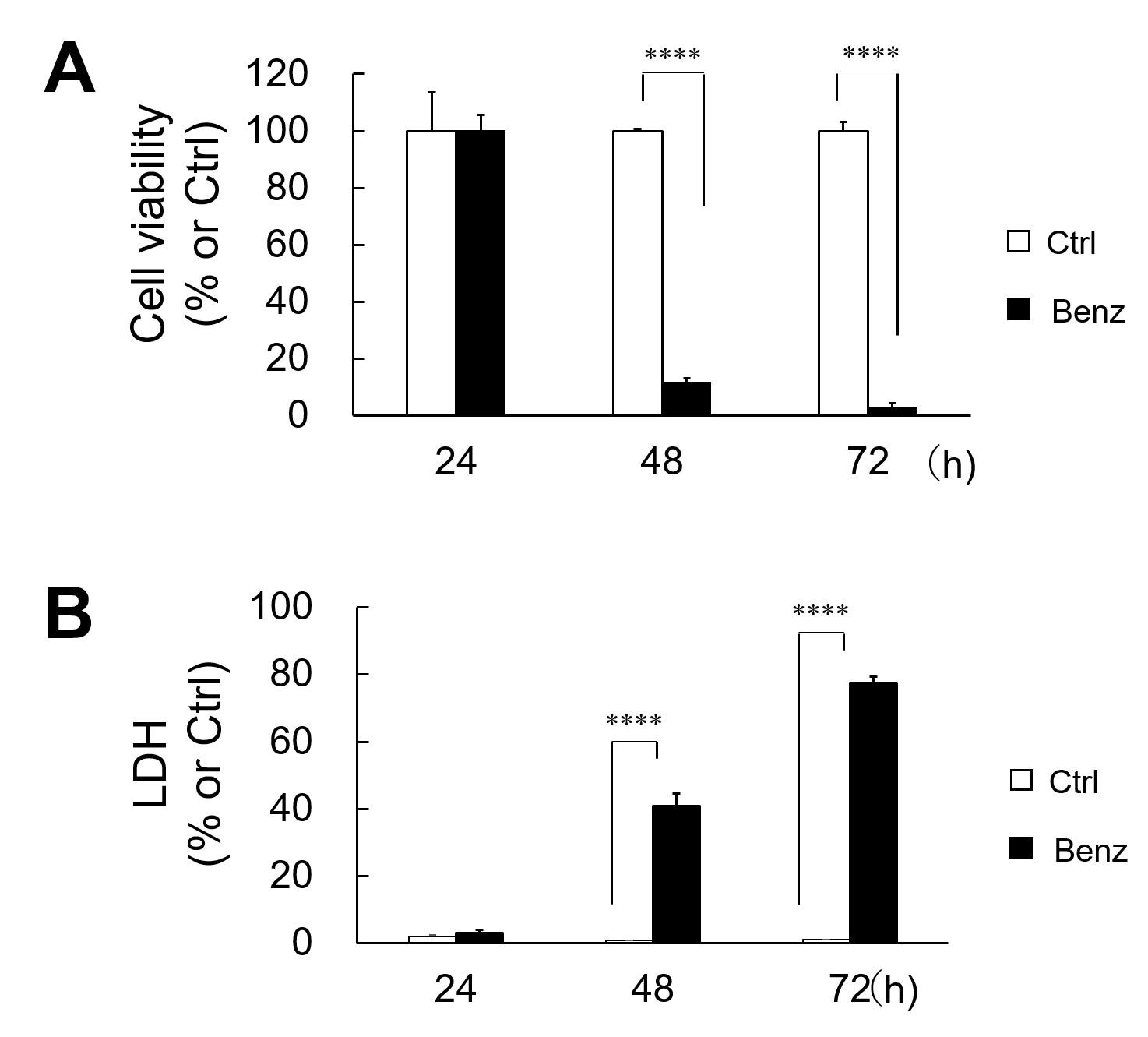


**Figure S1. Cell viability and LDH release of tumoroids upon Benz treatment.** LuM1 cells were treated with 20 μM benztropine (Benz) for 24 h, 48 h, and 72 h under 3D culture condition. A, cell viability; B, LDH release. Mean ± SD, n=3. ****P<0.0001 vs control (Ctrl) for each time point.


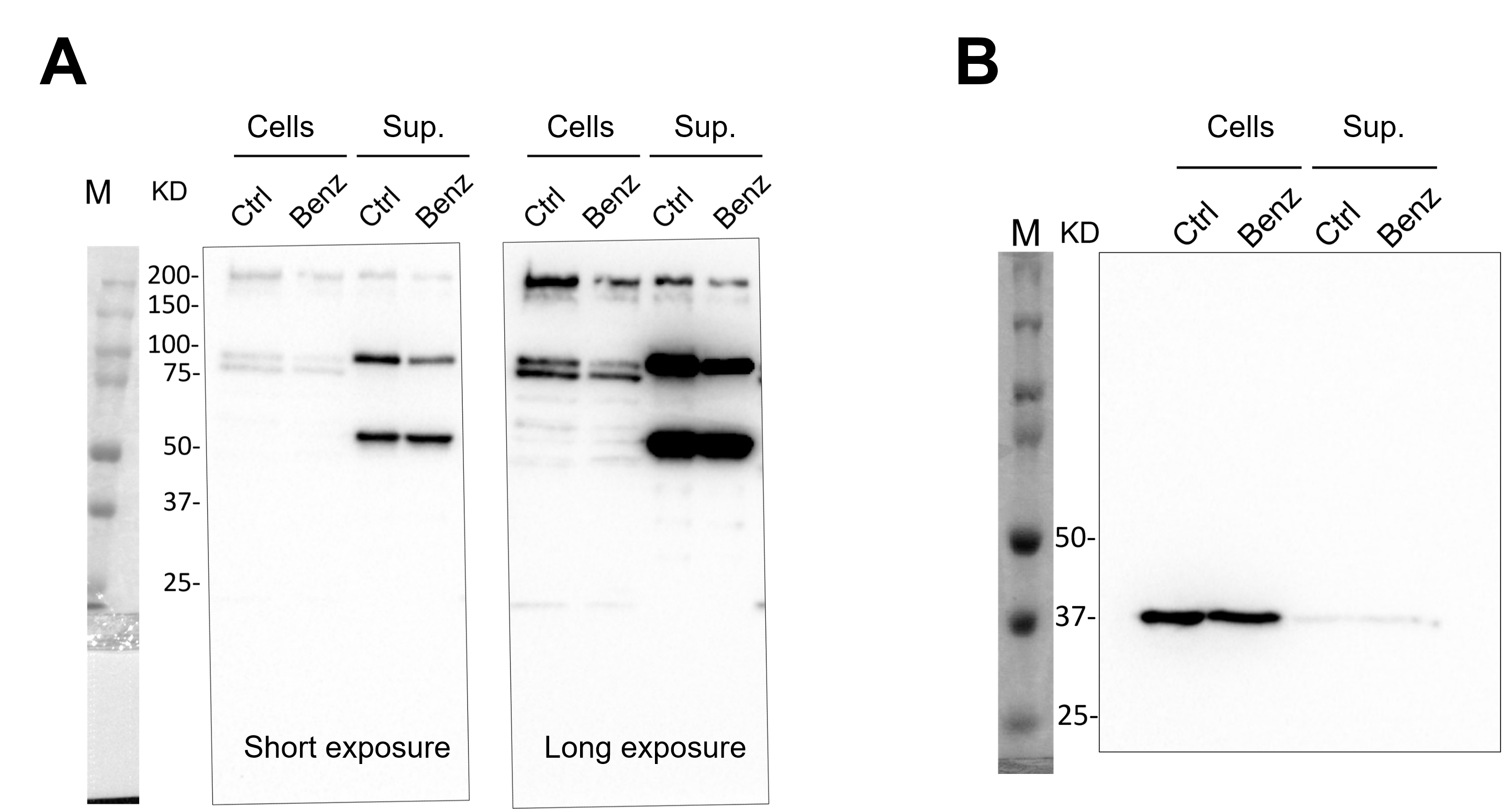


**Figure S2. Western blotting showing MMP9 and GAPDH supporting Fig. 3D.** Full images of the western blot analysis for MMP9 (A) and GAPDH (B). M, molecular weight marker. Cells, whole cell lysate. Sup., cell culture supernatant


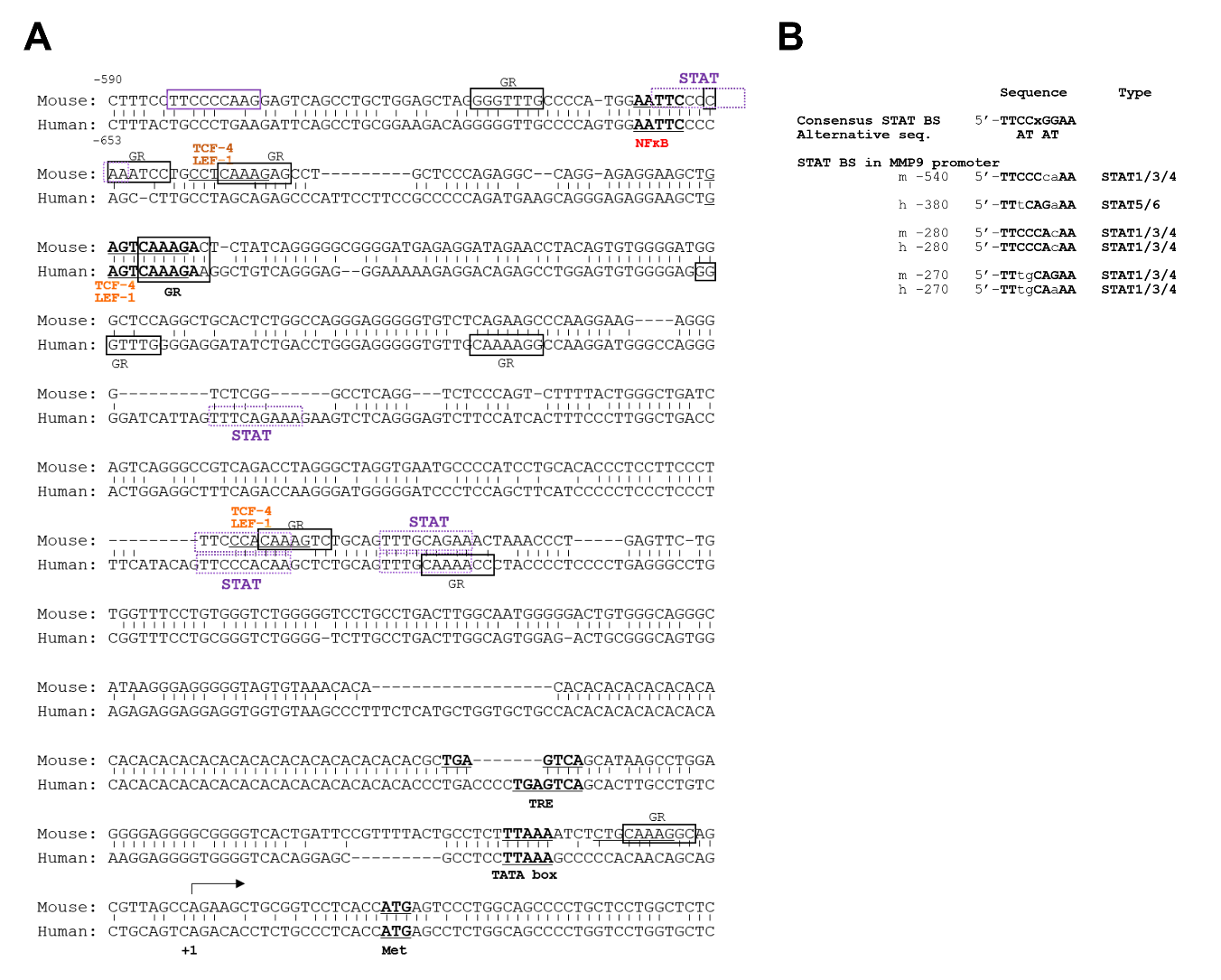


**Figure S3. Promoter analysis of mouse and human MMP9.** (A) Alignment of the MMP9 promoter sequences in human and mouse. Binding sites for STAT (purple), NF-κB (red), TCF-4/LEF-1 (orange), and GR (black) were mapped. (B) STAT binding sequences (BS) in the human and mouse MMP9 promoter regions. The nucleotide in the center defines preference of the types of STAT which bind to the sequence [75, 76]. x=C or G, STAT1/3/4 BS. x=A or T, STAT5/6 binding. Top, a consensus STAT BS and alternative sequence. Bottom, STAT BS and types of STAT that preferentially bind to the sequence.


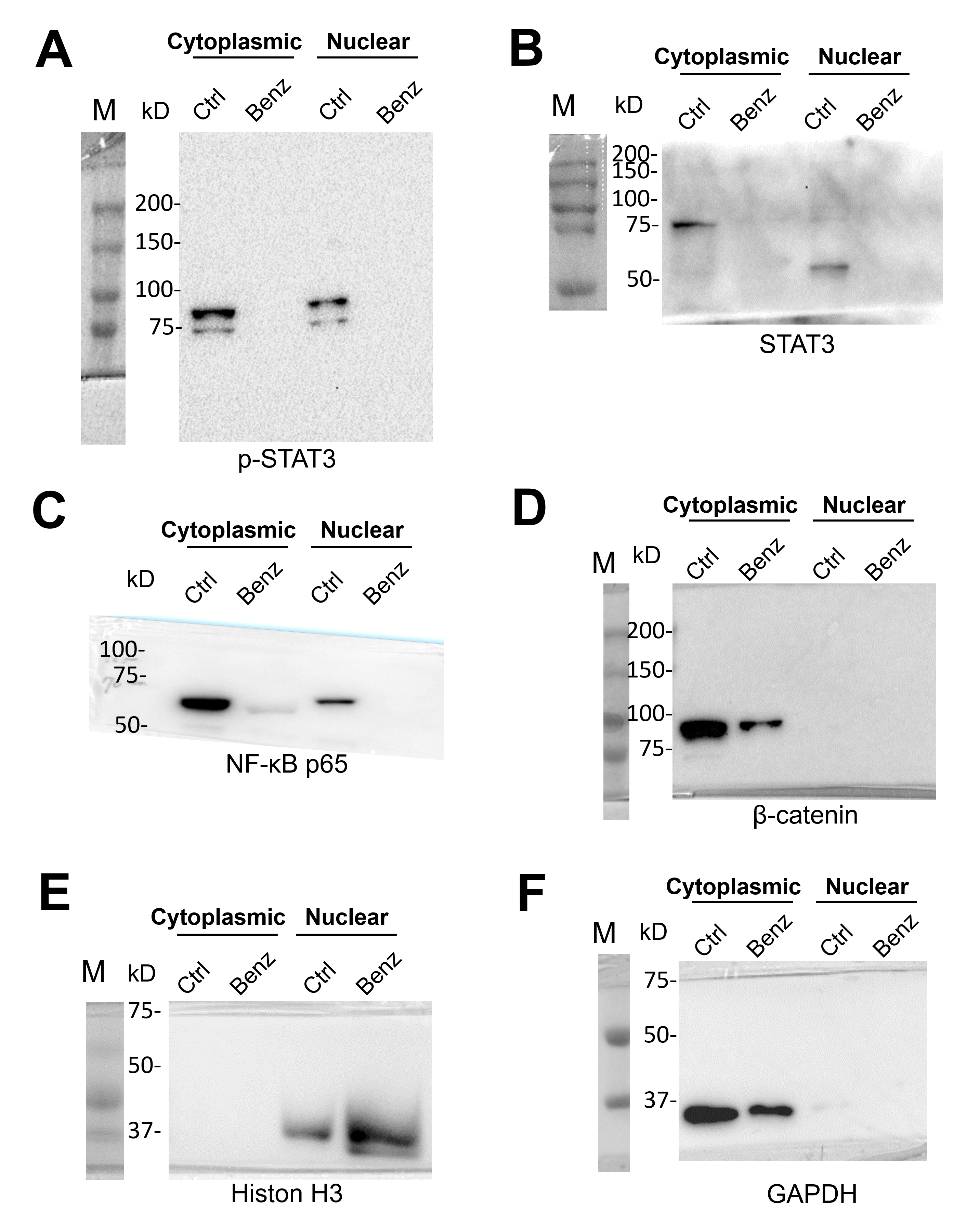


**Figure S4. Western blotting showing p-STAT3, STAT-3, NF-κB, β-catenin, Histon H3, and GAPDH supporting Fig. 4C.** Full images of the western blot analysis for p-STAT3 (A), STAT-3 (B), NF-κB (C), β-catenin (D), Histon H3 (E), and GAPDH (F). M, molecular weight marker.

**Figure S5. Tumoroid progression and Benz treatment altered expression levels of Cd326, Hif1a, Nanog, Sox2, and Oct4.** (A) Western blot showing CD326 in cytoplasmic fractions of LuM1 tumoroids. Tumoroids were treated with 20 μM Benz for 72 h. GAPDH, a cytoplasmic marker. (B) RT-qPCR analysis for mRNA levels of Cd326, Hif1a, Nanog, Sox2, and Oct4 altered by Benz. LuM1 cells were treated with 20 μM Benz for 24 h and 48 h under 3D culture condition. Mean ± SD, n=3. **P<0.01, ***P<0.001 and ****P<0.0001 vs Ctrl. mRNA levels were normalized with Hprt1 mRNA.


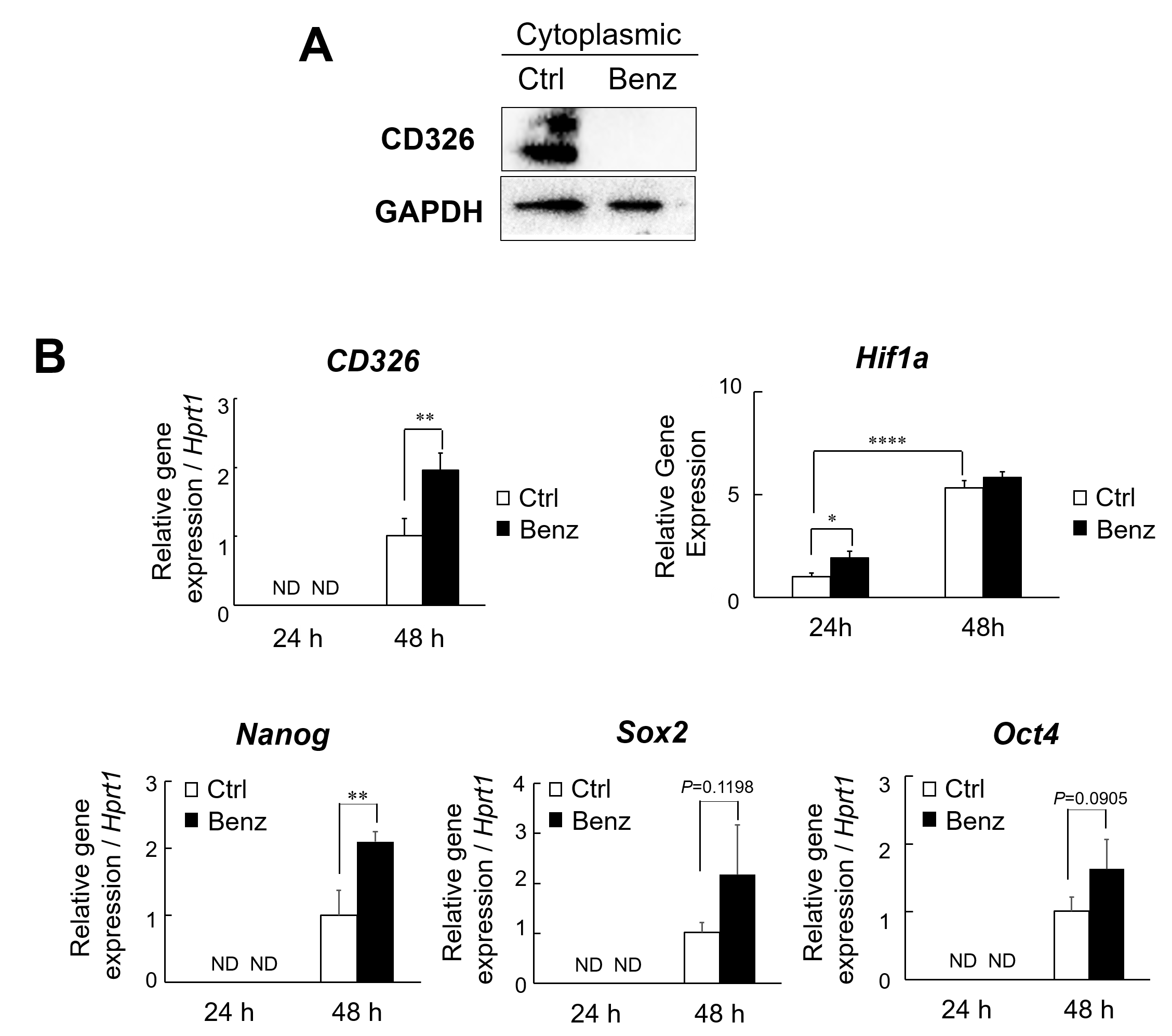


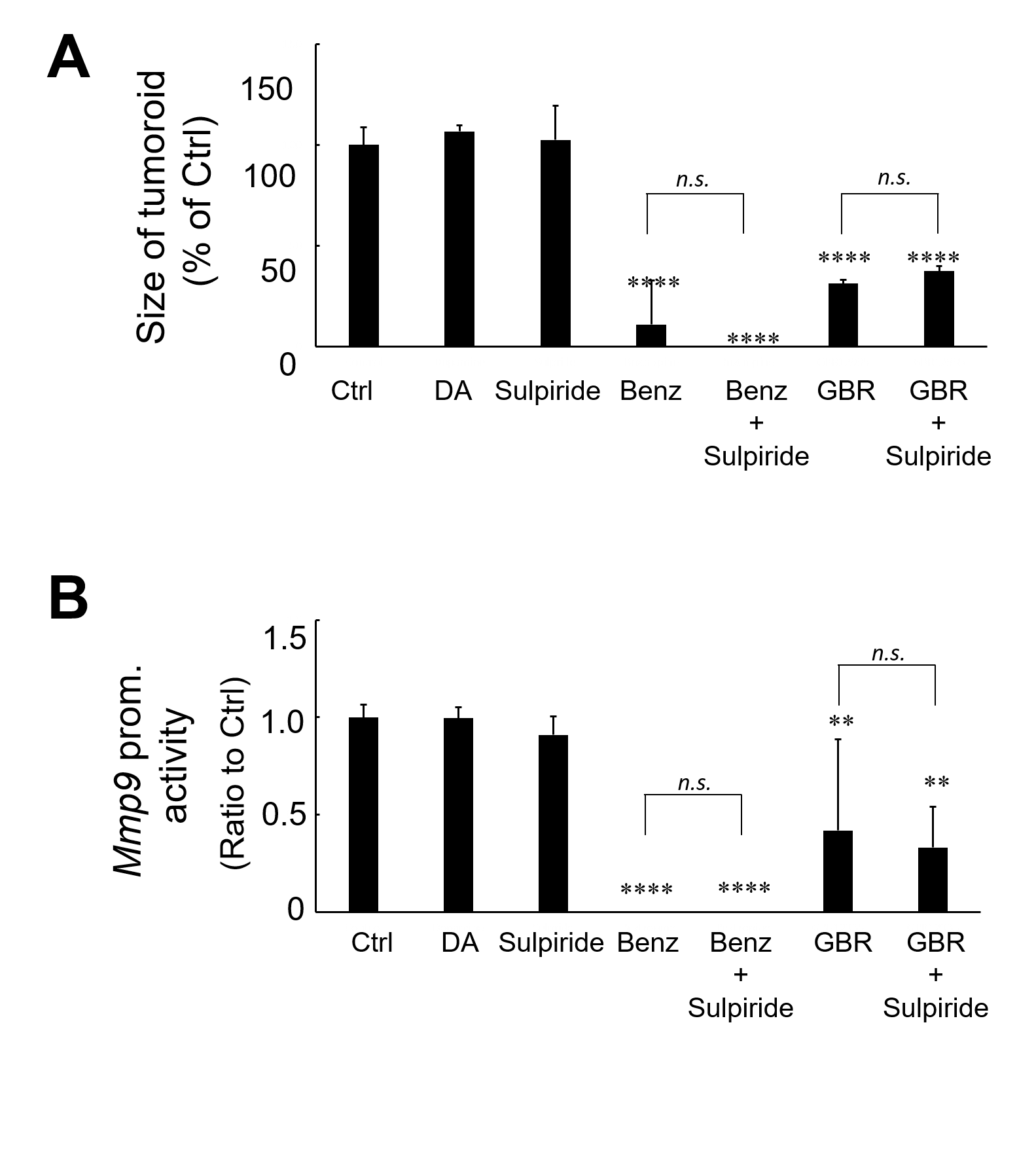


**Figure S6.** T**he size of tumoroids and MMP9 promoter activities altered by treatment with dopamine, a DR antagonist (sulpiride), GBR12935, and their combinations.** The protocol shown in Fig. 1B was used. (A) The size of the tumoroids in a well was quantified. The values were shown % of Ctrl. Mean ± SD, n=4. ****P<0.0001 vs Ctrl. (B) The values were shown as a ratio to Ctrl. Mean ± SD, n=4. **P<0.01 and ****P<0.0001 vs Ctrl.


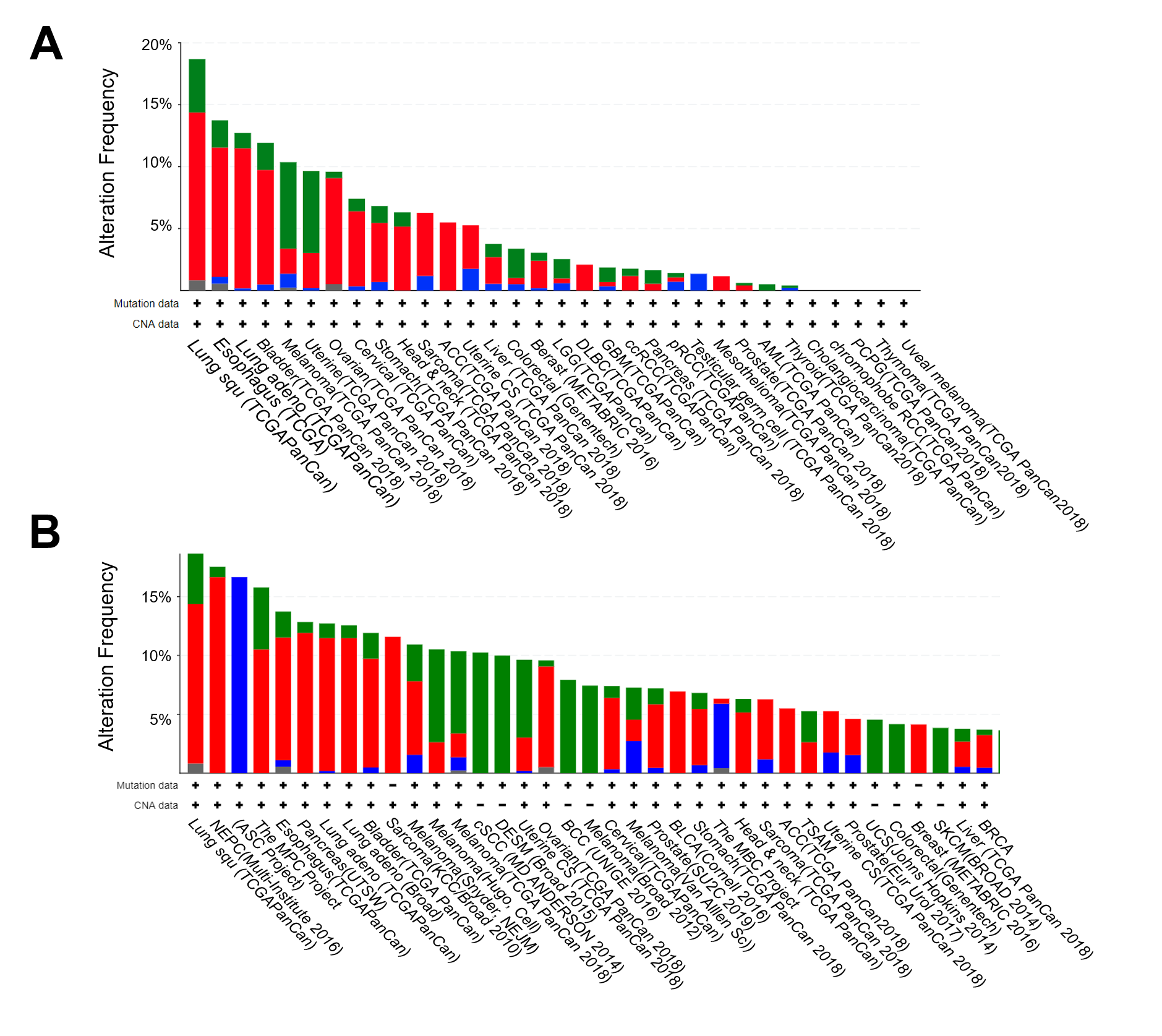


**Figure. S7. Alteration frequencies of DAT/SLC6A3 gene among cancers.** Genetic signatures of SLC6A3 gene encoding DAT was searched in cBioPortal. (A) DAT/SLC6A3 genetic alteration found in TCGA PanCancer Atlas combined study (10953 patients / 10967 samples). (B) DAT/SLC6A3 genetic alteration found in a curated set of non-redundant studies (44313 patients / 46641 samples).


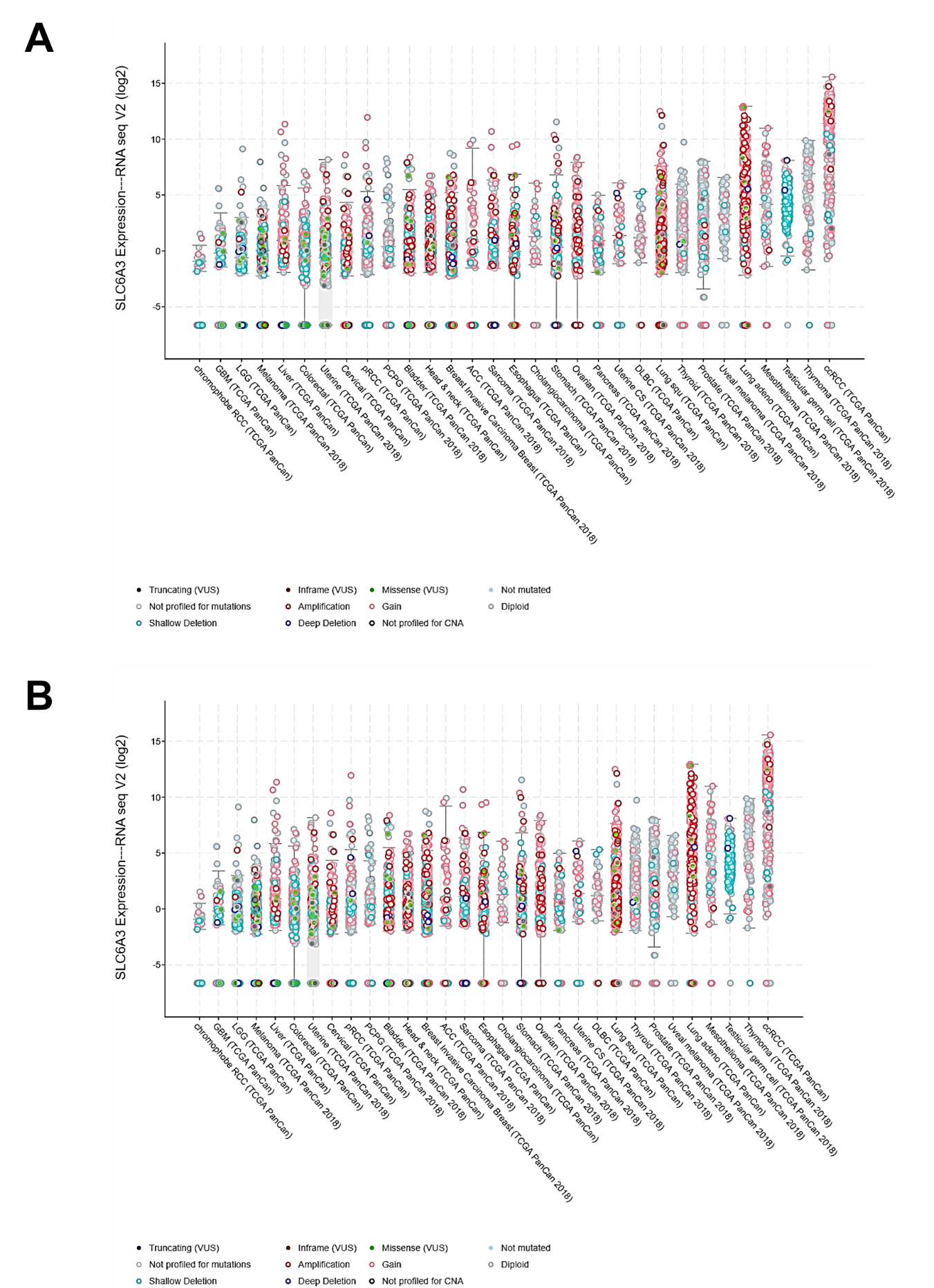


**Figure S8. SLC6A3/DAT mRNA expression among cancers.** (A) TCGA PanCancer Atlas combined study (10953 patients / 10967 samples). (B) A curated set of non-redundant studies (44313 patients / 46641 samples).


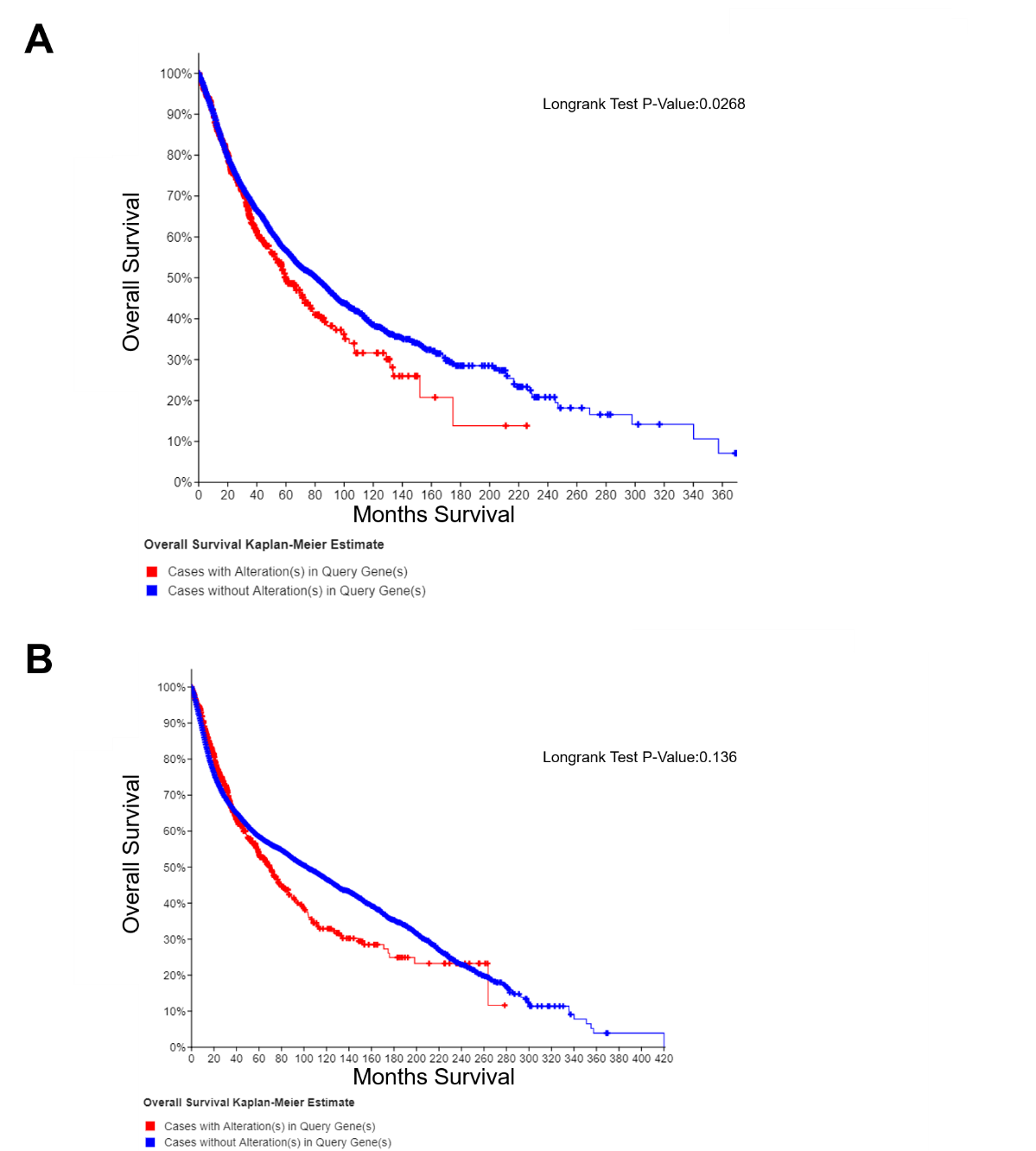


**Figure S9**. **Overall survival Kaplan-Meier estimate of cases with or without DAT/SLC6A3 alteration(s).** (A) Overall survival Kaplan-Meier estimate from TCGA PanCancer Atlas combined study data set (10953 patients / 10967 samples). (B) Overall survival Kaplan-Meier estimate from a curated set of non-redundant studies (44313 patients / 46641 samples). Red and blue lines indicate cases with or without SLC6A3 genetic alterations, respectively.

**
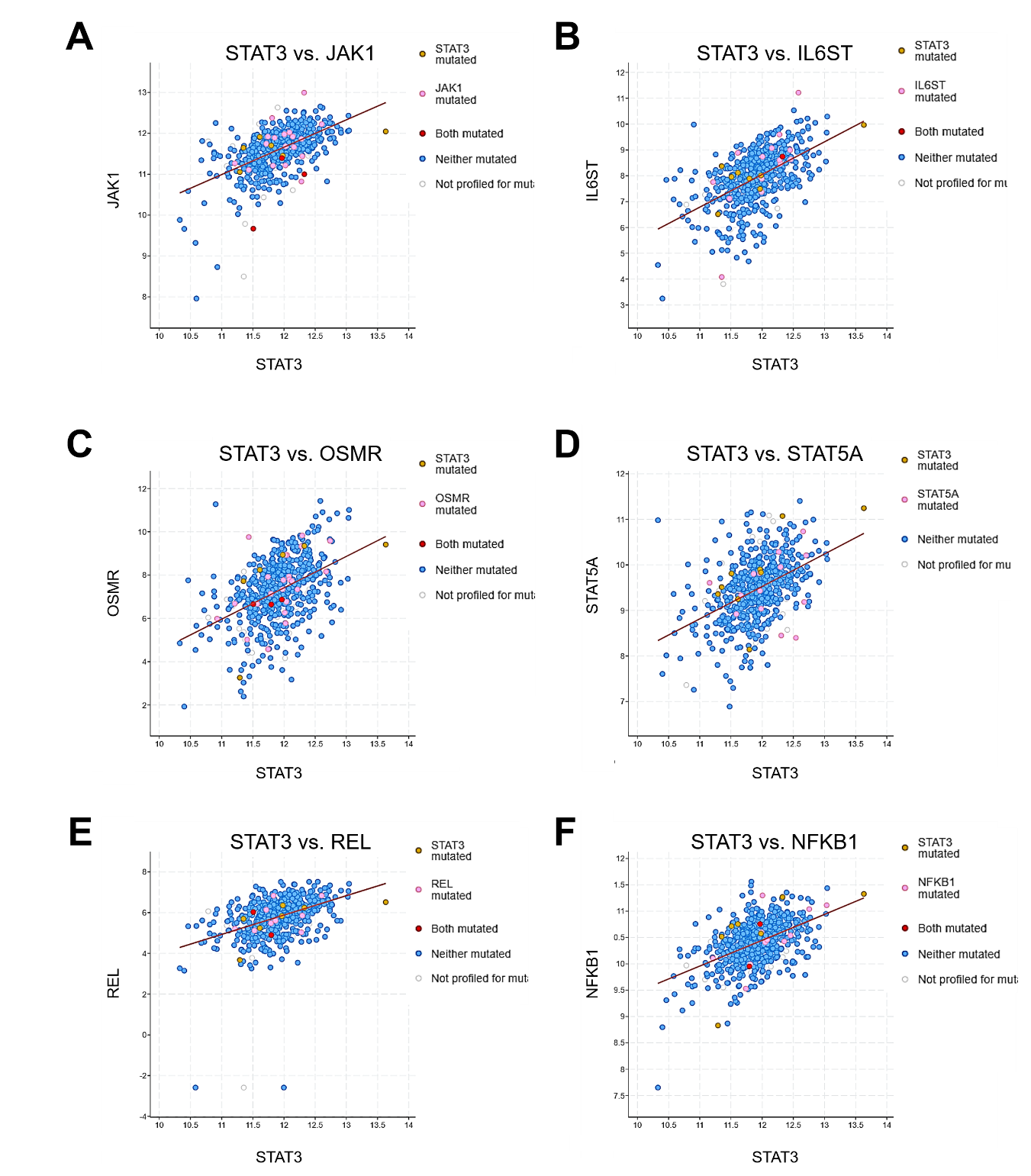
**

**Figure S10. Scatter plot analysis of coexpression correlation** **of STAT3 with NF-κB and oncostating signaling.** Data were from colorectal adenocarcinoma cases of 594 patients / 594 samples (TCGA, PanCancer Atlas). Coexpression correlation of STAT3 with JAK1 (A), IL6ST (B), OSMR (C), STAT5A (D), REL (E), and NFKB1 (F) were shown. Data were represented as log2.

**Figure S11. CTC analysis on chip.** (A to C) Pilot studies. LuM1/m9 cells (500k cells) were injected into side abdominal walls of BALB/c mice. PBS was injected as negative control. (A) Scatter plot of cell sorting. The gate of CTCs was determined by comparing tumor cells injected with PBS (mouse 1, 2, 3) vs. with LuM1/m9 cells (mouse 4, 5, 6) at 3 weeks after the injections. The gate to define CTCs was decided as area with ZsGreen-positive cells (enclosed with green line) and without cells in the PBS-treated group (enclosed with black line). The number of CTCs are shown in the gate. (B) Time-dependent change in the number of CTCs at 1 to 4 weeks after the injections. Green, ZsGreen-positive cells. (C) The number of CTCs per 40 μl blood. (D to F) The number of CTCs in LuM1/m9 tumor-injected group, LuM1/m9 tumor + Benz-injected group, and PBS-injected group. (D) Changes in the number of CTCs from 1 to 4 weeks post-allograft period. (E) The number of CTCs at 2 weeks post-allograft period. (F) The number of CTCs at 3 weeks post-allograft period. PBS, n=5; Tumor injected, n=5; Tumor+Benz, n=8.


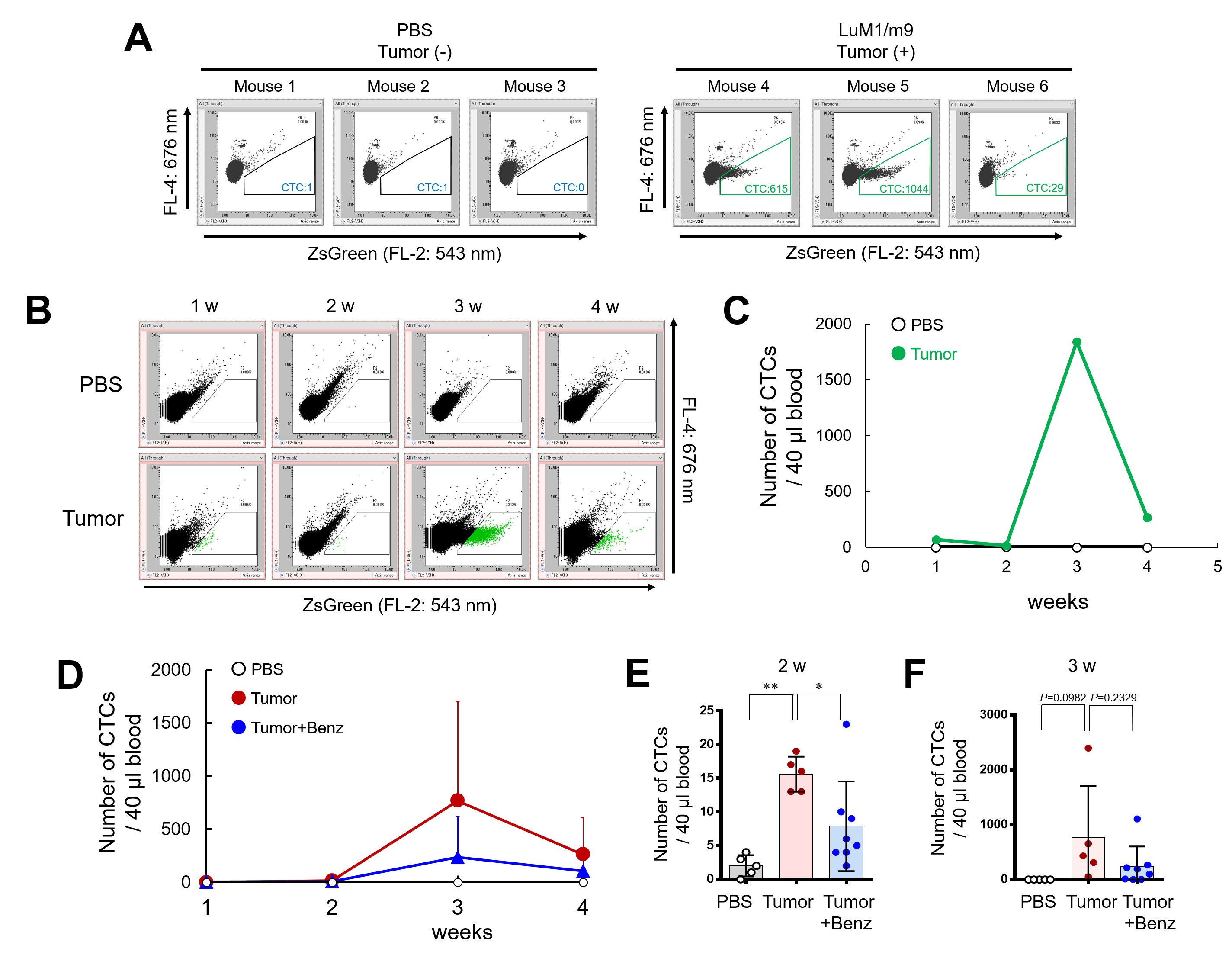


**Table S1.** Expression correlation in colorectal adenocarcinoma patients (594 cases). Data were from TCGA PanCancer Atlas and expressed as Spearman’s rank correlation coefficient and Pearson’s product-moment correlation coefficient.

|  | Spearman | Pearson |
| --- | --- | --- |
| DAT vs. BCL10 | -0.32 (p=6.85e-16) | -0.26 (p=2.93e-10) |
| STAT3 vs. JAK1 | 0.57 (p=3.08e-51) | 0.55 (p=4.69e-49) |
| STAT3 vs. IL6ST/gp130 | 0.53 (p=1.20e-43) | 0.53 (p=9.64e-44) |
| STAT3 vs. PIK3CG | 0.46 (p=9.11e-33) | 0.47 (p=2.18e-34) |
| STAT3 vs. STAT5A | 0.46 (p=4.27e-32) | 0.45 (p=3.06e-31) |
| STAT3 vs. CCR5 | 0.46 (p=7.59e-32) | 0.45 (p=2.00e-31) |
| STAT3 vs. REL | 0.45 (p=2.08e-30) | 0.41 (p=1.55e-25) |
| STAT3 vs. NOTCH2 | 0.45 (p=3.65e-30) | 0.45 (p=2.80e-30) |
| STAT3 vs. NFKB1 | 0.44 (p=1.33e-29) | 0.49 (p=2.00e-37) |
| STAT3 vs. RAB27 | 0.44 (p=4.95e-29) | 0.44 (p=1.32e-29) |
| STAT3 vs. OSMR | 0.43 (p=5.26e-28) | 0.44 (p=7.39e-30) |
| STAT3 vs. IL6R | 0.40 (p=5.70e-22) | 0.41 (p=6.05e-23) |
| STAT3 vs. IL6 | 0.24 (p=3.45e-8) | 0.23 (p=1.37e-7) |
| STAT3 vs. OSM | 0.22 (p=4.55e-8) | 0.22 (p=4.87e-8) |
| STAT3 vs. IL31RA | 0.21 (p=9.61e-7) | 0.19 (p=9.517e-6) |
| STAT3 vs. IL31 | 0.05 (p=0.387) | 0.04 (p=0.424) |
| DAT vs. STAT5B | 0.35 (p=5.04e-18) | 0.31 (p=1.70e-14) |
| DAT vs. HSPB6 | 0.33 (p=1.34e-16) | 0.33 (p=7.87e-17) |
| DAT vs. CTNNBL1 | 0.29 (p=1.22e-12) | 0.24 (p=2.98e-9) |
| DAT vs. CTNNB1 | 0.20 (p=9.94e-7) | 0.17 (p=5.225e-5) |
| DAT vs. STAT3 | 0.11 (p=8.178e-3) | 0.09 (p=0.0361) |

**Table S2.** List of primers for RT-qPCR.

| Name of primer | Sequence |
| --- | --- |
| STAT3F | 5’-CAATACCATTGACCTGCCGAT |
| STAT3R | 5’-GAGCGACTCAAACTGCCCT |
| Ctnnb1F | 5’-TCTGAGGACAAGCCACAGGA |
| Ctnnb1R | 5’-GCACCAATGTCCAGTCCAAG |
| CD326/EpcamF | 5’-GGAGTCCCTGTTCCATTCTTCT |
| CD326/EpcamR | 5’-GCGATGACTGCTAATGACACCA |
| Hif1aF | 5’-TCATCAGTTGTTGCCACTTCCCC |
| Hif1aR | 5’-ATGTAAACCATGTCGCCGTC |
| NanogF | 5’-CCTGAGCTATAAGCAGGTTAAG |
| NanogR | 5’-AATCAGACCATTGCTAGTCTTC |
| Oct4F | 5’-CGGAAGAGAAAGCGAACTAGC |
| Oct4R | 5’-ATTGGCGATGTGAGTGATCTG |
| Sox2F | 5’-GCGGAGTGGAAACTTTTGTCC |
| Sox2R | 5’-GGGAAGCGTGTACTTATCCTTCT |
