# Supplementary material for: Antiparkinson Drug Benztropine Suppresses Tumor Growth, Circulating Tumor Cells, and Metastasis by Acting on SLC6A3/DAT and Reducing STAT3
Source: Cancers (Basel). 2020 Feb 24;12(2):523. doi: 10.3390/cancers12020523 (PMC7072357; doi:10.3390/cancers12020523)
Supplement: Supplementary file 1 [file cancers-12-00523-s001.zip › Benz supple REV.pptx]

## Slide 1
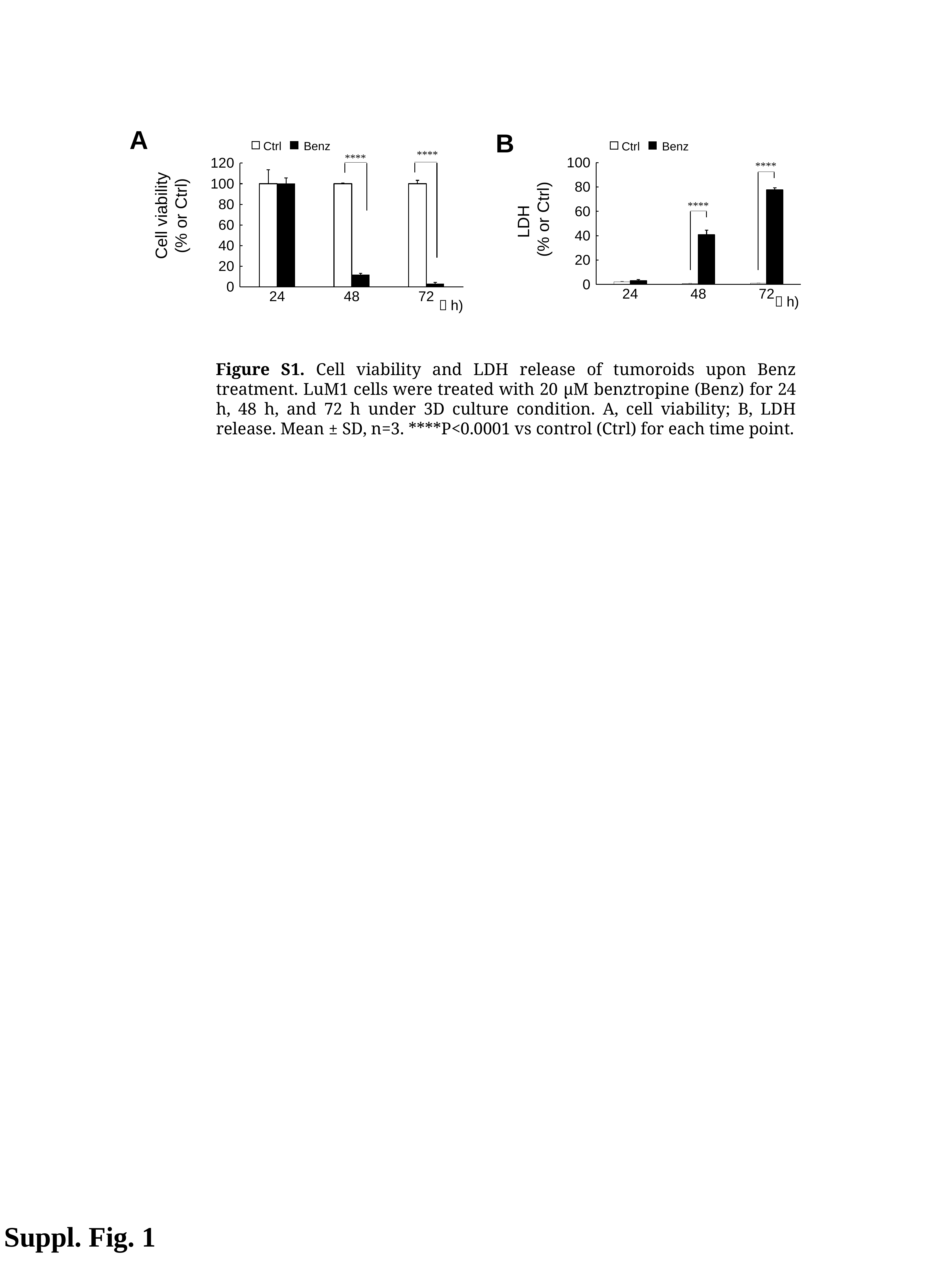

A
B
Ctrl
Benz
Ctrl
Benz
****
****
### Chart
| Category | | |
|---|---|---|
| 24 | 100.00046923453566 | 100.16304540014143 |
| 48 | 99.99978870858688 | 11.914226250845166 |
| 72 | 99.99989525948905 | 3.106999117505482 |
### Chart
| Category | | |
|---|---|---|
| 24 | 2.1604938271604954 | 2.983539094650206 |
| 48 | 0.8020050125313279 | 40.90177133655395 |
| 72 | 1.0416666666666667 | 77.6073619631902 |****
Cell viability
(% or Ctrl)
****
LDH
(% or Ctrl)
（h)
（h)
Figure S1. Cell viability and LDH release of tumoroids upon Benz treatment. LuM1 cells were treated with 20 μM benztropine (Benz) for 24 h, 48 h, and 72 h under 3D culture condition. A, cell viability; B, LDH release. Mean ± SD, n=3. ****P<0.0001 vs control (Ctrl) for each time point.
Suppl. Fig. 1

## Slide 2
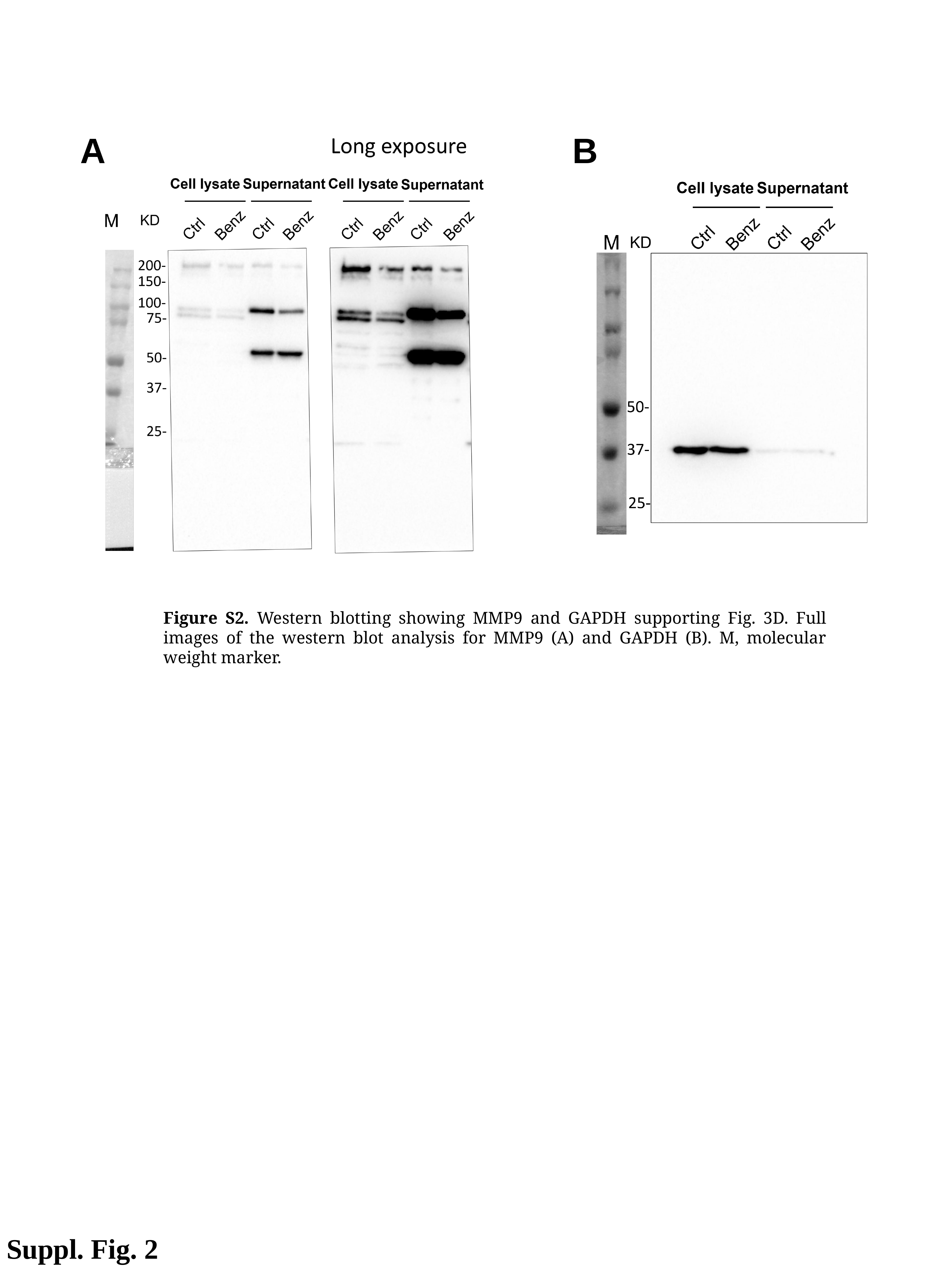

A
B
Figure S2. Western blotting showing MMP9 and GAPDH supporting Fig. 3D. Full images of the western blot analysis for MMP9 (A) and GAPDH (B). M, molecular weight marker.
Suppl. Fig. 2

## Slide 3
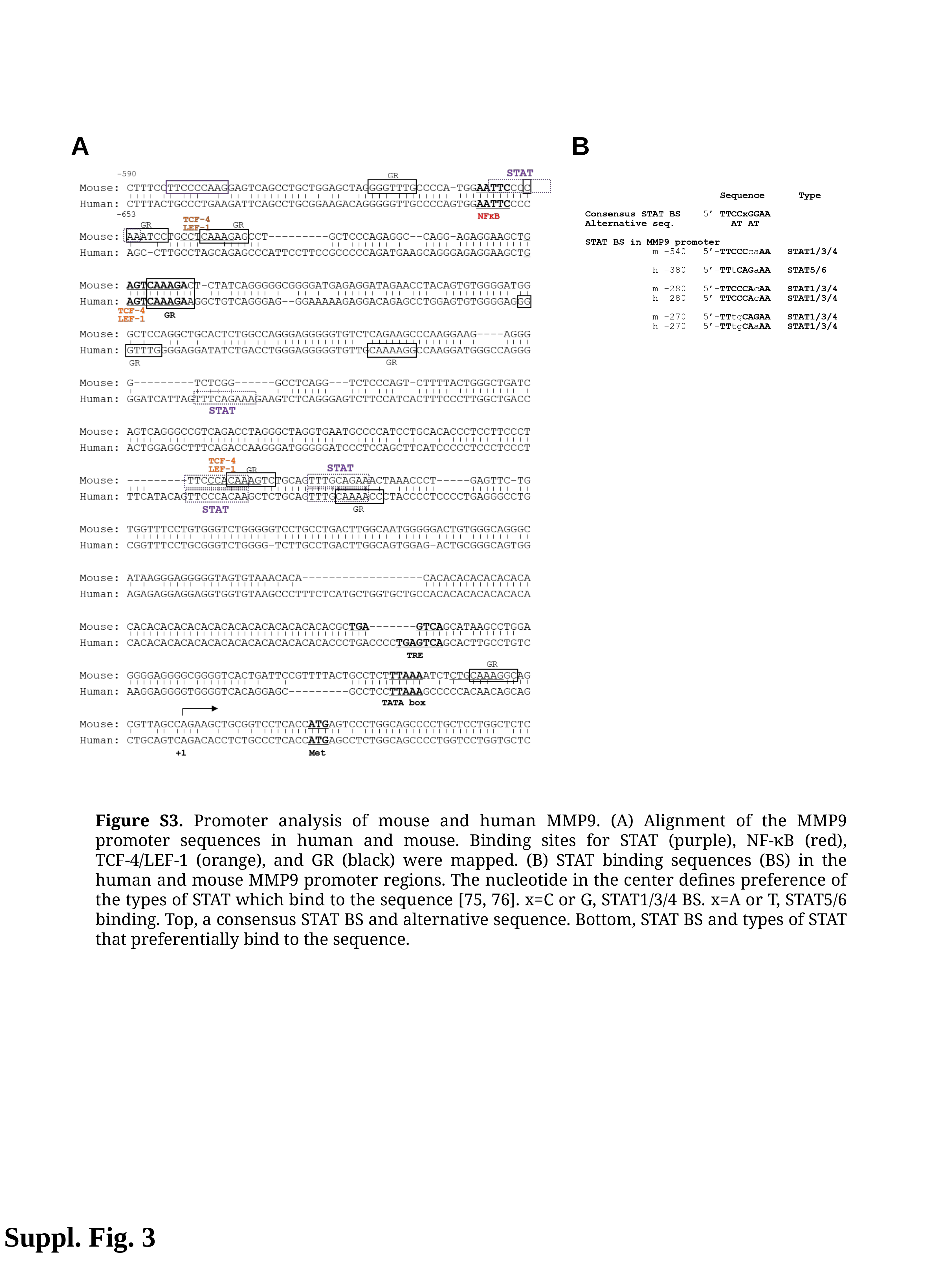

A
TCF-4
LEF-1
NF-κB
TRE
STAT4
STAT1ｂ
Human
MMP9
STAT4
STAT4
STAT4
STAT4
STAT4
+1
-600
-500
-400
-300
-200
-100
GR
GR
GR
GR
A
B
TCF-4
LEF-1
TCF-4
LEF-1
TCF-4
LEF-1
TCF-4
LEF-1
NF-κB
TRE
STAT4
STAT1ｂ
Mouse
Mmp9
STAT4
STAT1ｂ
STAT5A
STAT4
STAT4
STAT4
STAT4
STAT4
STAT4
+1
-600
-500
-400
-300
-200
-100
GR
GR
GR
GR
GR
GR
Consensus STAT bs.	5’-TTCCxGGAA
Alternative seq.		 AT AT
MMP9 promoter region
		m -540	5’-TTCCCcaAA
		h -380	5’-TTtCAGaAA
		m -280	5’-TTCCCAcAA
		h -280	5’-TTCCCAcAA
		m -270	5’-TTtgCAGAA
		h -270	5’-TTtgCAaAA
Figure S3. Promoter analysis of mouse and human MMP9. (A) Alignment of the MMP9 promoter sequences in human and mouse. Binding sites for STAT (purple), NF-κB (red), TCF-4/LEF-1 (orange), and GR (black) were mapped. (B) STAT binding sequences (BS) in the human and mouse MMP9 promoter regions. The nucleotide in the center defines preference of the types of STAT which bind to the sequence [75, 76]. x=C or G, STAT1/3/4 BS. x=A or T, STAT5/6 binding. Top, a consensus STAT BS and alternative sequence. Bottom, STAT BS and types of STAT that preferentially bind to the sequence.
Suppl. Fig. 3

## Slide 4
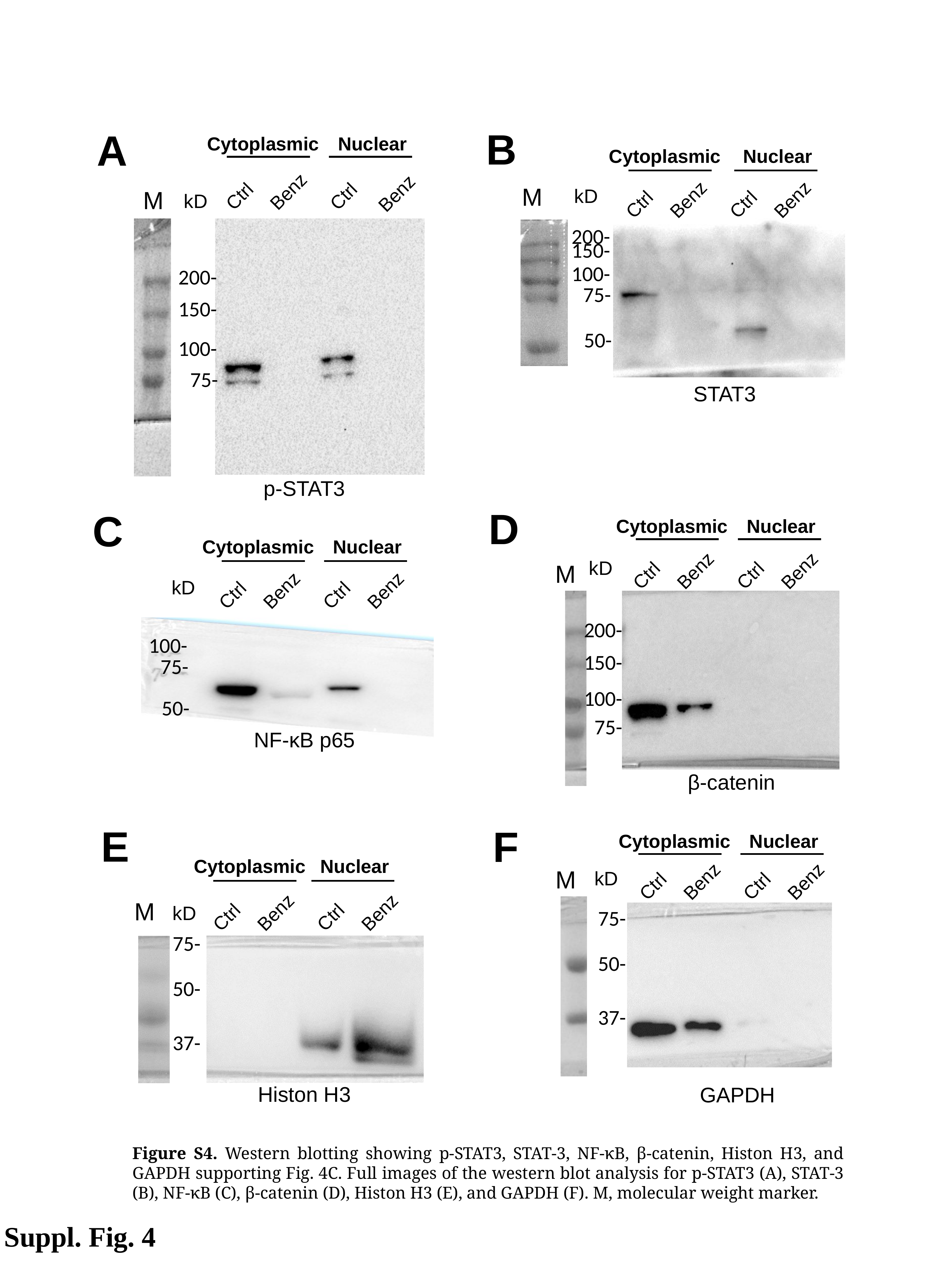

B
A
Cytoplasmic
Nuclear
Cytoplasmic
Nuclear
Benz
Benz
Benz
Benz
Ctrl
Ctrl
Ctrl
Ctrl
M
kD
M
kD
200-
150-
100-
75-
50-
200-
150-
100-
75-
STAT3
p-STAT3
D
C
Cytoplasmic
Nuclear
Cytoplasmic
Nuclear
Benz
Benz
Ctrl
Ctrl
kD
Benz
Benz
M
Ctrl
Ctrl
kD
200-
150-
100-
75-
100-
75-
50-
NF-κB p65
β-catenin
E
F
Cytoplasmic
Nuclear
Benz
Benz
Cytoplasmic
Nuclear
Ctrl
Ctrl
M
kD
Benz
Benz
Ctrl
Ctrl
M
kD
75-
50-
37-
75-
50-
37-
Histon H3
GAPDH
Figure S4. Western blotting showing p-STAT3, STAT-3, NF-κB, β-catenin, Histon H3, and GAPDH supporting Fig. 4C. Full images of the western blot analysis for p-STAT3 (A), STAT-3 (B), NF-κB (C), β-catenin (D), Histon H3 (E), and GAPDH (F). M, molecular weight marker.
Suppl. Fig. 4

## Slide 5
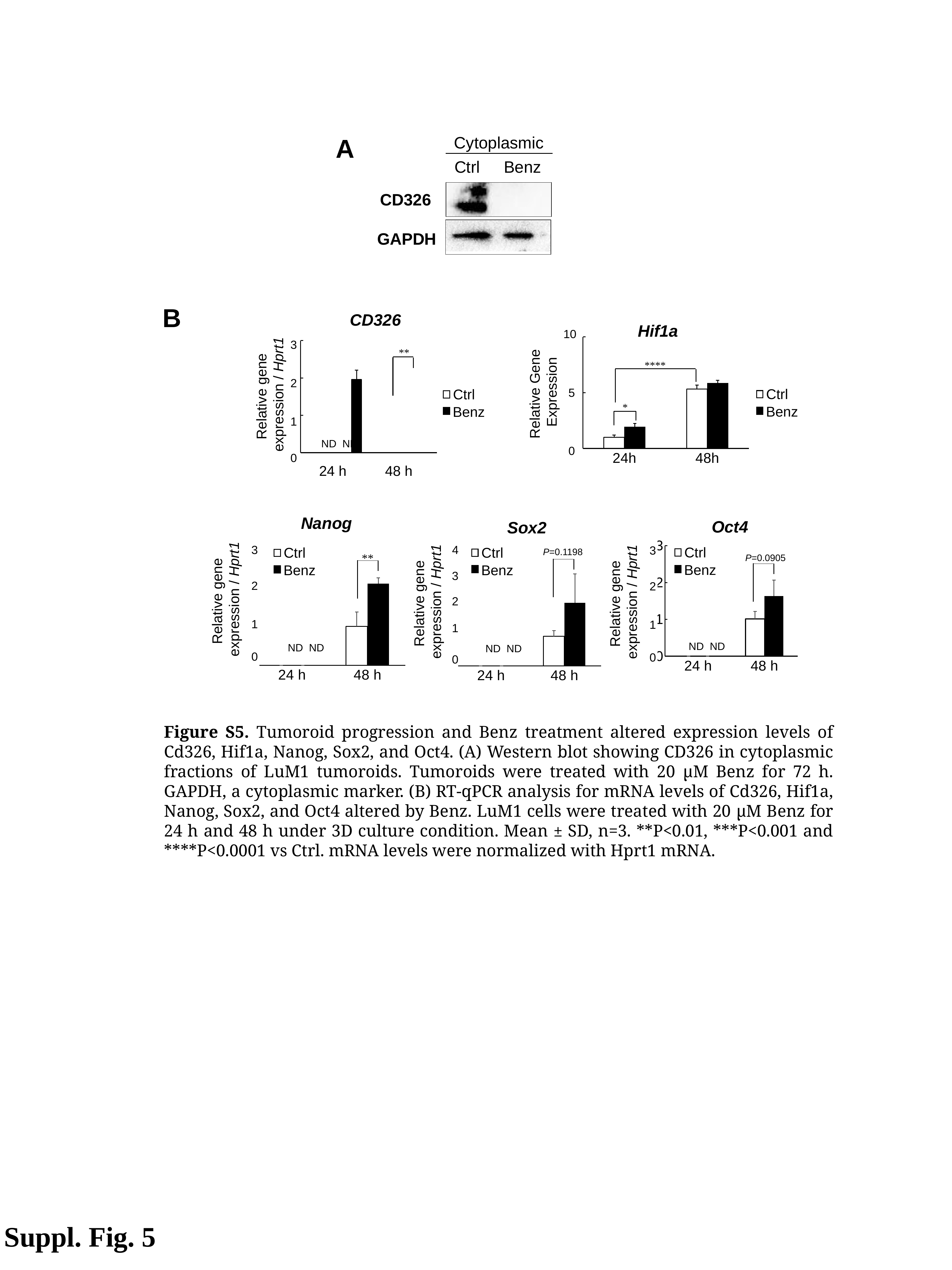

A
Cytoplasmic
Ctrl
Benz
CD326
GAPDH
B
CD326
### Chart
| Category | | |
|---|---|---|
| 24h | 1.0090028488811271 | 1.924337761880663 |
| 48h | 5.32275289683147 | 5.859719707525581 |Hif1a
10
****
Relative Gene
Expression
5
*
0
### Chart
| Category | | |
|---|---|---|
| 24h | 0.0 | 0.0 |
| 48h | 1.0110124682594972 | 1.9622158460379489 |3
**
### Chart: OCT4
| Category | |
|---|---|
| Ctrl | 1.0110807044122125 |
| Benz | 1.6326407765281419 |
### Chart: EpCAM
| Category | |
|---|---|
| Ctrl | 1.0110124682594972 |
| Benz | 1.9622158460379489 |
### Chart: SOX2
| Category | |
|---|---|
| Ctrl | 1.0186211708068698 |
| Benz | 2.1732829461436514 |
### Chart: Nanog
| Category | |
|---|---|
| Ctrl | 1.0 |
| Benz | 2.1 |2
Relative gene
expression / Hprt1
Ctrl
Benz
Ctrl
Benz
1
ND ND
0
24 h
48 h
Nanog
Oct4
Sox2
### Chart
| Category | | |
|---|---|---|
| 24 h | 0.0 | 0.0 |
| 48 h | 1.0110807044122125 | 1.6326407765281419 |
### Chart
| Category | | |
|---|---|---|
| 24 h | 0.0 | 0.0 |
| 48 h | 1.0 | 2.1 |3
### Chart
| Category | | |
|---|---|---|
| 24 h | 0.0 | 0.0 |
| 48 h | 1.0186211708068698 | 2.1732829461436514 |4
3
Ctrl
Benz
Ctrl
Benz
Ctrl
Benz
P=0.1198
**
P=0.0905
3
2
2
Relative gene
expression / Hprt1
Relative gene
expression / Hprt1
Relative gene
expression / Hprt1
2
1
1
1
ND ND
ND ND
ND ND
0
0
0
Figure S5. Tumoroid progression and Benz treatment altered expression levels of Cd326, Hif1a, Nanog, Sox2, and Oct4. (A) Western blot showing CD326 in cytoplasmic fractions of LuM1 tumoroids. Tumoroids were treated with 20 μM Benz for 72 h. GAPDH, a cytoplasmic marker. (B) RT-qPCR analysis for mRNA levels of Cd326, Hif1a, Nanog, Sox2, and Oct4 altered by Benz. LuM1 cells were treated with 20 μM Benz for 24 h and 48 h under 3D culture condition. Mean ± SD, n=3. **P<0.01, ***P<0.001 and ****P<0.0001 vs Ctrl. mRNA levels were normalized with Hprt1 mRNA.
### Chart
| Category | |
|---|---|
| Ctrl | 1.0 |
| Benz | 2.1 |
### Chart
| Category | |
|---|---|
| Ctrl | 1.0186211708068698 |
| Benz | 2.1732829461436514 |Oct4
Sox2
Nanog
### Chart
| Category | |
|---|---|
| Ctrl | 1.0110807044122125 |
| Benz | 1.6326407765281419 |P=0.1198
**
P=0.0905
Relative Gene
Expression
P=0.1198
**
P=0.0905
Suppl. Fig. 5

## Slide 6
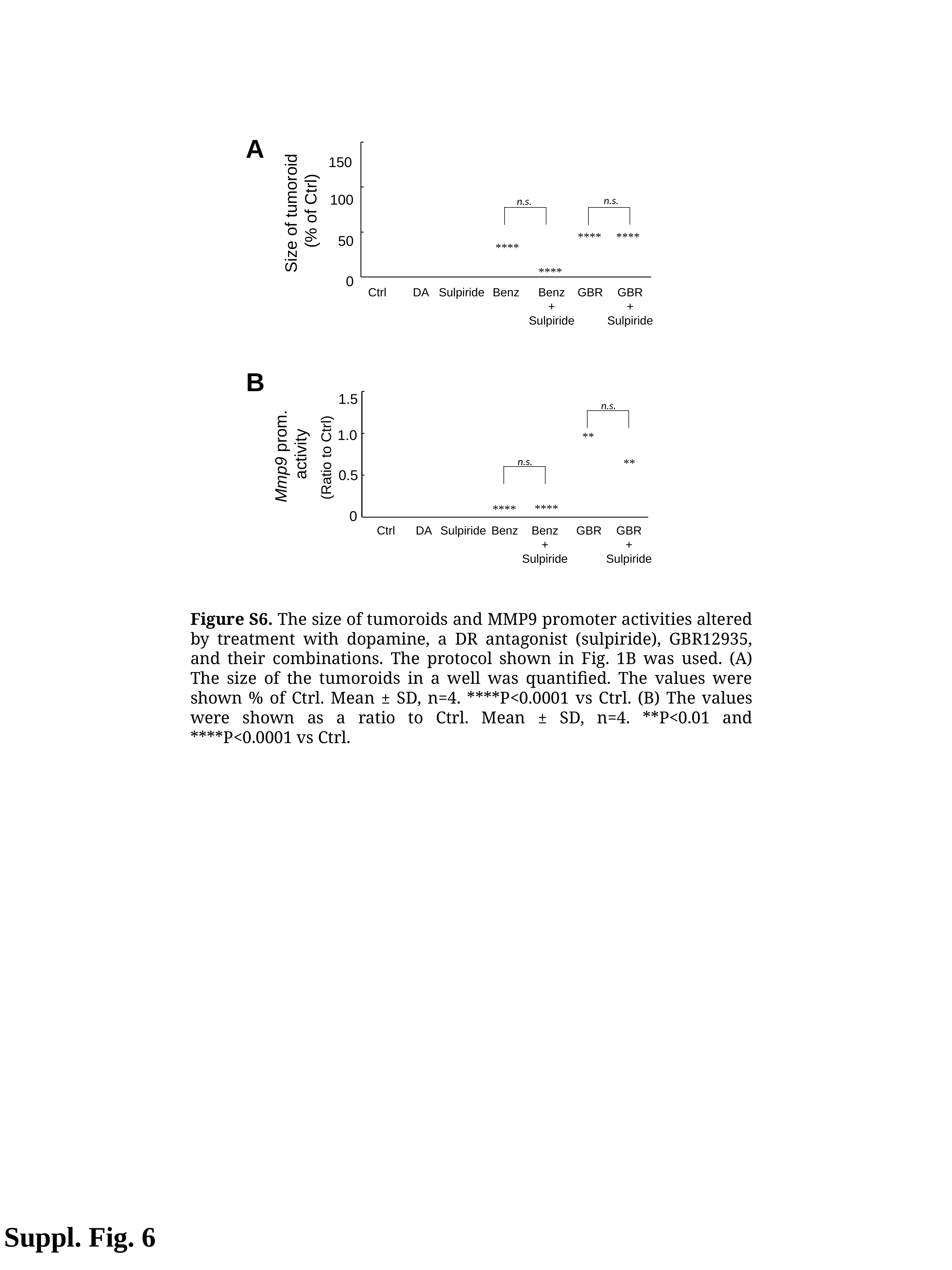

A
### Chart
| Category | |
|---|---|
| Control | 100.00188768286927 |
| Dopamine | 106.70056630486079 |
| Sulpride | 102.33907503539405 |
| Bnztropine | 10.901368570080226 |
| Bnztropine | 0.0 |
| GBR12935 | 31.17862199150543 |
| GBR12935 | 37.3112317130722 |150
n.s.
n.s.
100
Size of tumoroid
 (% of Ctrl)
****
****
50
****
****
0
Ctrl
DA
Sulpiride
Benz
Benz
+
Sulpiride
GBR
GBR
+
Sulpiride
### Chart
| Category | |
|---|---|
| Control | 0.9999089076639438 |
| Dopamine | 0.9951444805458493 |
| Sulpride | 0.9097587237715047 |
| Bnztropine | 0.0 |
| Bnztropine | 0.0 |
| GBR12935 | 0.41811295736336274 |
| GBR12935 | 0.33152829560023933 |
n.s.
1.5
1.0
**
Mmp9 prom.
 activity
n.s.
(Ratio to Ctrl)
**
0.5
****
****
0
Ctrl
DA
Sulpiride
Benz
Benz
+
Sulpiride
GBR
GBR
+
Sulpiride
B
Figure S6. The size of tumoroids and MMP9 promoter activities altered by treatment with dopamine, a DR antagonist (sulpiride), GBR12935, and their combinations. The protocol shown in Fig. 1B was used. (A) The size of the tumoroids in a well was quantified. The values were shown % of Ctrl. Mean ± SD, n=4. ****P<0.0001 vs Ctrl. (B) The values were shown as a ratio to Ctrl. Mean ± SD, n=4. **P<0.01 and ****P<0.0001 vs Ctrl.
Suppl. Fig. 6

## Slide 7
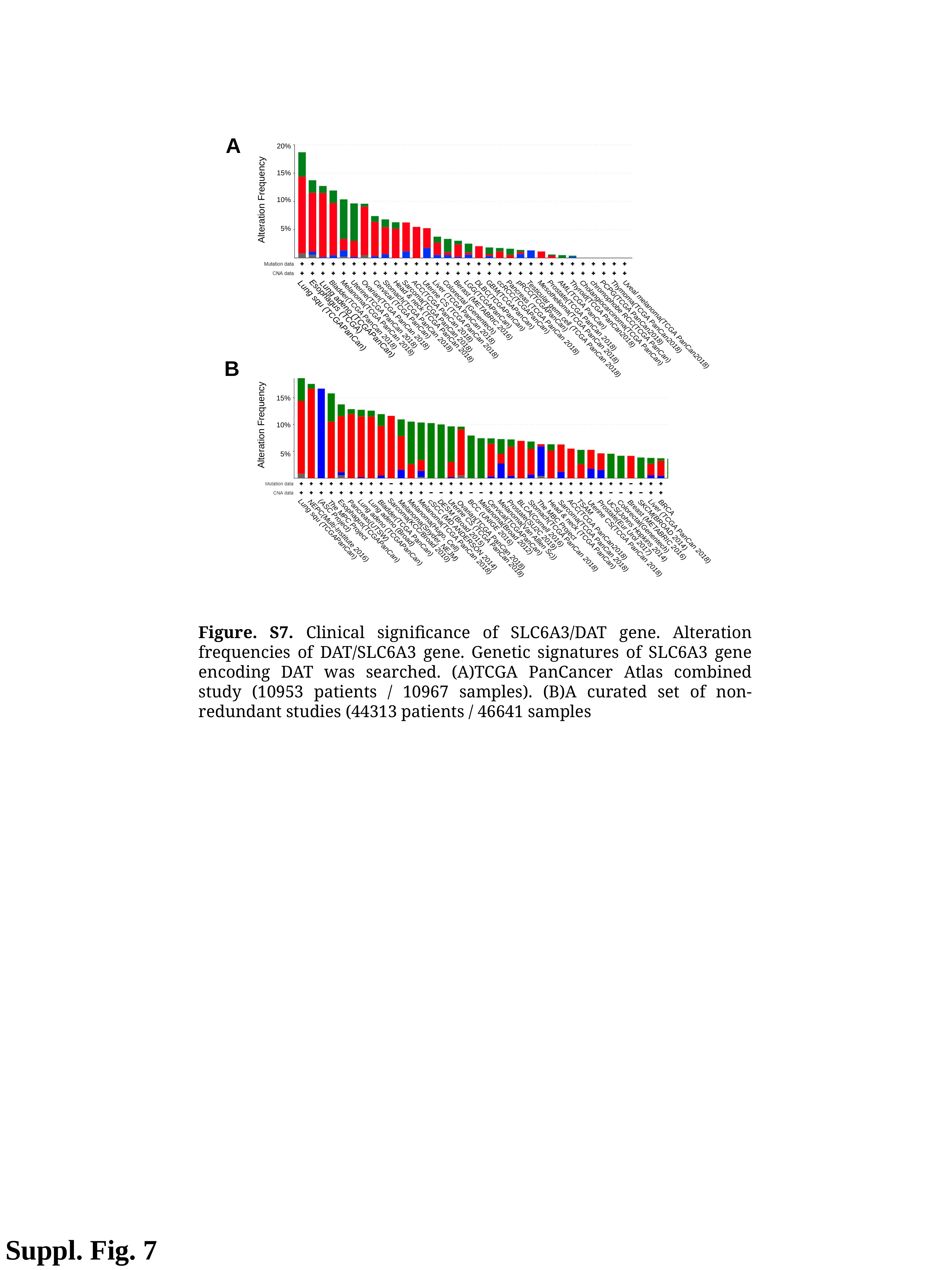

A
20%
15%
Alteration Frequency
10%
5%
stat3
tcga pancancer
LGG(TCGAPanCan)
GBM(TCGAPanCan)
AML(TCGA PanCan)
DLBC(TCGAPanCan)
pRCC(TCGAPanCan)
Esophagus (TCGA)
ccRCC(TCGAPanCan)
Colorectal (Genentech)
Cervical (TCGA PanCan)
Berast (METABRIC 2016)
ACC(TCGA PanCan 2018)
Liver (TCGA PanCan 2018)
Thyroid(TCGA PanCan2018)
Uterine(TCGA PanCan 2018)
Bladder(TCGA PanCan 2018)
Lung squ (TCGAPanCan)
Ovarian(TCGA PanCan 2018)
Prostate(TCGA PanCan 2018)
Stomach(TCGA PanCan 2018)
Sarcoma(TCGA PanCan 2018)
Pancreas (TCGA PanCan 2018)
Melanoma(TCGA PanCan 2018)
Lung adeno (TCGAPanCan)
Uterine CS (TCGA PanCan 2018)
PCPG(TCGA PanCan2018)
Thymoma(TCGA PanCan2018)
Head & neck (TCGA PanCan 2018)
chromophobe RCC(TCGA PanCan)
Mesothelioma(TCGA PanCan 2018)
Cholangiocarcinoma(TCGA PanCan)
Uveal melanoma(TCGA PanCan2018)
Testicular germ cell (TCGA PanCan 2018)
B
15%
Alteration Frequency
10%
5%
TSAM
BRCA
(ASC Project)
The MBC Project
The MPC Project
Pancreas(UTSW)
BCC (UNIGE 2016)
Lung adeno (Broad)
DESM (Broad 2015)
BLCA(Cornell 2016)
SKCM(BROAD 2014)
Prostate(SU2C 2019)
Melanoma(Hugo, Cell)
Colorectal(Genentech)
Melanoma(Broad 2012)
Cervical(TCGAPanCan)
Prostate(Eur Urol 2017)
Bladder(TCGA PanCan)
Lung squ (TCGAPanCan)
ACC(TCGA PanCan2018)
Melanoma(Van Alllen Sc))
Breast (METABRIC 2016)
Sarcoma(KCC/Broad 2010)
Melanoma(Snyder, NEJM)
UCS(Johns Hopkins 2014)
NEPC(Multi-Institute 2016)
Esophagus(TCGAPanCan)
Liver (TCGA PanCan 2018)
Lung adeno (TCGAPanCan)
Head & neck (TCGA PanCan)
cSCC (MD ANDERSON 2014)
Ovarian(TCGA PanCan 2018)
Stomach(TCGA PanCan 2018)
Sarcoma(TCGA PanCan 2018)
Melanoma(TCGA PanCan 2018)
Uterine CS(TCGA PanCan 2018)
Uterine CS (TCGA PanCan 2018)
Figure. S7. Clinical significance of SLC6A3/DAT gene. Alteration frequencies of DAT/SLC6A3 gene. Genetic signatures of SLC6A3 gene encoding DAT was searched. (A)TCGA PanCancer Atlas combined study (10953 patients / 10967 samples). (B)A curated set of non-redundant studies (44313 patients / 46641 samples
Suppl. Fig. 7

## Slide 8
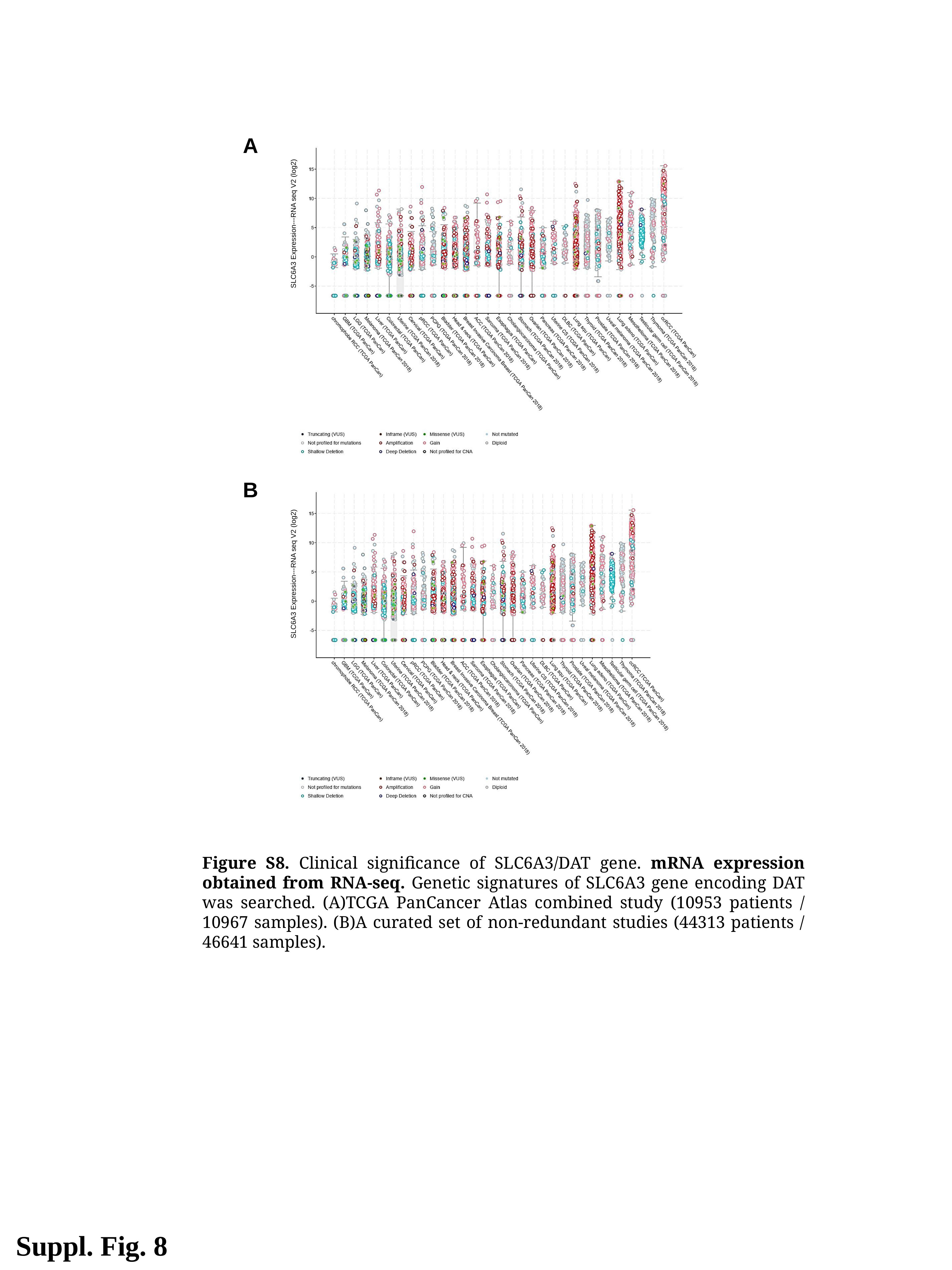

A
SLC6A3 Expression---RNA seq V2 (log2)
B
SLC6A3 Expression---RNA seq V2 (log2)
Figure S8. Clinical significance of SLC6A3/DAT gene. mRNA expression obtained from RNA-seq. Genetic signatures of SLC6A3 gene encoding DAT was searched. (A)TCGA PanCancer Atlas combined study (10953 patients / 10967 samples). (B)A curated set of non-redundant studies (44313 patients / 46641 samples).
Suppl. Fig. 8

## Slide 9
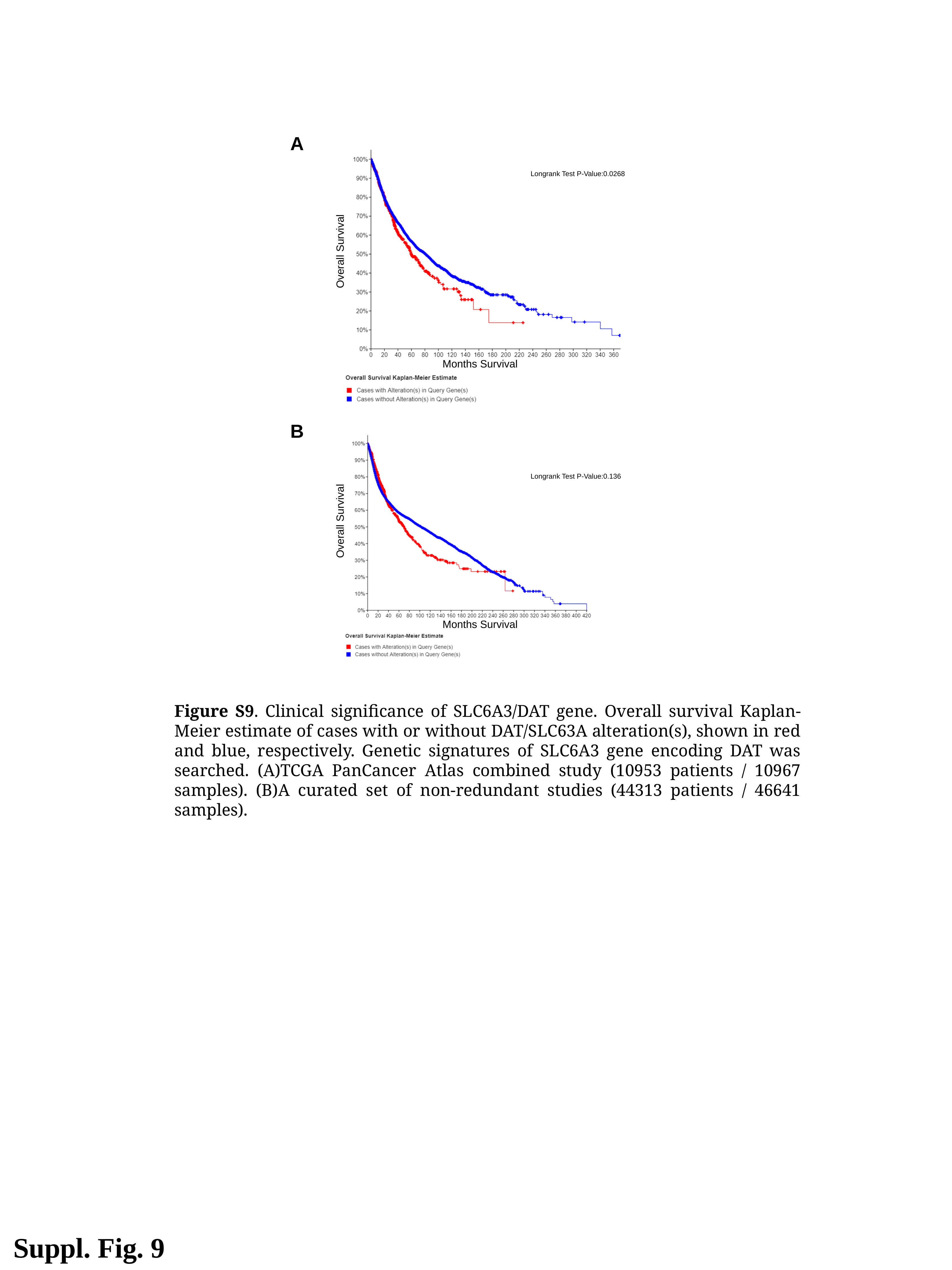

A
Longrank Test P-Value:0.0268
Overall Survival
Months Survival
B
Longrank Test P-Value:0.136
Overall Survival
Months Survival
Figure S9. Clinical significance of SLC6A3/DAT gene. Overall survival Kaplan-Meier estimate of cases with or without DAT/SLC63A alteration(s), shown in red and blue, respectively. Genetic signatures of SLC6A3 gene encoding DAT was searched. (A)TCGA PanCancer Atlas combined study (10953 patients / 10967 samples). (B)A curated set of non-redundant studies (44313 patients / 46641 samples).
Suppl. Fig. 9

## Slide 10
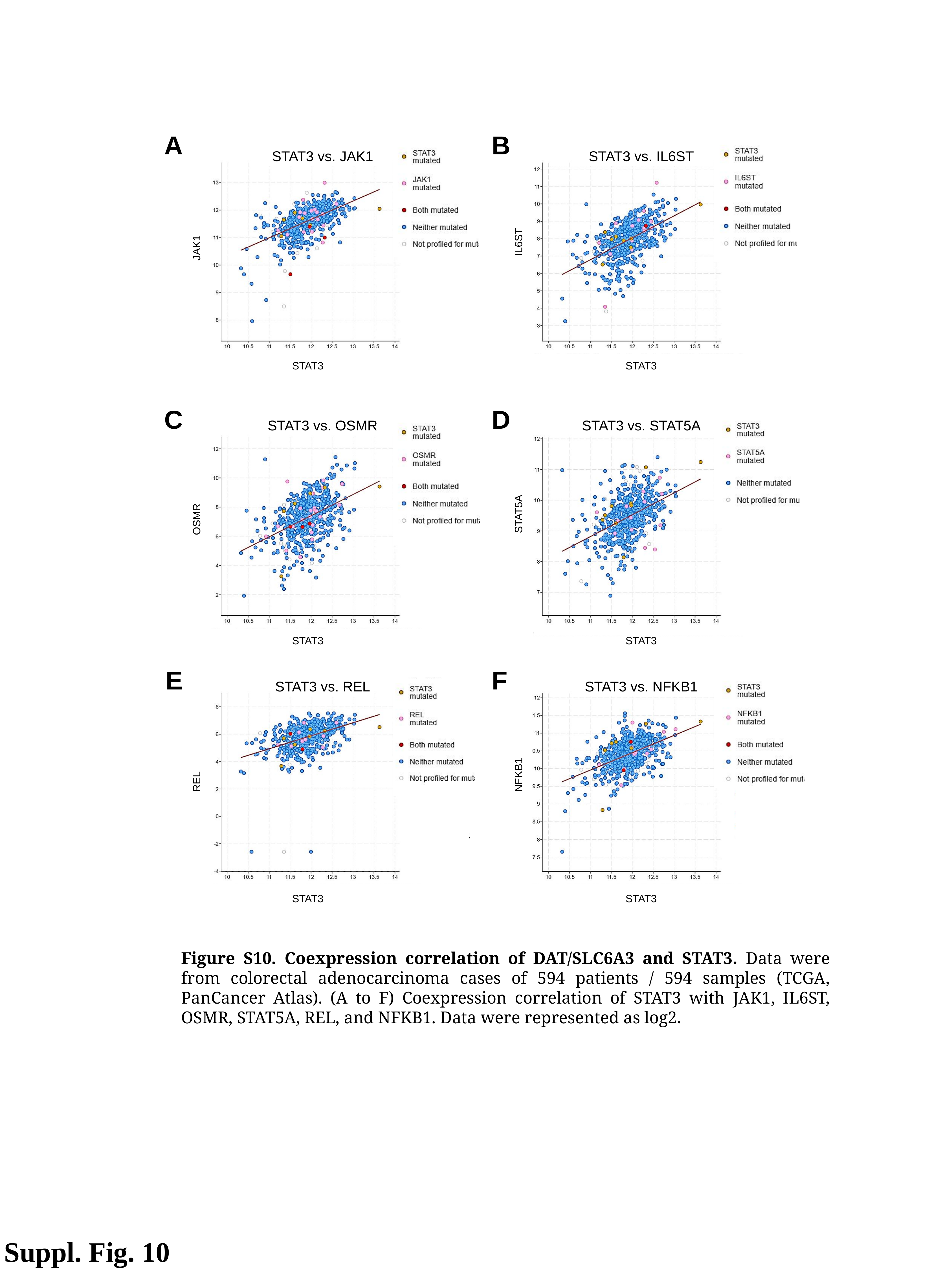

A
B
STAT3 vs. JAK1
STAT3 vs. IL6ST
IL6ST
JAK1
STAT3
STAT3
C
D
STAT3 vs. OSMR
STAT3 vs. STAT5A
STAT5A
OSMR
STAT3
STAT3
E
F
STAT3 vs. REL
STAT3 vs. NFKB1
NFKB1
REL
STAT3
STAT3
Figure S10. Coexpression correlation of DAT/SLC6A3 and STAT3. Data were from colorectal adenocarcinoma cases of 594 patients / 594 samples (TCGA, PanCancer Atlas). (A to F) Coexpression correlation of STAT3 with JAK1, IL6ST, OSMR, STAT5A, REL, and NFKB1. Data were represented as log2.
Suppl. Fig. 10
ｃ－Rel/RelA heterodimer --> Bcl-2, survival
ｃ－Rel homo dimer --> Bcl-X, IL-6 survival

## Slide 11
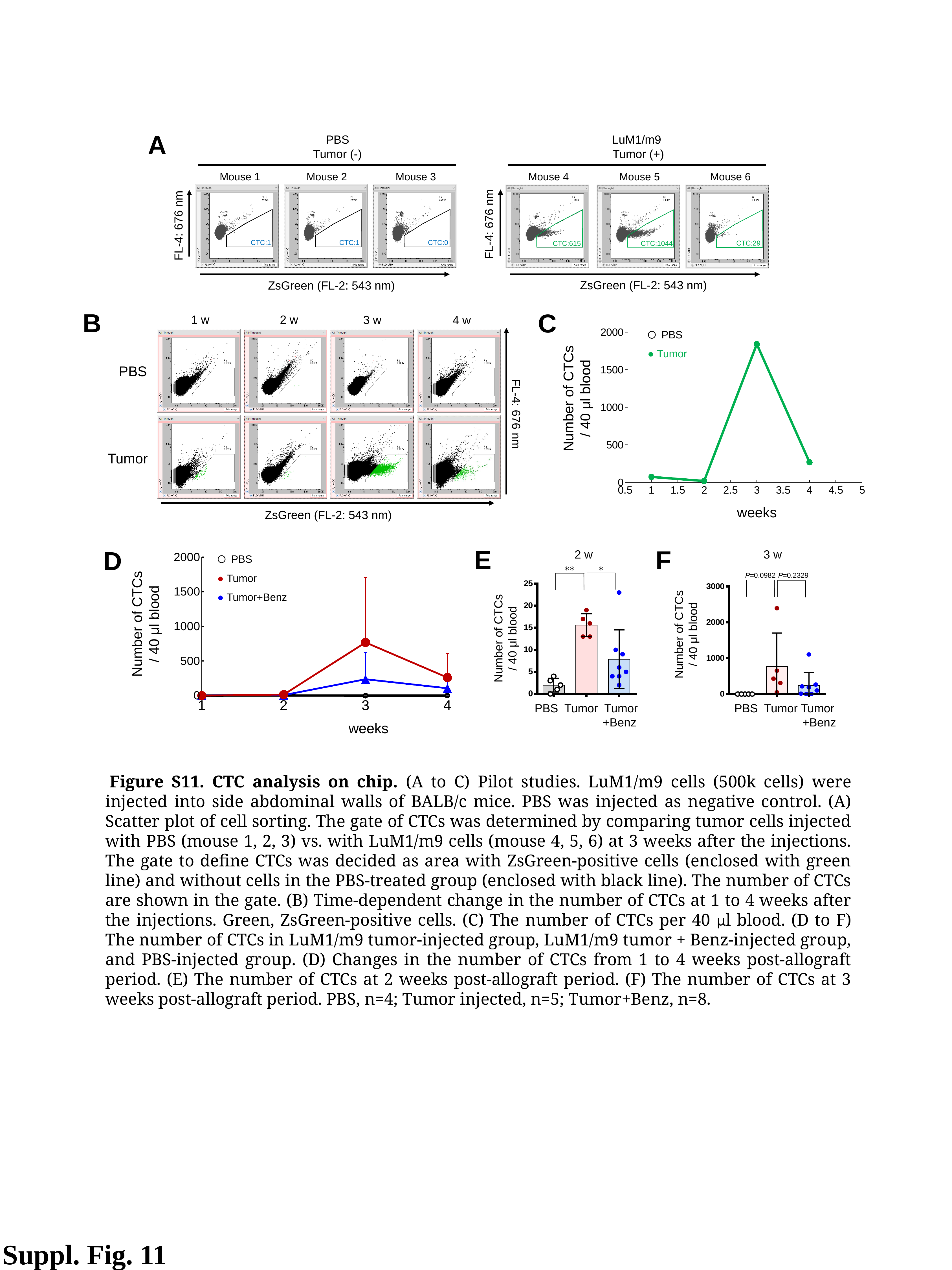

A
PBS
Tumor (-)
LuM1/m9
Tumor (+)
Mouse 1
Mouse 2
Mouse 3
Mouse 4
Mouse 5
Mouse 6
FL-4: 676 nm
FL-4: 676 nm
CTC:1
CTC:1
CTC:0
CTC:29
CTC:615
CTC:1044
D
3 w
ZsGreen (FL-2: 543 nm)
ZsGreen (FL-2: 543 nm)
P=0.0062
P=0.0389
B
C
1 w
2 w
3 w
4 w
〇 PBS
● Tumor
### Chart
| Category | | |
|---|---|---|
Number of CTCs / 40 μl blood
PBS
Number of CTCs
/ 40 μl blood
FL-4: 676 nm
Tumor
PBS Tumor Tumor
 +Benz
weeks
ZsGreen (FL-2: 543 nm)
〇 PBS
● Tumor
● Tumor+Benz
### Chart
| Category | | | |
|---|---|---|---|Number of CTCs
/ 40 μl blood
weeks
E
F
D
2 w
3 w
**
*
P=0.0982
P=0.2329
Number of CTCs
/ 40 μl blood
Number of CTCs
/ 40 μl blood
 PBS Tumor Tumor
 +Benz
PBS Tumor Tumor
 +Benz
 Figure S11. CTC analysis on chip. (A to C) Pilot studies. LuM1/m9 cells (500k cells) were injected into side abdominal walls of BALB/c mice. PBS was injected as negative control. (A) Scatter plot of cell sorting. The gate of CTCs was determined by comparing tumor cells injected with PBS (mouse 1, 2, 3) vs. with LuM1/m9 cells (mouse 4, 5, 6) at 3 weeks after the injections. The gate to define CTCs was decided as area with ZsGreen-positive cells (enclosed with green line) and without cells in the PBS-treated group (enclosed with black line). The number of CTCs are shown in the gate. (B) Time-dependent change in the number of CTCs at 1 to 4 weeks after the injections. Green, ZsGreen-positive cells. (C) The number of CTCs per 40 μl blood. (D to F) The number of CTCs in LuM1/m9 tumor-injected group, LuM1/m9 tumor + Benz-injected group, and PBS-injected group. (D) Changes in the number of CTCs from 1 to 4 weeks post-allograft period. (E) The number of CTCs at 2 weeks post-allograft period. (F) The number of CTCs at 3 weeks post-allograft period. PBS, n=4; Tumor injected, n=5; Tumor+Benz, n=8.
Suppl. Fig. 11
