# Supplementary figures and images for: Antiparkinson Drug Benztropine Suppresses Tumor Growth, Circulating Tumor Cells, and Metastasis by Acting on SLC6A3/DAT and Reducing STAT3
Source: Cancers (Basel). 2020 Feb 24;12(2):523. doi: 10.3390/cancers12020523 (PMC7072357; doi:10.3390/cancers12020523)

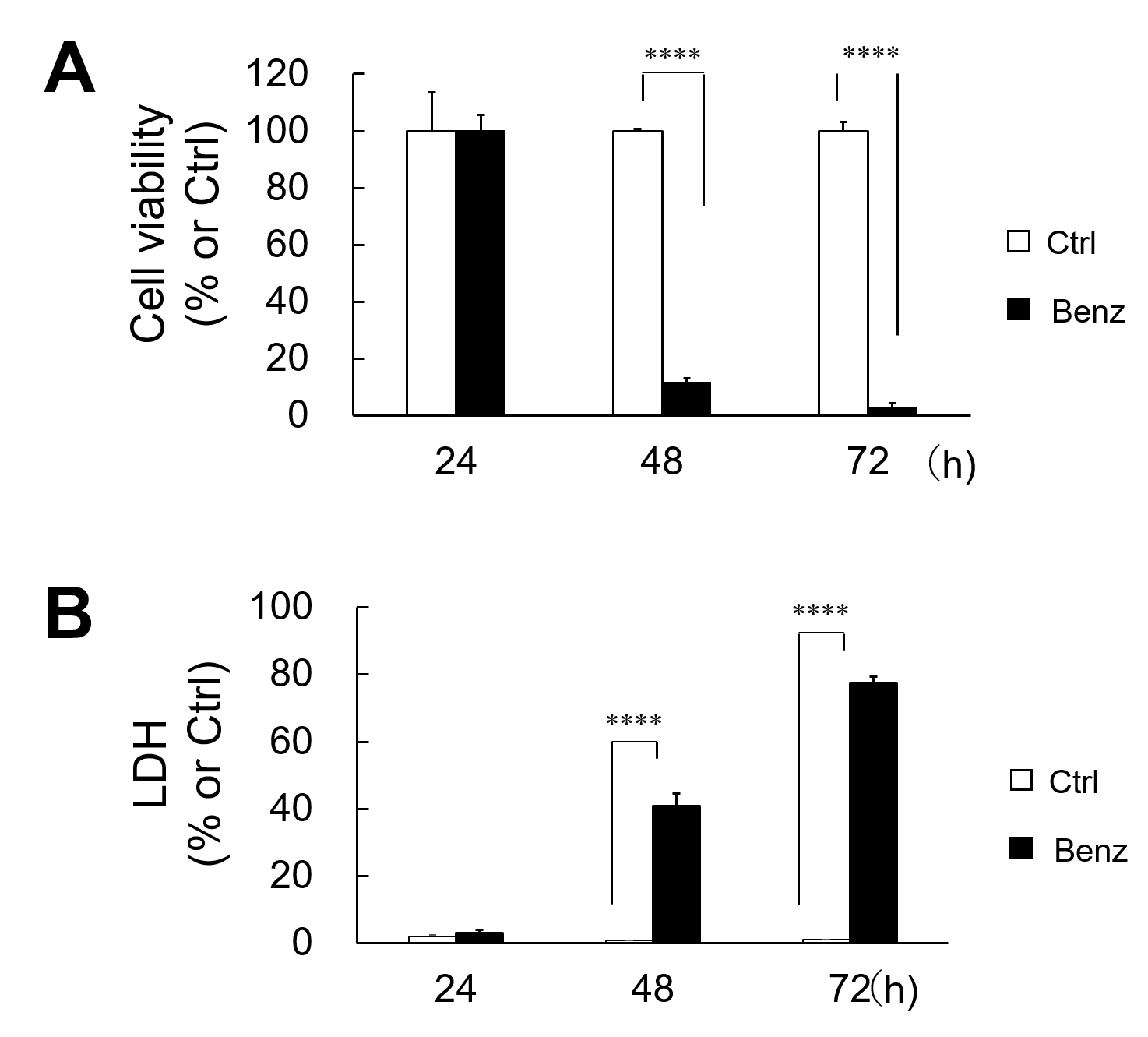

Supplement: Supplementary file 1 [file cancers-12-00523-s001.zip › s1.png]

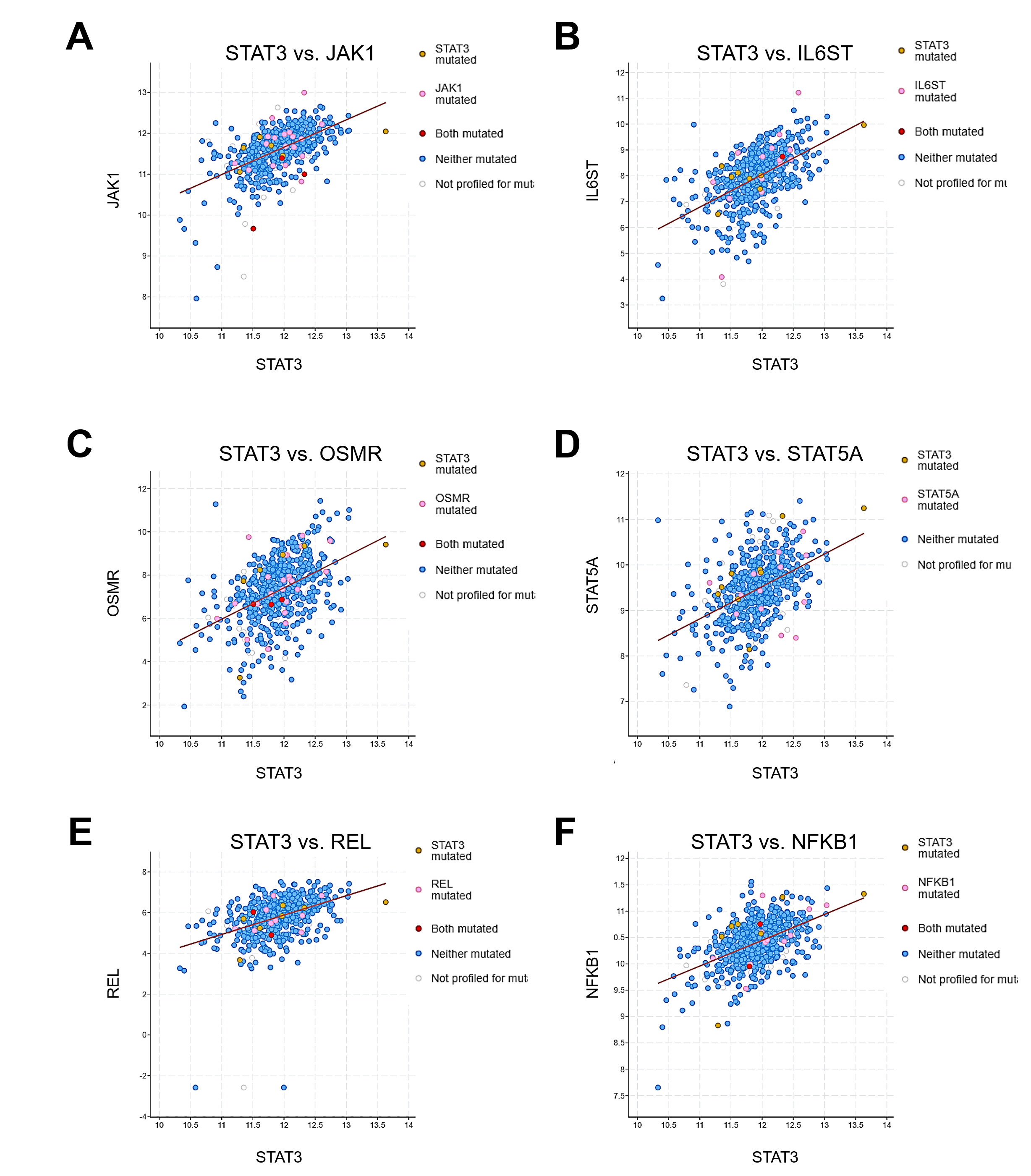

Supplement: Supplementary file 1 [file cancers-12-00523-s001.zip › s10.png]

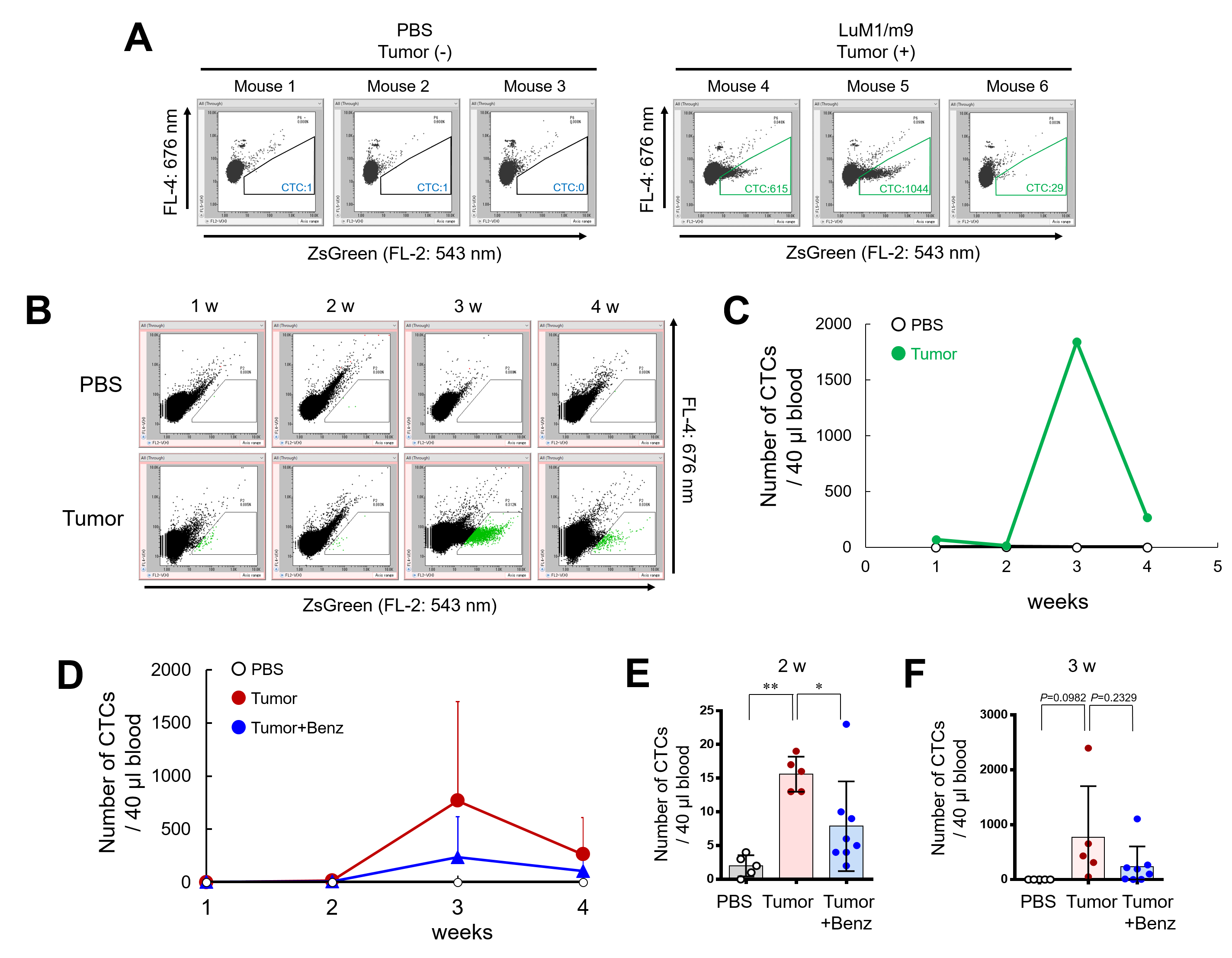

Supplement: Supplementary file 1 [file cancers-12-00523-s001.zip › s11.png]

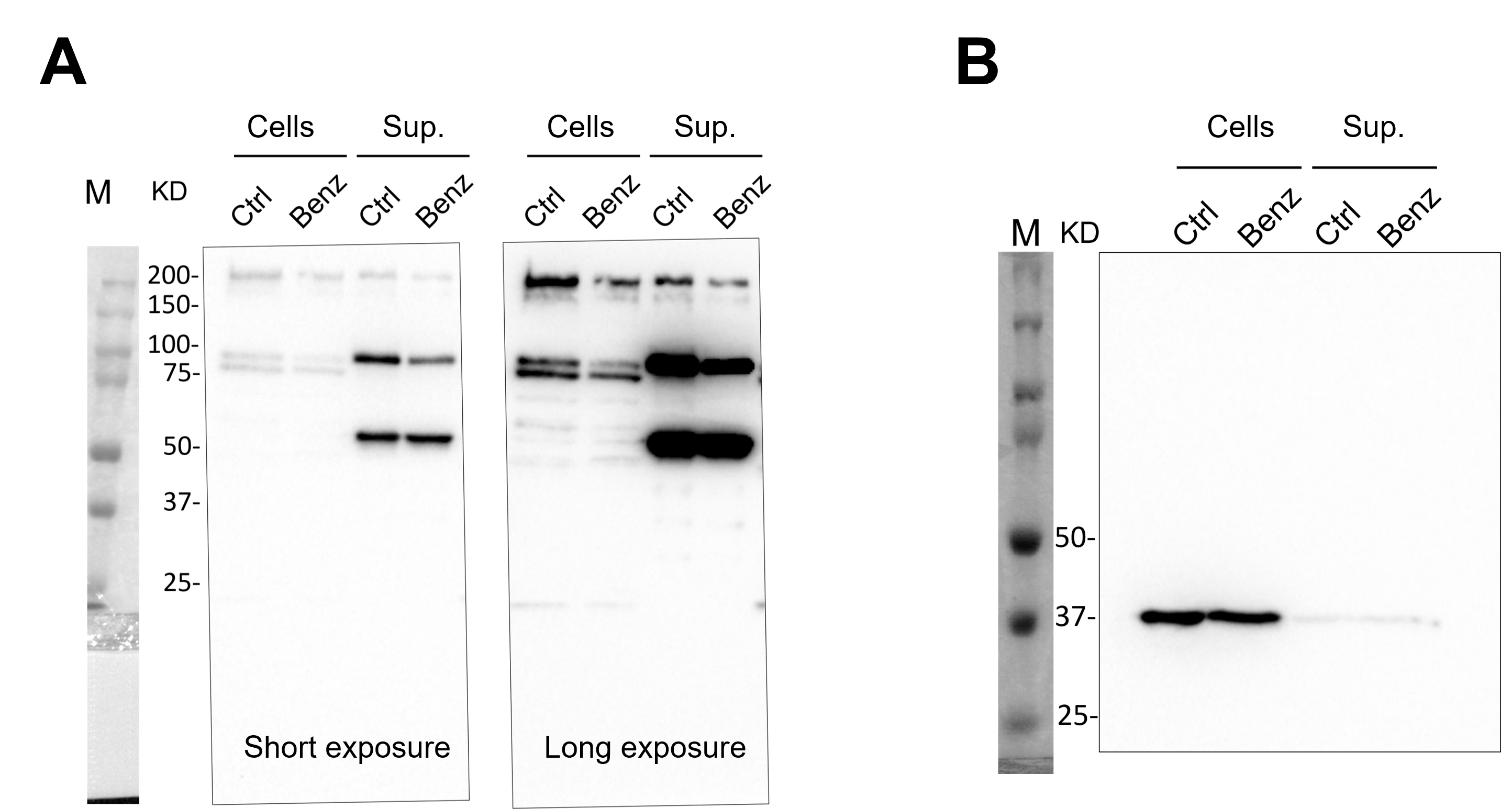

Supplement: Supplementary file 1 [file cancers-12-00523-s001.zip › s2.png]

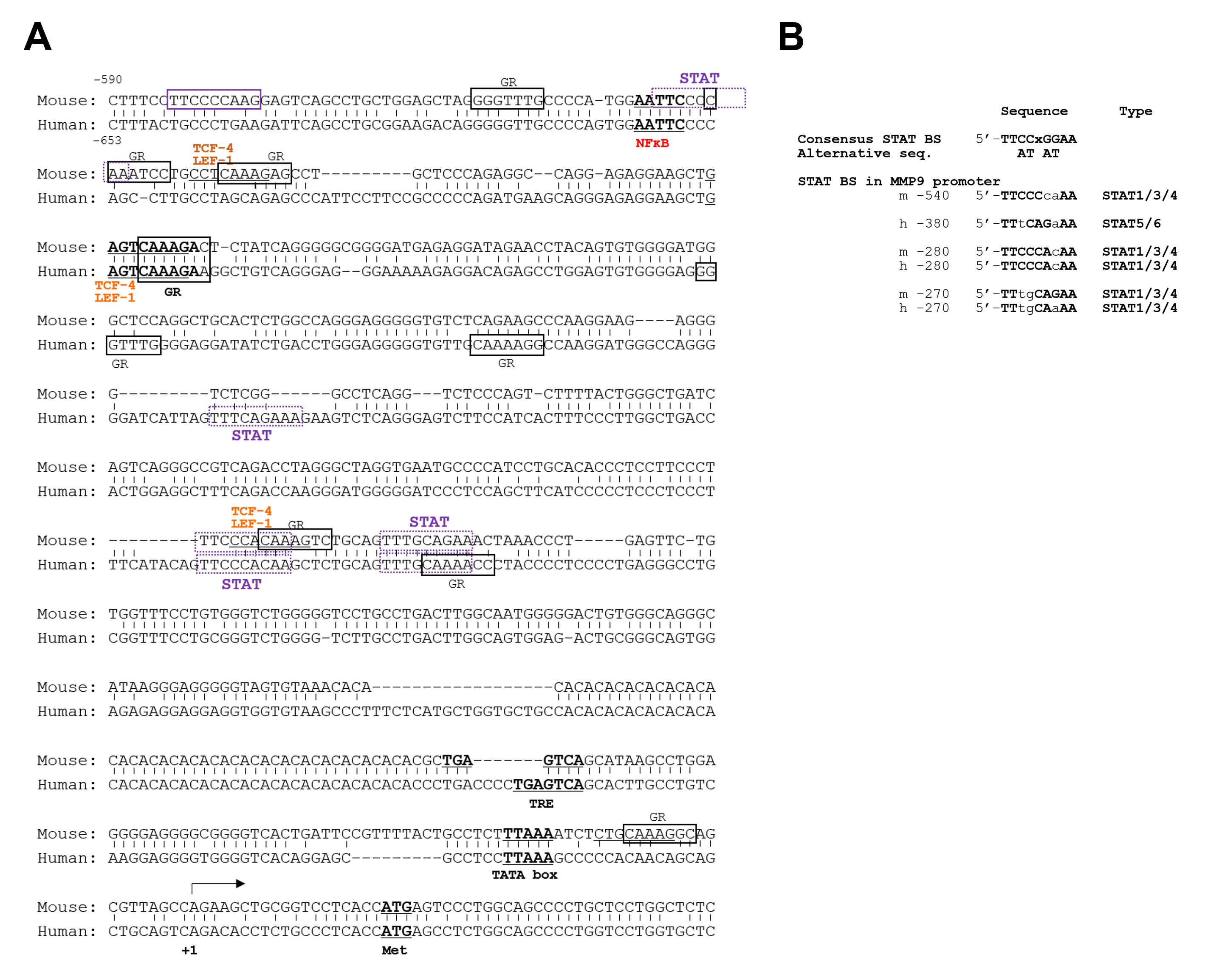

Supplement: Supplementary file 1 [file cancers-12-00523-s001.zip › s3.png]

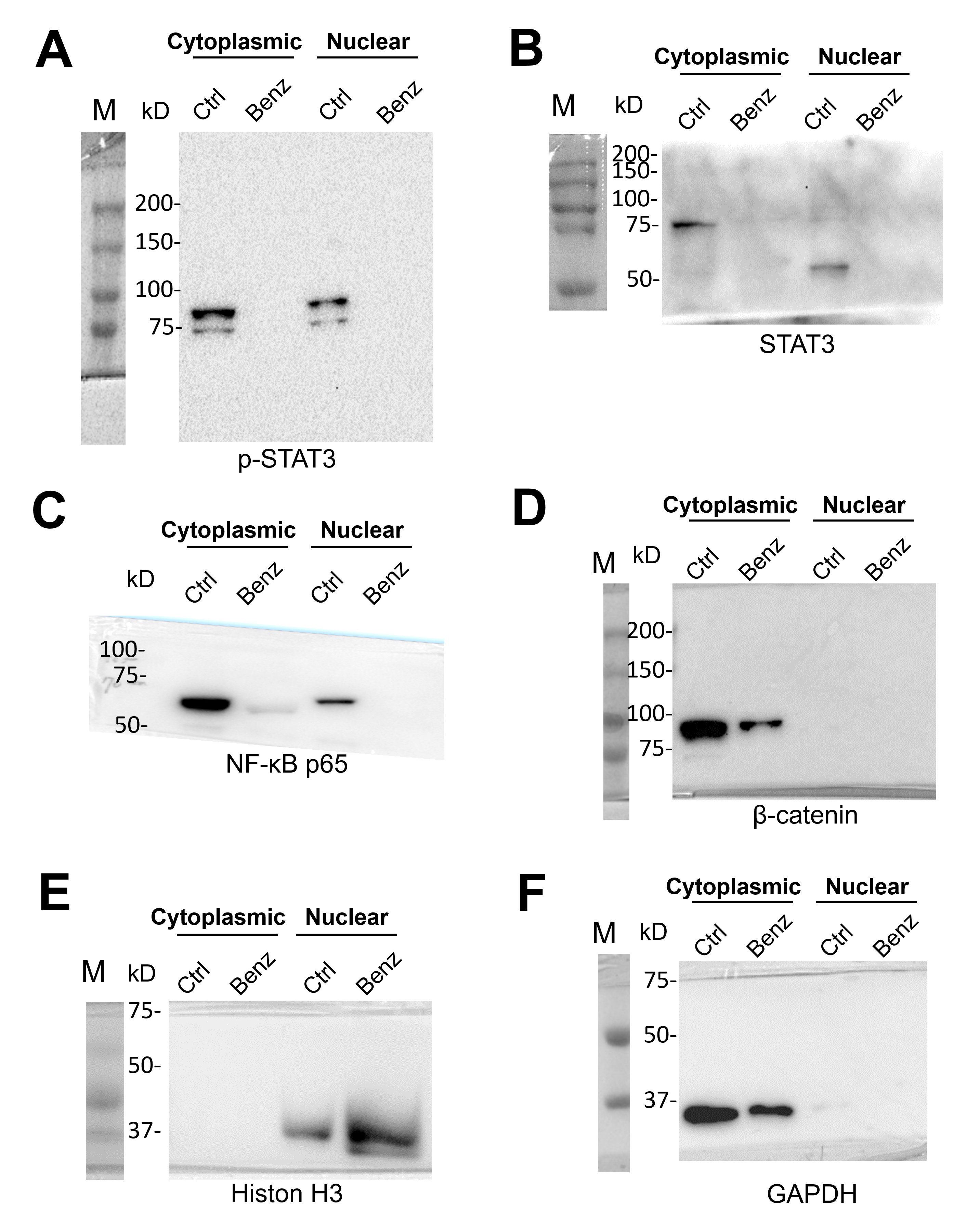

Supplement: Supplementary file 1 [file cancers-12-00523-s001.zip › s4 rev.png]

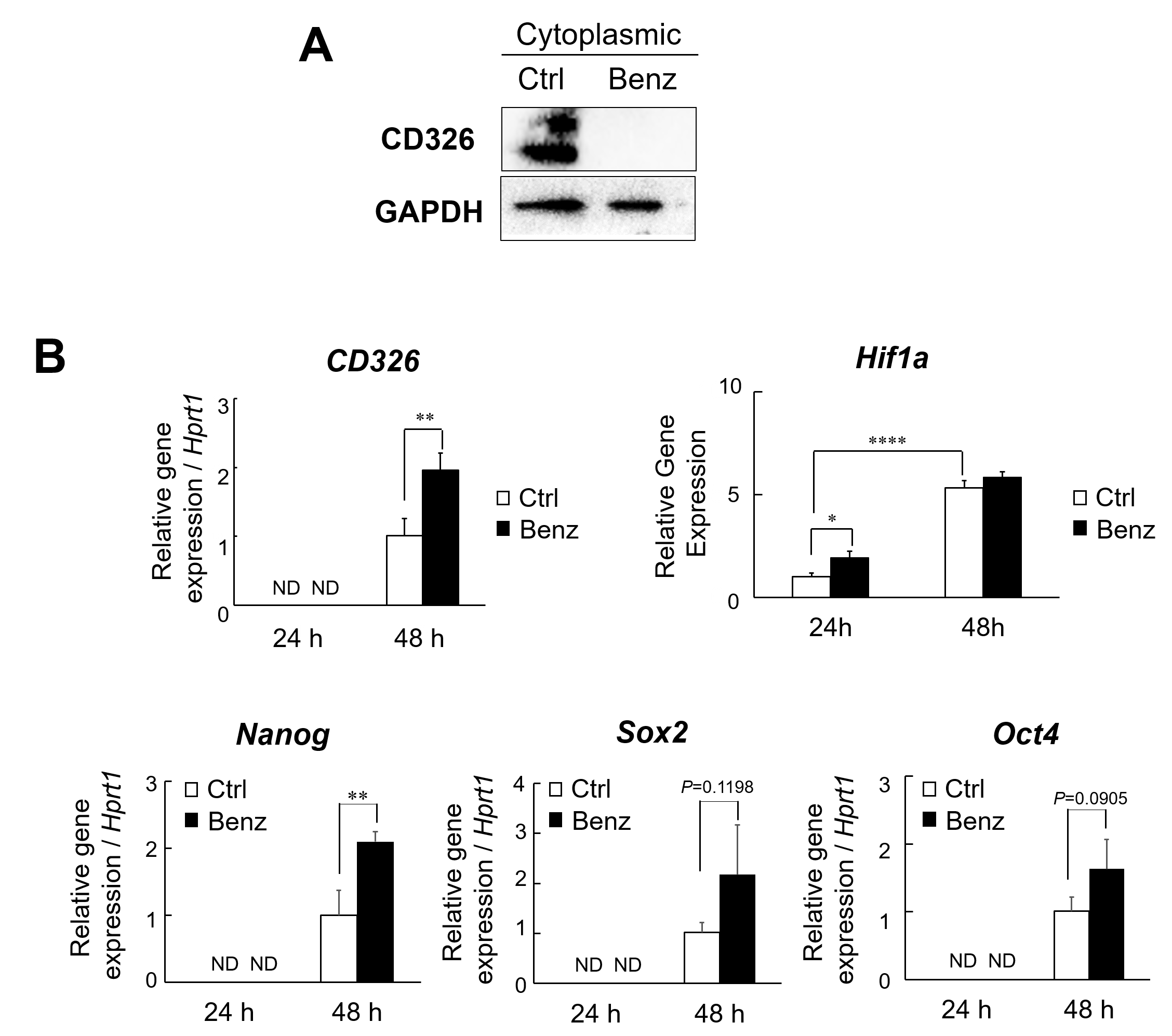

Supplement: Supplementary file 1 [file cancers-12-00523-s001.zip › s5 proof.png]

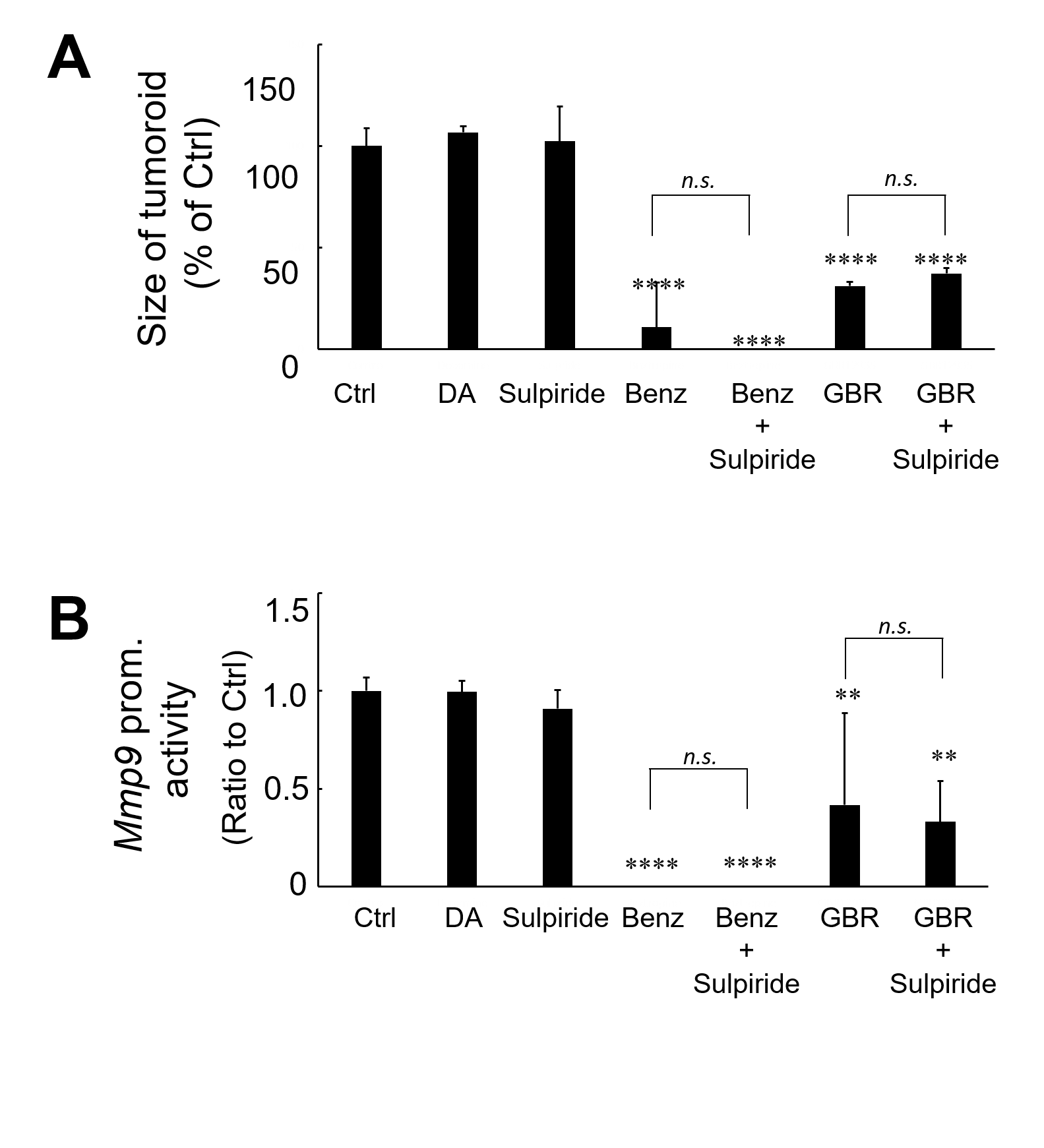

Supplement: Supplementary file 1 [file cancers-12-00523-s001.zip › s6.png]

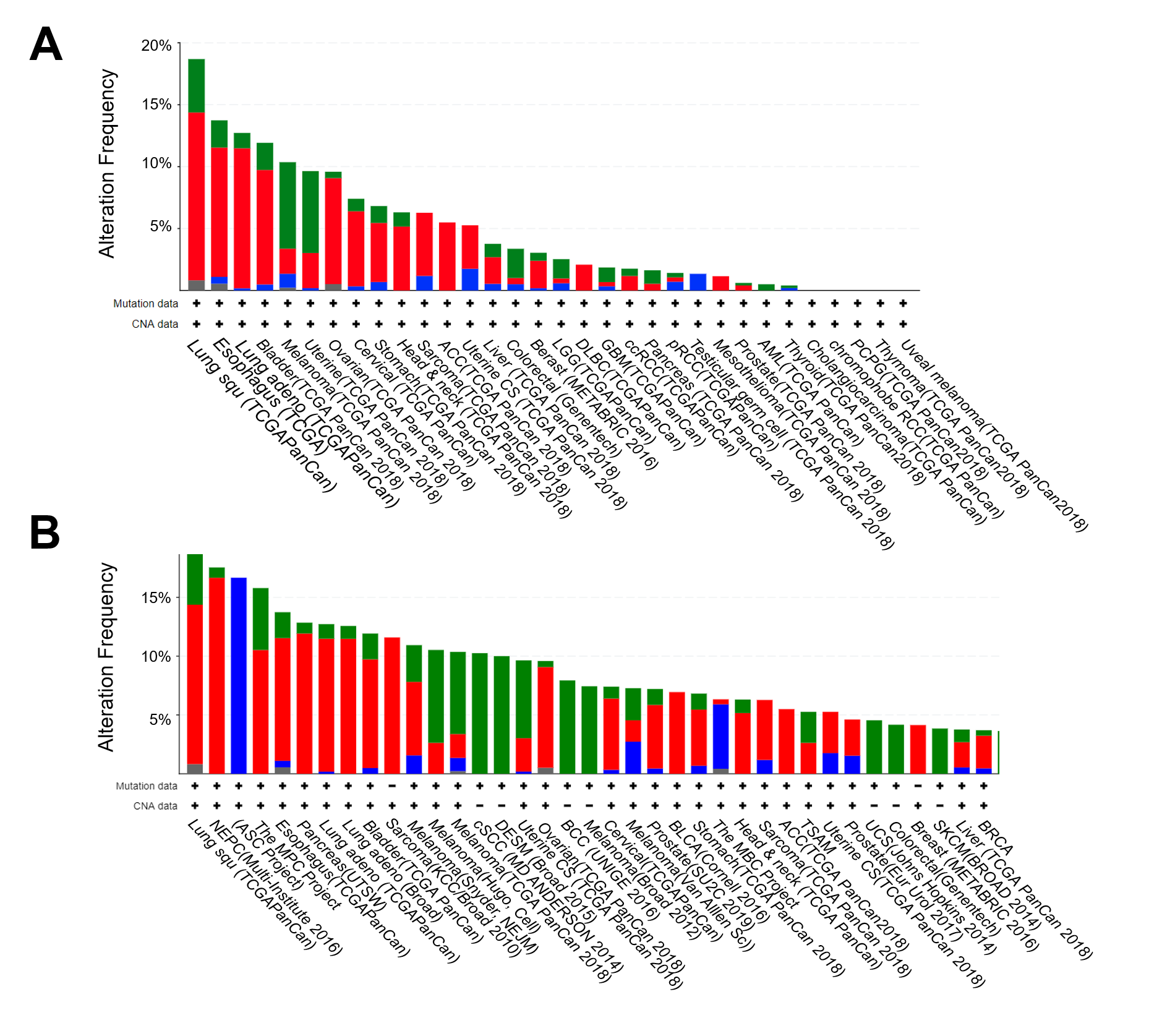

Supplement: Supplementary file 1 [file cancers-12-00523-s001.zip › s7.png]

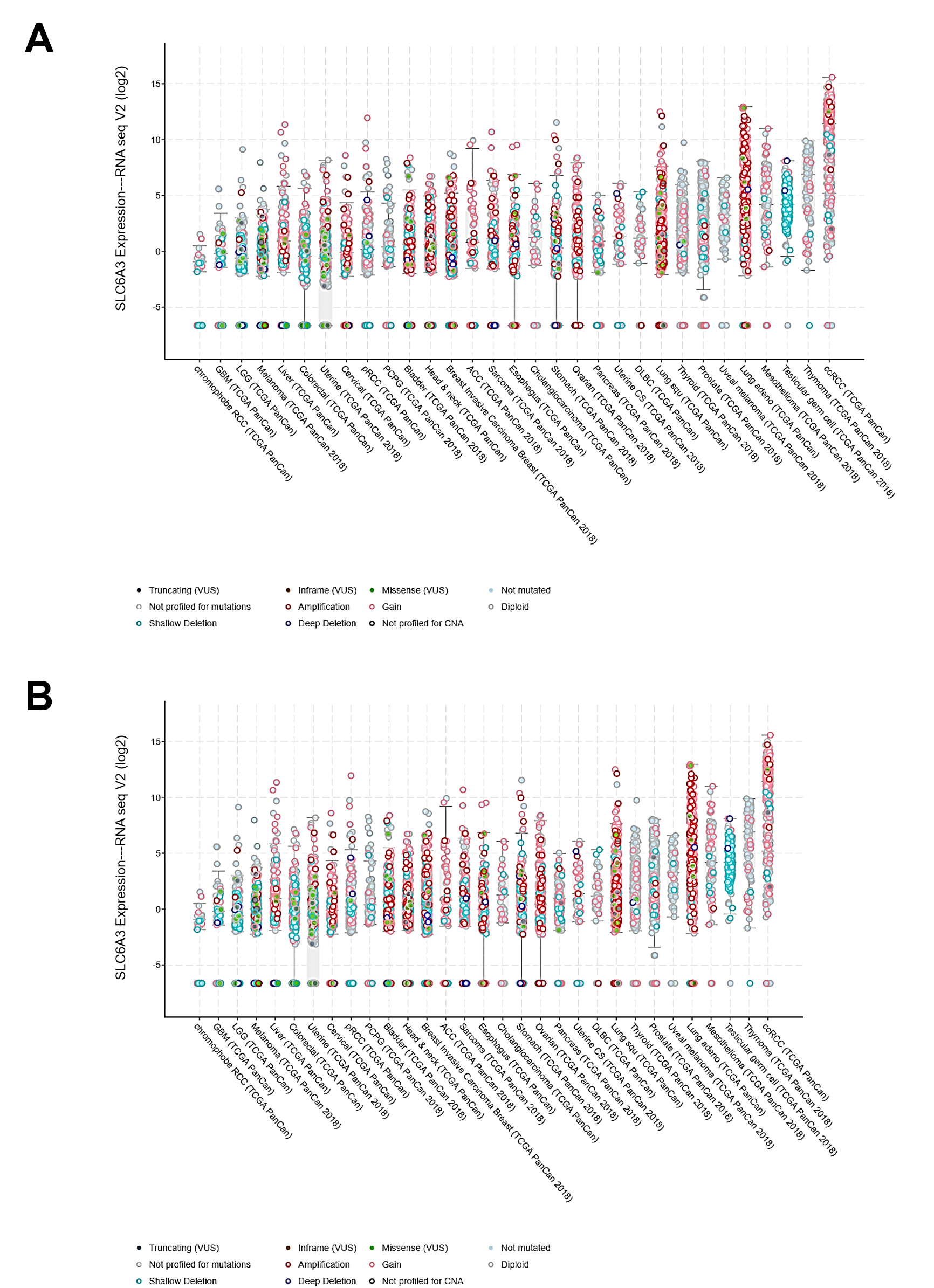

Supplement: Supplementary file 1 [file cancers-12-00523-s001.zip › s8.png]

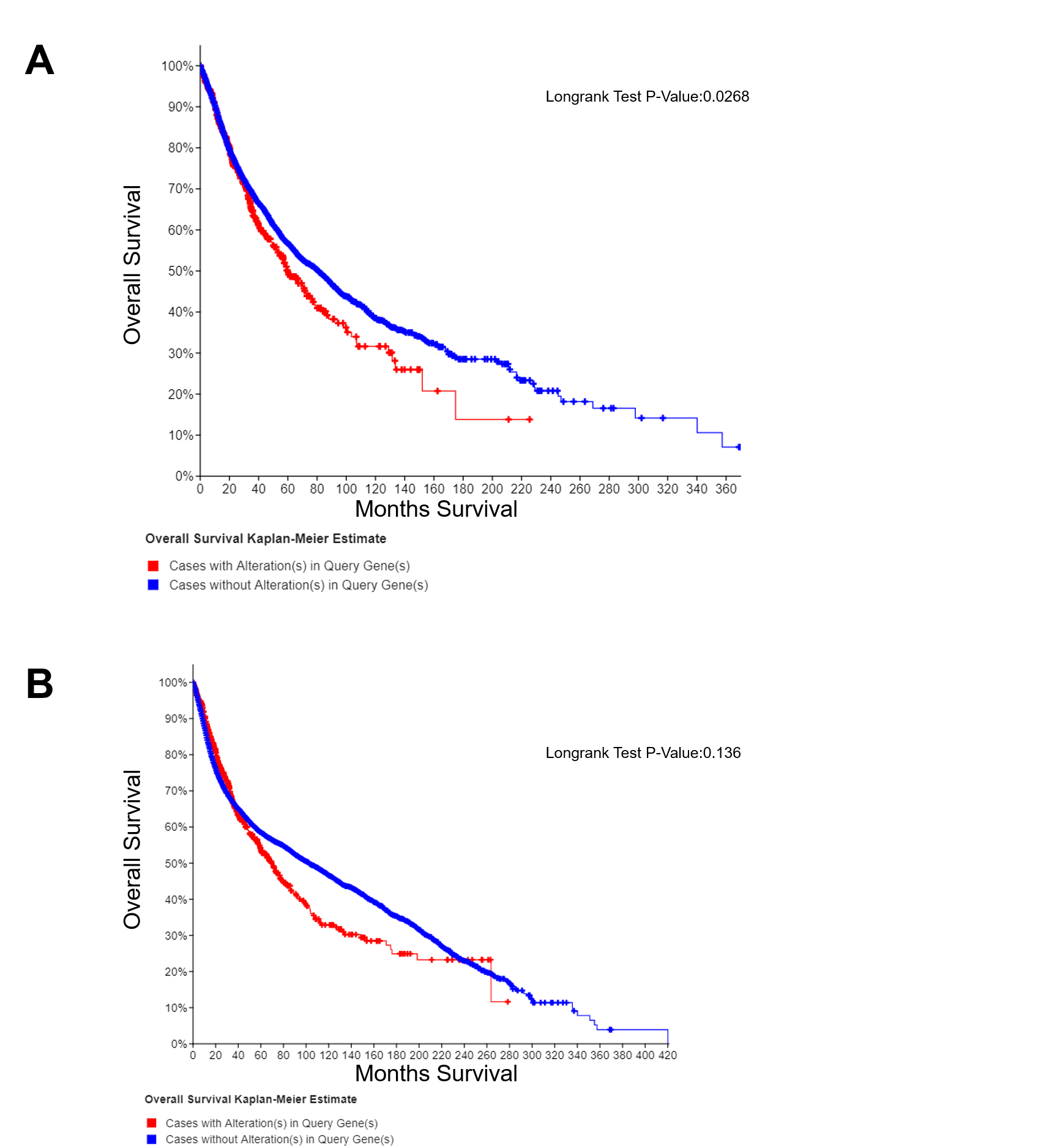

Supplement: Supplementary file 1 [file cancers-12-00523-s001.zip › s9.png]
